# POSTER PRESENTATIONS

**DOI:** 10.1038/sj.bjc.6601056

**Published:** 2003-06-27


**British Journal of Cancer (2003) 88**(Suppl 1), S25 – S54
© 2003 Cancer Research UK All rights reserved 0007 – 0920/03 $25.00
**www.bjcancer.com**

### POSTER PRESENTATIONS

### P1

BONE MARR OW STROMAL C ELL-DER IVED OSTEOPROTEGER IN (OPG) PR OTEC TS B REAST C ANCER CEL LS FROM TRAIL-IN DUCED APOPTOSIS

Hele n L Ne ville-We bbe , Alyson Evans, Co lby Ea ton, Ro bert Cole man , an d Ingu nn Hole n
Cancer Research Centre, Weston Park Hospital, Sheffield

OPG binds to and inhibits TNF-Related Apoptosis Inducing Ligand (TRAIL), thereby potentially increasing the survival of tumour cells normally eliminated by this mechanism. We have investigated whether OPG produced by bone marrow stromal cells (BMSC) isolated from breast cancer patients has the potential to inhibit TRAIL-induced apoptosis of breast cancer cells *in vitro*, thereby increasing tumour cell survival rates.

BMSC were isolated from breast cancer patients (following appropriate ethical approval), and OPG levels determined by ELISA. Breast cancer cells were exposed to TRAIL in the absence or presence of 50% BMSC-conditioned medium, and the levels of apoptotic cell death evaluated.

BMSC were found to produce substantial levels of OPG *in vitro* (45000pg/ml/96hours). In standard medium, TRAIL induced apoptotic cell death in the breast cancer cell line MDA-MB-436, (33%vs 0.6% in control). When the breast cancer cells were exposed to TRAIL in BMSC-conditioned medium containing high levels of OPG, the levels of TRAIL-induced apoptosis were reduced by 50% compared to standard medium.

BMSC isolated from breast cancer patients produce sufficient levels of *OPG in vitro* to protect breast cancer cells from TRAIL-induced apoptosis. Our data suggest that bone-derived OPG may act as a survival factor for breast cancer cells *in vitro*. The *in vivo* significance of these findings remains to be clarified.

### P2

THE ROLE OF CB1 AND CB2 RECEPTORS IN CANNABIS INDUCED APOPTOSIS *IN VITRO*.

Thomas Powles, Jonathan Shamash, Simon Joel, Tim Oliver and Wai Liu. New Drug Study Group, St Bartholomew's Hospital, London, UK.

**Background:** Delta-9 tetrahydocannabinoid (Δ9T) is the activemetabolite of cannabis and causes apoptosis *in vitro*. Both the cannabinoid 1 and 2 (CB1 and CB2) receptors are thought to beinvolved in this process. We have investigated the roleof thesetwo receptors in mediating apoptosis in 3 leukaemiccell lines.**Method:** CB1 and CB2 receptor levels in CEM, HEL92 andHL60 cells weremeasured byusing a novel flow cytometrictechnique involving biotinylation of specific ligands to thereceptors. Cells were cultured with specific CB1 and CB2agonists or with 9T in the presence or absence of the receptorantagonists. Cell parameters including apoptosis were assessed onday 3. **Results:**The levels of CB1 and CB2 receptors are shownin the table. 9T, CB1-agonist andCB2-antagonist (ant) resultedin decreased cellviability and increased apoptosis in all cell linesirrespective of the receptor expression. Increasing concentrationsofCB1- or CB2-antagonists did not reverse the effects of 9T inany of the cell lines (see table).

***Table:*** *Cannabis receptor levels and cell viability data.*

| | R eceptor level | | IC50 9T | %Cell viability | |
|---|---|---|---|---|---|
| | **CB1** | **CB2** | **(∝M)** | **IC50 9T +CB1-ant** | **IC50 9T +CB2-ant** |
| **CEM** | 9.4 ± 3.0 | 1.2 ± 0.3 | 26.5 | 51 ± 12 | 41 ± 8.1 |
| **HL60** | 0.6 ± 0.4 | -0.2 ± 0.5 | 21.1 | 47 ± 6.8 | 45 ± 10 |
| **HEL92** | 5.4 ± 1.0 | 1.0 ± 0.6 | 61.4 | 58 ± 9.9 | 31 ± 4.0 |

**Conclusions:** Contrary to previous reports, our data suggests CB1 and CB2 receptor levels are unimportant in determining cell death. It appears that the mechanism of apoptosis is actually independent of these receptors.

### P3

APOMINE INDUCES APOPTOSIS IN BREAST CANCER CELLS ALTHOUGH THIS IS NOT ACHIEVED THROUGH INHIBITION OF THE MEVALONATE PATHWAY.

Lorraine C. Lowe*, Siddhika G. Senaratne, Kay W. Colston.
St George's Hospital Medical School, London SW17 0RE, UK.

Apomine is a new anti-cancer compound that is a member of the1-1, bisphosphonate ester familyof cholesterol synthesis inhibitors. Our aim was to determine the effect of apomine onbreast cancercellsand to investigate further any growth inhibitory effects found. We have found that apomine causes a significant level of cell deathin MCF-7 and MDA-MB-231 breast cancer cells. This was confirmed as apoptotic cell death using a cell death ELISA andbycaspase7 cleavage in MCF-7 cells. The pro-apoptotic MAPKinasefamily member p38 is also activated in MCF-7 cells with apominetreatment. Amino bisphosphonates, such as zoledronate, induceapoptosis in breast cancer cells by inhibiting the mevalonatepathway which leads to a decrease in the formation of isoprenoidlipids. These are essential for Ras localisation tothe membrane ofthe cell, an action that is required for Ras activation. A decrease inthe production of these lipids leads to impaired Ras localisation andactivation. To investigate whether apomine was acting in a similarmanner we added mevalonate to cell medium while also treatingwith apomine. We found that this did not rescue MCF-7 cells from a pomine induced death. Western blot analysis showed that apominet reatment of MCF-7cells does not impair Ras localisation to the membrane. These results indicate that apomine is not inducing apoptosis by inhibiting the mevalonate pathway and is acting byanother mechanism.We are currentlyinvestigatingwhether thefarnesol X receptor (FXR) may be involved in the action ofapomine on breast cancer cells.

### P4

ZOLEDRONIC ACID INDUCES APOPTOSIS AND INHIBITS ADHESION OF BREAST AND PROSTATE CANCER CELLS VIA INHIBITION OF PROTEIN PRENYLATION

Jonathan P Coxon*, Lisa M Pickering, Kay W Colston
Department of Urology, St George's Hospital, London, UK

Bisphosphonates (BPs) have been shown to have direct effects oncancer cell function- for example, inducingapoptosis of breast andprostate cancer cells *in vitro*, previously only after exposure to highdoses (up to 100∝M), and for long durations (3-4 days). Potent BPs, such as zoledronic acid (ZA), inhibit the mevalonate pathway, which normally allows G-protein prenylation, required for membrane localisation. In this study, were-examinedeffects of ZAon apoptosis and on adhesion of breast and prostate cancer cells. We investigated whether effects on adhesion were altered byco-treatment with analogues of intermediates in the mevalonatepath way: farnesol (FOH), or geranylgeraniol (GGOH). We also compared effects with C3-exoenzyme (C3X), an inhibitor of Rho.

A cell death ELISA was usedto examine apoptosis. To testadhesion, cells were exposed to ZA for 24 hours, then seeded on to dentineslices for 24 hours before being fixed, stained and counted.Cells werealso exposed for 3 hours toeither FOHor GGOH priorto ZA. Effects of ZA were compared to 5μg/ml C3X.

ZA induced apoptosis and inhibited adhesion in breast andprostate cancer cells. Significant induction of apoptosis was seenearlier (1 day), and at lower concentrations (1μM), than previously (similar to adhesion). In MCF-7 cells (breast), inhibition ofadhesion was rescued byFOH, not GGOH. In DU145 and PC-3cells (prostate), only GGO Hrescued inhibition. In all cell lines, C3X significantly attenuated adhesion.

These results show ZA induces apoptosis and inhibits adhesion inthese cancer cells, and suggest that the underlying mechanism involves inhibiting prenylation of proteins, such as Ras and Rho.

### P5

HUMAN BONE MARROW STROMAL CELLS (hBMSC) PROTECT PROSTATE CANCER CELLS FROM TRAIL INDUCED APOPTOSIS

Rachel Nyambo*, Neil A. Cross, Jenny Lippit, Ingunn Holen, A.A. Gordon Bryden and Colby L. Eaton
Academic Unit Urology, Division of Clinical Sciences, University of Sheffield S10 2RX, UK

The bone microenvironment may provide a range of factors that favour the growth and survival of prostate cancer cells. Osteoprotegerin (OPG) is a molecule involved in bone remodellingand isalso a decoy receptor for TRAIL (TNF related apoptosisinducing ligand). The latter activity identifies OPG as a potentialsurvivalfactor. Theaim of the study was todetermine whetherOPG produced by human tumour associated bone marrow stromalcells(hBMSC) could protect prostate cancer cells fromTRAILinduced apoptosis.

hBMSC cultures were generated frombiopsies of prostate cancerbone metastases. Production of OPG by these cells *in vitro* wasdemonstrated by RT-PCR, Western blot and ELISA. When thehuman prostate cancer cell line PC3 waschallenged with TRAIL inthe presence of medium conditionedby hBMSC, the level ofinduced apoptosis was suppressed compared with that observedwith TRAIL in fresh medium. Immuno-precipitation of OPG, andits removalfrom hBMSC conditioned medium, or co-treatment ofcultures with sRANKL, a factor known to bind OPG, reversed theinhibitoryeffects of conditioned media on TRAIL inducedapoptosis.

These data suggest a potential survival mechanism for prostatecancer in bone mediatedby the production of OPG by tumourassociated stromal cells.

### P6

*IN VITRO* INHIBITORY EFFECTS OF LYCOPENE ON HUMAN CANCER CELLS IS ASSOCIATED WITH APOPTOSIS

Debasish Debnath*, Iain Brown, Mrinalini Bilgi, Steven D Heys, Frank Thies, Andrew C Schofield
Department of Surgery, University of Aberdeen, Medical School, Foresterhill, Aberdeen, AB25 2ZD.

The mechanismof action of lycopene, an anticancer carotenoid available in tomatoes, remains unclear. Although lycopene hasshown to induce apoptosis in Jurkat E6.1 malignant T-lymphoblastcells, no such effect has been demonstrated on other cancer cells.

We aimed to evaluate apoptosis by assessing the presence ofapoptotic ladder in cells following *in vitro* exposure to lycopene.

Seven human cancer cell lines [A549 (lung), MCF-7 (ER+vebreast), MDA-MB-231 (ER-ve breast), WiDR (colon), LNCaP(hormone sensitive prostate), PC-3 (hormone resistant prostate)and AGS (stomach)] were exposed to lycopene at a concentrationof 1 μM. The effects were assessed by MTT assays at 24, 48, 72and 96 hours. DNA was extracted from each cell line (followingexposure to lycopene for 24 hours as well as control) andelectrophoresed on a 2% agarose gel.

Results showed that lycopene significantly inhibited (18%-55%) growth of all cell lines ($p<0.05$), irrespective of the hormonal status.The levels of inhibition decreased after 48 hours. Evidence of apoptosis was demonstrated by the presence of DNA fragmentation

Lycopene inhibited the growth of several human cancer celllines, particularly WiDR and LNCaP. Furthermore, the inhibitoryeffect of lycopene appears to be due to apoptosis. Further workusing more specific and quantitative measures of apoptosis will becarried out to validate these results.

### P7

RADIATION-INDUCED MAPK ACTIVATION PRODUCES POLYPLOID TUMOUR CELLS THAT UNDERGO DNA DOUBLE STRAND BREAK REPAIR.

*Andrey Ivanov[1], Mark Cragg[2], Jekaterina Erenpreisa[3] and Tim Illidge[1.1] Cancer Research UK Wessex Oncology Unit and [2]Tenovus Research Laboratory, Southampton, UK. [3]Biomedical Research and Study Center, Riga, Latvia p53 mutant tumor cells respond to genotoxic insults by bypassing the G1checkpoint and halting in G2. Following release from G2 arrest they undergo complex cellular events termed mitoticcatastrophe, whereby mitotic cyclingis suppressed, delayedapoptosis begins and endopolyploid cells are produced. Here wepresent evidence that DNAdamage induced after irradiation, asshown byan increase inthe presence of -H2AX foci, can berepaired in these endopolyploid cells. We studied the kinetics anddistribution of Rad51 protein knownto be important inhomologous recombination DNA repair. Expressionof Rad51 focireached a maximum5-6 days after irradiation, at the peak of endopolyploidy and delayed apoptosis, indicating that selection ofcells for survivalor apoptosis was occurring at this time. Further more, the proportion of Annexin-V positive cells decreasedas the endopolyploid cells continued rounds of DNA replication from day 2-4, indicating that endoreduplication is involved inselecting cells resistantto apoptosis. Using the MEK1 inhibitor U0126, we wereable to inhibit the abrogation of radiation inducedG2 arrest and mitotic catastrophe, with a resultingdecrease in bothdelayed apoptosis and the formation of endopolyploid cells.Resolution of -H2AX foci induced after irradiation was howeveralso inhibited by U0126. Therefore our findings suggest thatendopolyploid cells produced after adaptation of G2 and M-phasearrest appear to have a greater potential for DNAdouble strandbreak repair and are protected from apoptosis suggesting that the endopolyploid fraction may be an important repair compartment.

### P8

INDUCTION OF DIFFERENTIATION AND APOPTOSIS BY APAG-1 IN ACUTE PROMYELOCYTIC LEUKAEMIA CELLS

Johnson Stanslas*, Shiamala D. Manikam, and Andrea L. Holme, Faculty of Medicine and Health Sciences, Universiti Putra Malaysia, 43400 UPM, Serdang, Selangor, Malaysia.

This study was carried out to evaluate the potential of APAG-1, a naturally occurring diterpenoid lactone as a differentiating agent in acute promyelocytic leukaemia cells (APL): HL-60 (late FAB-M2), NB4 (FAB-M3) and NB4-R2 (NB4 cell line made resistant to all-trans retinoic acid (ATRA)). In an earlier study, APAG-1 showed antitumour activity in breast cancer models (Stanslas et al. 2001, *Eur J Cancer*, 37 (Suppl. 6): 169). In this study APAG-1 exhibited 2- and 3-fold increased efficiency in killing HL-60 ($IC_{50} = 2.4 \pm 0.5$ μM) and NB4-R2 ($IC_{50} = 1.5 \pm 0.3$ μM) cells, respectively compared with NB4 cells ($IC_{50} = 4.5 \pm 0.5$ μM) ($p<0.001$).

The induction of differentiation in NB4 cells was assessed by the ability of the cells to produce superoxide, measured by the reduction of nitroblue tetrazolium (NBT) dye. APAG-1 induced a linear kinetic differentiation effect throughout the 96 hr exposure at $IC_{50}$ concentration. Failure of retinoic acid receptor (RAR) antagonist AGN 193109 to reverse the cytotoxic effect of APAG-1 suggests that this compound exerts its effect through an RAR independent signalling pathway to induce differentiation, unlike ATRA.

Morphologically, APAG-1 induced apoptosis and this was later confirmed by the presence of internucleosomal DNA fragmentation of 200 bp ladders on agarose gel electrophoresis. In conclusion, the abilities of APAG-1 to be effective against primary NB4 cells by inducing differentiation and apoptosis, and its killing effect on ATRA-resistant APL cells at a concentration range that is achievable in the plasma i.e. 3 – 30 μM (Stanslas et al. 2001, *Eur J Cancer*, 37 (Suppl. 6): 169) suggest that APAG-1 may be useful in the treatment of primary and ATRA-relapse APL.

© 2003 Cancer Research UK

### P9

REDOX-ACTIVE COBALT COMPLEXES AS SELECTIVE ANTICANCER AGENTS: MECHANISMS OF ACTIVITY

Larissa N. Bubnovskaya*, SergejP. Osinsky, Ilia Ya. Levitin,Andrej L .Sigan, Irina I. Ganusevich, Antonina V. Kovelskaya,Natalia V. Valkovskaya, Luigi Campanella, Peter Wardman Institute of Exp. Pathol. Oncol. Radiobiol., 03022 Kiev, Ukraine; INEOS, Moscow, Russia; University "La Sapienza", Rome,Italy; Gray Cancer Inst., Northwood, UK.

**Aim:**to study mechanisms of biological activity of novel cobaltcomplexes (CoC) displaing anticancer effect.

**Materials&Methods:** CoC of the common formula [Co(acac2en)L2] X where L nicotinamide (AC-30), or *iso*nicotinamide (AC-40), were tested. Murine tumours [Lewislung carcinoma (3LL), melanoma (B16), adenocarcinoma Ca755]were investigated. Pulse radiolysis, electrochemical techniques,biochemical methods and NMR spectroscopy were applied inmodel and in vivo studies. All experiments have been approvedby the regional animal ethics committee.

**Results:** Growth inhibition of primary tumour and metastasesranged from 60 to 80% and from 65 to 99%, respectively. It was shown the reduction of CoC caused the formation of reactiveoxygen species (ROS). ROS selectively attack cellularbiomolecules in primarytumours resulted in the activation of lipidperoxidation, decrease of glutathione content, reduction of theenergy status, increase of DNA unwinding (a marker of single-strand breaks), and decrease of matrix metalloproteinase 2 and 9activity. AC-30 initiated apoptosis in primary tumour by two-phase manner. Theseeffects were not observed innormaltissuesof tumour-bearing animals.

**Conclusion:** ROS formation due to CoC reduction is a keymechanism of CoC effectson cells; ROS affect DNA andcellular matrix and results in the inhibition of tumour growth and metastases.

The study was supported by INTAS (Grant No 00-665).

### P10

HOW DO ZINC-PHTHALOCYANINE DERIVATIVES KILL HUMAN SiHa CELLS DURING PHOTODYNAMIC THERAPY?

S. L. Hankin*[1], D. I. Vernon[1], J. Griffiths[2], J. Schofield[2] & S. B. Brown[1].
Centre for Photobiology and Photodynamic Therapy, [1]School of Biochemistry and Molecular Biology, [2]Department of Colour Chemistry, University of Leeds, Leeds, LS2 9JT.

Photodynamic therapy (PDT)-mediated cell cycle deregulation and apoptosis were evaluated using zinc phthalocyanine derivatives in SiHa cells. Photosensitisers included a zinc phthalocyanine tetra-sulphonic acid (TSPC) and a series of derivatives with amino-acid substituents of varying alkyl chain length and degree of branching; glycine, -alanine, -alanine, butyric, valeric and caproic.

The response was characterised using the Sulforhodamine B assay and by flow cytometry for both DNA cell cycle and dual Annexin V-FITC /PI analysis. All PDT-related experiments involved a 2-hour drug incubation at specific concentrations and illumination with $3J/cm^2$ using a 665nm laser.

An overall trend of increased phototoxicity with amino acid chain length was evident, although phototoxicity was the same order of magnitude as TSPC. PDT resulted in apoptosis, inhibition of cell growth and $G_0/G_1$ cell cycle arrest in a time and dose dependent manner. This novel study has provided evidence for the involvement of cell cycle deregulation during the phthalocyanine PDT-mediated response.

### P11

BIOAVAILABILITYOF NOVEL ANTICANCER CHALCONES IN NUDE MICE BEARING XENOGRAFTS

P. Gibson[1]*, P.M. Loadman[1], S. D.Shnyder[1], P.A.Cooper[1], M. C. Bibby[1], G. Potter[2] and P.C. Butler[2]
[1]Cancer Research Unit, Tom Connors Cancer Research Centre, University of Bradford, Bradford BD7 1DP
[2]Cancer Drug Discovery Group, Schoolof Pharmacy, De Montfort University, Leicester LE1 9BH

Chalcones have shown *in vitro* cytotoxicity by mechanisms which mayinclude interactions with p53 and tubulin. The novelchalcones DMU-120, 135, and 175 have been designed as potential tumour selective anticancer prodrugs1. These compounds are effective at killing human tumour-derived cell lines *in vitro*: 50% inhibition of growth is achieved between 0.005 and 1 μM insensitive cell lines (MCF7 and MDA-468 breast adencocarcinomalines). With the intention of investigating their *in vivo* efficacyagainst xenograft models of human cancer, we undertook a bioavailability study using three lead compounds. Work was doneusingathymic ("nude") mice. Mice were housed and treated according to relevant Home Office guidelines and statutes.

These compounds were found to be very well to lerated, with maximum tolerated dose (MTD) being 200mg/kg in each case. Dosing at MTD, we found by HPLC analysis that the drugs rapidly reached both liver and xenograft (MDA-MB-468) in appreciableconcentrations: 15 minutes after injection, drugs were present at15-170μM in liver and 20-75∝M inxenograft.

Xenograft concentrations remained at up to 10μM for 60 minutes. We present here results which show that, given the provenanticancer properties of chalcones*in vitro*, this class of compoundsshould be progressed to preclinical animal studies.

1Potter, G.A., Butler, P.C. & Wanogho, E. Chalcone TherapeuticAgents, British Patent PCT: GB 01/01341 (2001).

### P12

DESIGN OF TUMOUR-ACTIVATED PRODRUGS THAT HARNESS THE 'DARK SIDE' OF MMP-9

Lesley Young[1], Alberto Di Salvo[1], Agnes Turnbull[1], Jason Lyle[2], Michael C Bibby[2], John A Double[2], Graeme Kay[1], Paul M Loadman[2], David J Mincher*[1]
[1]School of Life Sciences, Napier University, Edinburgh, EH10 5DT, UK; [2]Cancer Research Unit, University of Bradford, BD7 1DP, Bradford, UK.

The aimof this study is to design prodrugs that are converted to potent compounds within the tumour environment by exploitingthe proteolytic action of overexpressed MMP-91. Proptotypes, D-Ala-Ala-Ala-Leu-Gly-Leu-Pro heptapeptide conjugate**(1)** and theD-Ala-Ala-Leu-Gly-Nva-Pro analogue **(2)** have been designed asefficient substrates for tumour-associated MMP-9. Incubation of the non-toxic prodrug **(1)** with human recombinantMMP-9 resulted in cleavage of the heptapeptide motif at the Gly-Leu cleavage 'hot-spot' to afford the residual anthracenyl-Pro-Leu-Glytripeptide conjugate (by LC-MS). Incubation of **(1)** withdiluted (1:500) MMP-9-expressing (by zymography)HT1080human fibrosarcoma tissue homogenates gave rapid (1.8μmolmin-1 g -1) initialmetabolism to the sametripeptide conjugatewhich was further cleaved to the residual,*in vivo-active*, prolinemetabolite NU:UB 31. Introduction of unnatural norvaline (Nva),in **(2)**, in which the -side chain wasmodelled into a deephydrophobic S1 pocketon the enzyme surfaceof MMP-9,directed the cleavage to the N-terminus of the Nva residue, toafford the cytotoxic anthracenyl-Pro-Nva dipeptide metabolite**(3)**. [IC50 3 μM *vs.* intact prodrug (IC50 >100μM) against theMAC15A cellline]. Furthermore, differential metabolism of theprodrugs **(1)** and **(2)**by tumour tissue and non-transformed livertissue is feasible by structural manipulation of the peptide motif.1Mincher D J, (2002) British Journal Of Cancer 86(Suppl 1), S34

© 2003 Cancer Research UK 

**P13**

ANTITUMOUR QUINOLS AS NOVEL INHIBITORS OF THIOREDOXIN SIGNALLING

Jane M. Berry, Andrew D. Westwell, Charles A. Laughton, Charles S. Matthews, Malcolm F.G. Stevens and Tracey D. Bradshaw*
School of Pharmaceutical Sciences, University of Nottingham, Nottingham, NG7 2RD

The antitumour quinols (e.g. AW 464) are a novel seriesofagents found to have selective *in vitro* LC50 activity concentratedin certain colon and renal human cancer cell lines (NCI sixty cellline analysis). Recently a new generation of antitumour quinolshas been found tobe substantially more potent *in vitro* in anexpanded repertoire of cell lines. For example quinol BW 114gives a mean GI50 value of 38.9 nM (sixty cell line analysis) with the most potent activity concentrated in colon HCT 116 (LC5033.1 nM) and renal CAKI-1 (LC50 52.5 nM) cell lines.

COMPARE analysis within the NCI database has led us to suggest the small redox protein thioredoxin as a potential target.Our hypothesis on themechanistic role of thioredoxin has nowbeen experimentally verified using the insulin reduction assay(dose dependent inhibition of bacterial thioredoxin signallingcorrelating with*in vitro* growth inhibition).

Gene microarray studies (HCT 116 cells treated at1µM) provide further verification for thioredoxin as an intracellulartarget (Anne Monks, NCI, personnel communication); amongst apanel of some 10K cancer related genes, only one (thioredoxinreductase) was upregulated more than two-fold. Furthermore, themost highly down regulated gene was CDC2 (CDK1), the kinaseresponsible for cycling of the cell into G2/M. BW 114 was foundto induce a G2/Mcell cycle block (FACS analysis, HCT 116cells). Evidence for an irreversible mode of thioredoxin binding has been suggested from modelling and biochemical thioredoxininhibitory studies.

**P14**

THALIDOMIDE ANALOGUES CC-5013 AND CC-4047 INDUCE T CELL ACTIVATION AND IL-12 PRODUCTION IN PATIENTS WITH BOTH SOLID TUMOURS AND RELAPSED AND REFRACTORY MULTIPLE MYELOMA.

Angus Dalgleish*, Steve Schey, Richard Jones, Kavita Raj, Keith Dredge, Matthew Streetly and Blake Marriott. Guy's and St Thomas'NHS Trust and St George's Hospital Medical School, SW17 ORE

Two thalidomide analogues are currently in ethically approved clinical trials for both solid tumours and multiple myeloma. They were selected for their enhanced ability to inhibit TNFalpha and negative birth defect screens. In vitro studies clearly show stong anti angiogenic activity especially for CC-5013 as well as immuneactivation, with enhanced co-stimulation reported for CC-4047. We therefore sought evidence of immune activation in clinical studies and to correlate such with clinical responses.

In all solid tumour patients and in 17/18 myeloma patients peripheral blood CD4 and CD8 cells greatly increased their expression of the activation and memory marker CD45RO, whilst expression of the mutually exclusive isoform CD45RA decreased. Serum soluble IL-2 receptor levels were increased indicating Il-2 mediated co-stimulation and increased IL-12 levels which is probably monocytic and dendrtic cell derived. This is consistent with enhanced Th1 responses. Enhanced NK activity was also seen in keeping with IL12 production. Importantly, there were NO effects on pro angiogenic factors incuding IL-8, VEGF or FGF, and neither were there any effects seen on serum IL-6 levels.

Clinical responses were seen some solid tumours including 6/13 melanomas and 1 prostate cancer whereas the M -protein response was <25% in 8/18 (44%), >25-50% in 7/18 and >50% in 3/18 patients with myeloma on a dose escalating study with one Complete Response and 2 very close responses.

Both analogues are clinically active and induce strong co-stimulation. The current study strongly suggests that the mechanism of clinical efficacy for CC-4047 in Myeloma is via immune stimulation and not by inhibiting angiogenesis or IL-6 production.

**P15**

A NEW CLASS OF LIGAND FOR HIGH-ORDER DNA STRUCTURE RECOGNITION.

Victoria A. Phillips,* Sharon A. Jennings, Shelley L. Moores, Terence C. Jenkins, Roger M. Phillips, Richard T. Wheelhouse. University of Bradford, BRADFORD, BD7 1DP, UK.

Tetraplex and triplex nucleic acids are important targets for anti-cancer drug design. Biarylpyrimidines bearingω-aminoalkyl substituents have been designed and synthesised as potentially-planar ligands capable of intercalating these high-order structures.

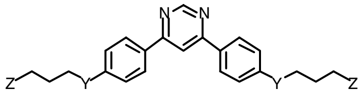

The energy barrier to a planar conformation, $\Delta E$, has been measured as a function of the torsion,Θ, around the inter-annular bond using semi-empirical molecular modelling techniques (Table), supported by X-ray crystallographic data. The influence of functional group Y on electron distribution in the tricyclic system and the propensity to achieveplanarity has also been

| | | $E_{max}$ (Θ = 0°) | $E_{min}$ (Θ = 45°) | $\Delta E$ |
|---|---|---|---|---|
| 1,3-Biphenylbenzene | E / kcal $mol^{-1}$ | 75.21 | 73.28 | 1.93 |
| 4,6-Biphenylpyrimidine | E / kcal $mol^{-1}$ | 99.30 | 98.47 | 0.83 |

The interaction of compounds with poly(dA)· $[poly(dT)]^2$ triplex was assessed by thermal melting experiments. When Z is a simpledialkylamino group, there is a negligible effect on triplex melting ($\Delta Tm1 \leq 1$ °C) and modest duplex stabilisation ($\Delta Tm2 \leq 5$ °C). However, ligands bearing heterocyclic Z -groups show a marked stabilisation ofpoly(dA)· poly(dT) duplex ($\Delta Tm2 \leq 14$ °C) and a specific destabilisation of the triplex structure ($\Delta Tm1 \approx -8$ °C). Substituted biarylpyrimidines have intriguing binding preferences forunusual nucleic acid structures and the compounds examined allexhibited the desirable property of negligible cytotoxicity in a range of human tumour cell lines (IC50 ≥ 100 µM).

**P16**

COMBRETASTATIN A-4-PHOSPHATE (CA-4-P) ACTIVATES NF B IN HUMAN ENDOTHELIAL CELLS.

A.C. Brooks*, C.S. Parkins, C. Kanthou, and G.M. Tozer.
Tumour Microcirculation Group, Gray Cancer Institute, Northwood, Middx., UK HA6 2JR.

The tumour vascular targeting agent CA-4-P inducesendothelial cellular adhesion molecule (CAM) expression, andsubsequent neutrophil adhesion. CAM expression is dependentupon the activation of transcription factors, including NF B,which is known to be activated by Rho GTPases. We haverecently demonstrated Rho GTPase activation by CA-4-P inhuman endothelial cells [Blood **99(6)**2060 (2002)]. The aim ofthis study was to investigate CA-4-P-induced NF B activation.

Nuclearfractions wereobtained fromHUVEC incubatedwith TNF- (100 U/ml), or CA-4-P (0.001 – 0.1 µM). Todetermine the role of Rho in CA-4-P-induced NF B activation,cells were pre-treated with the RhoA inhibitor, C3 exoenzyme,or the Rho kinase inhibitors Y27632 and HA1077. NF B-DNAinteractions were examined using an EMSA, and NF Bcomponents identified using supershift assays.

Following treatment with TNF- or CA-4-P, for 1 hour, twodensely labelled bands were observed on the EMSA blotcorresponding to NF B-DNA complexes. These were inhibitedby excess unlabeled NF B probe, whilst supershift assaysrevealed NF B in the form of a p50·p65 heterodimer. Pre-incubation of cells with C3 exoenzyme prior to CA-4-Pexposure, prevented the formation of the NF B-DNA complexbands, while Y27632 or HA1077 were ineffective. These datasuggest that CA-4-P activates NF Bvia mechanisms involvingRho, but not Rho kinase. This pathway is likely to be importantfor the expression of CAM following vascular targeted therapy.

Acknowledgements: The authors would like to thank Cancer Research UK for funding and OXiGENE Europe AB for supply of CA-4-P.

© 2003 Cancer Research UK

### P17

APAG-1 DERIVATIVES AS ANTITUMOUR AGENTS

Srinivas R. Jada*. Johnson Stanslas, Nordin H. Lajis, Said Saad, Ahmad S. Hamzah, Andrew McCorroll, Charlie Mathews, Malcolm F.G. Stevens, Department of Biomedical Sciences, Faculty of Medicine and Health Sciences, Universiti Putra Malaysia, 43400 UPM, Serdang, Selangor, Malaysia.

APAG-1 is a diterpenoid lactone, isolated from a popular herb found throughout the South East Asian region. Previously it was shown that APAG-1 possesses antitumour activity against *in vitro* and *in vivo* breast cancer models (Stanslas et al. 2001 *Eur J Cancer*, 37 (Suppl. 6): 169). Owing to the potential of APAG-1 as an antitumour agent, we synthesised simple derivatives (SRJ01- 10) of this agent by acetylation, hydrolysis, oxidation, condensation and Heck reactions. Using a 4-day MTT cytotoxic assay we found that the derivatives had $IC_{50}$ values in the submicromolar concentration range in breast tumour cell line, MCF-7, non-small cell lung cancer line, NCI-H460 and prostate cell line, PC-3. Effect of these compounds on the cell cycle progression showed that SRJ01 and SRJ03 induce G1 and G2/M arrest in MCF-7 cells.

Screening of SRJ01 and SRJ03 against the 60 National Cancer Institute (NCI) human cancer cell lines revealed that the 2 compounds failed to exhibit a pronounced antitumour selectivity.

We are currently synthesising newer derivatives of APAG-1 to uncover agent(s) with increased antitumour potency and selectivity.

### P18

INHIBITION OF PROTEASOME FUNCTION AS A MECHANISM OF ANTITU MOUR ACTIVITIES OF NATURALLY OCCURRING LACTONE RING-BASED COMPOUNDS AND THEIR SYNTHETIC DERIVATIVES

Johnson Stanslas*, Radin N.R. Yahaya and Srinivas R. Jada
Faculty of Medicine and Health Sciences, Universiti Putra Malaysia, 43400 UPM, Serdang, Selangor, Malaysia.

This study was conducted as an attempt to uncover themechanism of antitumour activities of naturally occurring APAG-1and goniothalamin (GTN) (Hawariah & Stanslas, 1998,*Anticancer Res.*, 18(6A): 4383; Stanslas et al. 2001, *Eur J Cancer*,37 (Suppl. 6): 169), and their synthetic derivatives. Usinglactacystin as a positive control, APAG-1 and its derivatives, SRJ01and SRJ03, and GTN were shown to significantly inhibit cell freeproteasome activity at concentrations of 25 microM and 50microM, accompanied with a strong association (r=0.75, p<0.05)with cytotoxic effect on breast cancer cell line, MCF-7. APAG-1 and SRJ03 were selected for evaluation of theirantitumour selectivity in a variety tumour cell lines based on theirpreliminary antitumour activity in MCF-7 cells. Using a 4-day MTTcytotoxic assay, parameters of growth inhibition GI50, TGI, LC50and IC50 were derived from dose-response growth inhibition curves.SRJ03 was found to be more active than APAG-1 in inducingcancer cell toxicity (mean IC50 of SRJ03 = 7.0 ± 1.7 microM *vs*mean IC50 of APAG-1 = 26.7 ± 5.8 microM, p<0.05). Overall,SRJ03 was more selective in inhibiting growth and killing of breastcancer cells, MCF-7 and MDA-MB-231. On the contrary, APAG-1was not selective towards breast cancer cells; instead it displayedselectivity towards prostate (DU-145) and ovarian (SKOV-3)cancer cell lines. However, the magnitude of selectivity was 1 logM concentration. The proteasome inhibitory property and the abilityto exert antitumour selectivity by APAG-1 and SRJ03 make thempromising new class of anticancer candidates.

### P19

PHARMACOKINETICS, METABOLISM & GLUTATHIONE REACTIVITY OF SJG-136

Gary P. Wilkinson*, Paul M. Loadman, Terence C. Jenkins, James Taylor, Steve D. Shnyder, Patricia A. Cooper & David E. Thurston
Tom Connors Cancer Research Centre, University of Bradford, All Saints Road, Bradford, West Yorkshire, BD7 3AY

SJG-136 is a novel DNA-crosslinking agent with sequence selective properties. The PBD dimer shows potent *in vitro* cytotoxicity and DNA-stabilising activity, and has been selected for clinical development on the basis of impressive preclinical activity.

The aim of this study was to investigate the *in vitro* & *in vivo* pharmacological characteristics of SJG-136 and to identify any potential metabolites. The proposed reactivity of PBDs with thiol containing macromolecules (e.g. GSH) was also investigated due to the potential influence of this on the pharmacology of SJG-136.

An HPLC method was developed using fluorescence and MS/MS detection. Intraperitoneal administration of SJG-136 to NMRI mice at the MTD (0.2 mg/kg) gave peak plasma concentrations of 336 nM after 30 min and an AUC of 0.34 uM. *In vitro* metabolism studies of SJG-136 in the mouse liver S9 fraction showed rapid drug depletion (< 10% at t0) likely to be due to GSH conjugation. SJG-136 is freely metabolised to a demethylated form in mouse liver microsomes & this metabolite was detected in in vivo samples at low concentrations. This metabolite is not detectible in the S9 fraction suggesting that GSH reactivity inhibits metabolic degradation of the drug. SJG-136 reacts with GSH in a concentration-dependant manner, suggesting increased adduct formation at higher GSH levels. Analytically, these adducts appear to be identical to those found in the S9 fraction.

Such low metabolic potential *in vivo* simplifies the pharmacology of the compound and enhances its therapeutic potential.

All *in vivo* studies complied with UK home office regulations.

### P20

HYPOXIA INDUCED P53 ACTIVATION: THE ROLE OF THE REDOX-SENSING TRANSCRIPTIONAL CO-REPRESSOR CTBP2.

Alexander. H. Mirnezami*; Sandra. J. Campbell; John. N. Primrose; Peter. W. Johnson; Jeremy. P. Blaydes; Cancer Sciences Division, Southampton General Hospital.

Background The p53 tumor suppressor protein has a critical role in the cellular response to stress, and when activated, functions to transactivate genes which induce apoptosis or growth arrest. The oncoprotein Hdm2 is the most significant regulator of p53 activity, and it achieves this partly through inhibition of p53 transactivating ability. In order to investigate the mechanism by which Hdm2 brings about this inhibition, we screened for novel Hdm2 interacting proteins and in this process identified the redox-sensitive transcriptional co-repressor CtBP2 as an Hdm2 binding partner. Methods The interaction was verified using protein binding assays and deletion mutants used to identify the interacting domains. The effect of the interaction on p53 activity was assessed using reporter gene assays. Results Hdm2 participates in a novel interaction with CtBP2 in vitro and in vivo. The acidic domain of Hdm2 and the N-terminus of CtBP2 are necessary and sufficient for this interaction. CtBP proteins undergo an NADH-induced conformational change, which we show results in a loss of Hdm2 binding ability. Recruitment of CtBP2 by Hdm2 results in a promoter selective repression of p53-dependant transcription, and this is abolished by hypoxia-mimicking conditions that increase NADH levels. Conclusions We have identified a novel interaction between Hdm2 and CtBP2, and identified that this interaction is regulated through changes in cellular redox and functions to keep p53 activity in check. This represents a novel mechanism whereby p53 activity can be enhanced by hypoxia, and suggests that inhibition of the Hdm2-CtBP2 interaction may prove a useful target in hypoxia insensitive p53 wild-type tumors.

© 2003 Cancer Research UK

### P21

HYPOXIA UPREGULATES CYTOKINE-INDUCIBLE NOS EXPRESSION IN HUMAN TUMOUR CELLS.

Simendra Singh*, Edwin J. Chinje & Ian J. Stratford
School of Pharmacy, University of Manchester, Manchester (U.K.)

The hypoxic tumour microenvironment is responsible for the selection of malignant clones that overexpress hypoxia-inducible genes responsible for tumour angiogenesis. The cytokine-inducible form of nitric oxide synthase (iNOS) appears to respond to hypoxia stress since its activation is detected in solid tumours. Previous studies indicate that induction of iNOS by cytokines in murine tumour cells (EMT-6) trigger a NO production high enough to reverse hypoxia-induced radioresistance. The aim of this study was to examine the activity of iNOS in aerobic and hypoxic conditions after cytokine exposure in human breast tumour cells.

MDA 231 cells grown to early confluence were exposed to a combination of LPS (50µg/ml) and IFN-γ (100ng/ml) for 24 hr in air & hypoxia. Cultures were then washed out from cytokines and re-incubated for 24, 48, and 72hr. iNOS activity was estimated by conversion of radiolabelled arginine to citrulline and NO.

Combination of LPSand IFN-γ resulted in the up-regulation of iNOS activity up to 48 hr, which remained unchanged at 72 hr. The iNOS induction in air at 48 hr was 6-fold compared to untreated cells. In the absence of LPS and IFN-γ, hypoxia only marginally up-regulated the iNOS level; about 1.5-fold at 48 hr compared to the untreated cells in normoxia. LPS and IFN-γ, treatment in hypoxia resulted in maximal iNOS induction at 48 hr, which was 4-5 fold in comparison to hypoxia alone.

We have demonstrated that hypoxia up-regulates cytokine-induced expression of iNOS in human breast tumour cells. This is of significance since some studies have indicated that NO produced intracellularly in the proximity of radiation targets can be more effective than chemically released NO. Therefore, cytokine modulation of iNOS activity in human tumours can potentially be exploited to increase radiation response.

### P22

USING HORSERADISH PEROXIDASE FOR GENE THERAPY UNDER OXIC AND HYPOXIC CONDITIONS.

Joanna Tupper*, Gillian M. Tozer, Gabi U. Dachs
Gray Cancer Institute, PO Box 100, Mount Vernon Hospital, Northwood, HA6 2JR, UK.

Hypoxic regions within solid tumours tend to be both radio and chemoresistant, resulting in adverse effects on locoregional control in a variety of human and experimental tumours. In this study we have used the horseradish peroxidase enzyme (HRP) for gene directed enzyme prodrug therapy (GDEPT). The HRP enzyme is capable of catalysing the 1 electron reduction of a variety of compounds including indole-3-acetic acid (IAA) and related indoles (Kenten, 1955, Biochem J, 59, 110). In addition HRP can catalyse the conversion of paracetamol to its semiquinone, resulting in formation of *N*-acetyl-*p*-benzoquinone imine (Potter, 1987, Biochem J, 262, 966).

Gene modified cells were grown either as monolayers or tumour cell spheroids, and exposed to prodrugs for 4 or 24h. After 24h exposure, 5mM IAA produced over 95% cell kill in HRP transfectants in both 300 and 700µm diameter spheroids. Similar results were seen with 1Me-IAA, whilst 5Br-IAA showed cell kill after 4h selectively in 700µm diameter spheroids, supporting previous data that it is rapidly activated under hypoxia (Greco, 2001, Mol. Cancer Ther, 1, 151). Paracetamol resulted in selective cell kill of HRP transfectants under both oxic and anoxic conditions. Clonogenic survival was reduced by 95% under anoxia at 5mM, and 8mM under normoxia.

These data show that the use of the HRP enzyme for GDEPT shows continued promise for selective therapy in solid tumours, with a range of potential prodrugs suitable for hypoxia targeting.

This work was funded by Cancer Research UK.

### P23

ENHANCEMENT BY INDOLE-3-ACETIC ACID (AUXIN) OF OXIC AND HYPOXIC PHOTODYNAMIC THERAPY

Lisa K. Folkes* and Peter Wardman, Gray Cancer Institute, Mount Vernon Hospital, Northwood, Middlesex, HA6 2JR, UK.

Photodynamic therapy in cancer therapy generally requires the excitation of oxygen to highly reactive singlet oxygen to kill cells. Tumours commonly exhibit low oxygen tensions. Addition of indole-3-acetic acid (I) to mammalian cells photosensitised with phenothiazinium dyes dramatically increases toxicity compared to the dyes alone, both in aerobic and hypoxic systems.

Toluidine blue ($TB^+$), used in cancer diagnosis, and methylene blue ($MB^+$), used in experimental photodynamic therapy have been investigated. Tissue penetration of light occurs at the high wavelength at which these dyes absorb. Excitation of the dye results in a triplet state that oxidises I to a radical cation which rapidly decarboxylates to a skatoyl radical. In hypoxia this may be involved in toxicity whilst in aerobic conditions it adds oxygen leading to a variety of products. These include methylene oxindole a highly toxic species reactive towards cellular nucleophiles.

Mouse fibroblast V79 and human breast MCF7 cells were incubated with 2 µM dye ± 0.1 mM I and illuminated at 630 or 660 nm (LEDs) either in air or in 1% oxygen. Cells were allowed to grow into colonies and surviving fractions (SF) calculated.

| Dye | Light dose | V79 cells SF in 1% $O_2$ | V79 cells SF in air | MCF7 cells SF in air |
|---|---|---|---|---|
| $TB^+$ | 1.6 J/cm$^2$ | 0.86±0.015 | 0.56 ± 0.12 | 0.91±0.005 |
| $TB^+$ + I | 1.6 J/cm$^2$ | 0.011±0.003 | 0.005±0.003 | 0.007±0.003 |
| $MB^+$ | 1.1 J/cm$^2$ | - | 1.03 ± 0.002 | 1.06 ± 0.08 |
| $MB^+$+ I | 1.1 J/cm$^2$ | - | 0.05 ± 0.035 | 0.002±0.001 |

The results show that addition of IAA at achievable non-toxic concentrations in man could markedly increase the efficacy of some photodynamic therapy treatments. Rational design of photosensitizer and IAA analogues is possible based on thermodynamic and kinetic properties.

*This work is supported by Cancer Research UK*

### P24

IMMUNODETECTION OF GLUT-1 AND NQO1 (NAD(P)H:Quinone oxidoreductase) IN SUPERFICIAL BLADDER CANCER.

Palit V* Basu S, Bibby MC, Shah T, Gill JH and Phillips RM.
Cancer research unit, University of Bradford, Bradford

**Introduction:** Elevated levels of reductase enzymes in conjunction with tumour hypoxia are two key parameters that will influence the efficacy of bioreductive drugs such as Mitomycin C. The enzyme NQO1 has been implicated in the activation of Mitomycin C and the glucose transporter GLUT-1 is known to be induced under hypoxic conditions.

**Aims:** To determine the relationship between NQO1 and GLUT-1 protein expression in superficial transitional cell cancer (TCC) of the bladder.

**Methods:** Sections from 20 paraffin embedded tissues representing superficial (pTa/pT1) and muscle invasive (pT2) TCC were cut and stained for NQO1 and GLUT-1. Slides were scored by three independent investigators and graded as low, moderate or high depending upon the extent and intensity of staining.

**Results:** Staining for NQO1 was cytoplasmic and confined to tumour cells with superficial tumours (n=12) having higher levels than muscle invasive (n=8) tumours. Staining for GLUT-1 was predominantly membranous with moderate to high levels of GLUT-1 present in superficial tumours (the majority of pT2 tumours have low levels of GLUT-1). In papillary tumours, intense GLUT-1 staining was found on the luminal surfaces of the tumour with no staining seen in cells residing close to blood vessels. NQO1 was present in both GLUT-1 positive and negative regions of the tumour.

**Conclusions:** Elevated levels of both NQO1 and GLUT-1 were found in superficial bladder TCC. The finding that many papillary type tumours have intense regions of hypoxia (GLUT-1 positive) offers potentially significant opportunities for hypoxia mediated therapeutic intervention.

© 2003 Cancer Research UK

### P25

AMINE REDUCTION PRODUCTS ARE KEY BYSTANDER METABOLITES WHEN CB 1954 IS ACTIVATED BY *E. coli* NITROREDUCTASE.

Nuala A. Helsby*, Dianne Ferry, Adam V. Patterson and William R. Wilson. Auckland Cancer Society Research Centre, University of Auckland, Private Bag 92019, Auckland, New Zealand.

The dinitrobenzamide aziridine CB 1954 is metabolised by *E. coli* nitroreductase (NTR) to an equimolar mixture of the corresponding 2- and 4-hydroxylamines. The 4-hydroxylamine is a potent DNA crosslinking agent, and is considered to be the major cytotoxic metabolite when NTR/CB 1954 is used in GDEPT (1). In this study we re-assess the bioactivity of CB 1954 metabolites, and examine their relative contribution to the bystander effect (killing of cells that lack NTR) when they are formed within 3D cell cultures (multicellular layers, MCL). NTR-transfected V79 cells were incubated with CB 1954, as single cell suspensions, and the extracellular medium was fractionated by HPLC and bioassayed against UV4 (ERCC-1 deficient) cells. This revealed four metabolites, identified by mass spectrometry as the 2- and 4-hydroxylamine and their corresponding amines. As expected, the 4-hydroxylamine was the major cytotoxic metabolite, but the other three species also showed significant bioactivity. Single cell suspensions of NTR transfected SiHa cells gave a similar metabolic profile. When CB 1954 was added to the donor side of the MCL (NTR transfected SiHa cells) the metabolites detected included the 4-hydroxylamine as well as the 2- and 4- amines. However, the only metabolites appearing on the other side of the MCL were the 2- and 4-amines. These results suggest that the amine metabolites are significant contributors to the bystander effects of CB 1954 following NTR activation in GDEPT, especially at long diffusion distances from NTR vectors.

(1) Bridgewater JA, Knox RJ, Pitts JD, Collins MK, Springer CJ (1997). *Hum Gene Ther* 8: 709

### P27

QM/MM COMPUTATIONAL STUDY OF INTERACTION OF NQO1 WITH BIOREDUCIBLE INDOLEQUINONES

Shelley L. Moores*, Stephen W. Doughty, Roger M. Phillips, Terry C. Jenkins

*YCR Laboratory of Drug Design*, Tom Connors Cancer Research Centre, University of Bradford, Bradford, West Yorkshire BD7 1DP

NQO1 [NAD(P)H: quinone acceptor oxidoreductase 1, or DT-diaphorase] can bioreductively activate prodrugs, including quinones, to active drug species while using NAD(H) or NAD(P)H as an electron donor (co-substrate). As levels of NQO1 are elevated in tumours compared to normal tissues, this system is currently being exploited for chemotherapeutic gain in enzyme-directed bioreductive drug approaches, particularly ADEPT, GDEPT and ENACT.

QM/MM modelling techniques and QSAR analysis have now been used to examine linkages between molecular properties (e.g. activation energy, LUMO) for a series of bioreducible indolequinones [Phillips, R.M. *et al.* (1999) *J. Med. Chem.* **42** 4071] and their substrate activity ($k_{cat}$, $K_m$) towards NQO1. This relationship can be used to predict activity for other candidate agents and thereby assist the overall drug design process.

For the first time, (i) correlation between drug properties and potency, and (ii) the use of molecular probes within the active reducing enzyme pocket provide a firm structural basis for the observed substrate preferences. This pharmacophore-based approach offers a powerful tool for the rational identification of new and active classes of bioreducible prodrugs for NQO1.

### P26

FACTORS INFLUENCING HUMAN CANCER CELLS RESPONSE TO MITOMYCIN C.

Milène Volpato*, Paul M. Loadman, Roger M. Phillips.

Cancer Research Unit, Bradford University, BD7 1DP Bradford.

Predicting tumour response to a specific treatment is an important issue for clinicians and researchers. Mitomycin C (MMC) is a bioreductive prodrug routinely used in clinic, which requires enzymatic activation. Several reductases can metabolise MMC and DT-diaphorase was thought to be the main activating enzyme. Correlation between tumour response and enzyme activity remains controversial. But the mechanism of action of MMC appears to be too complex to enable forecasting response on the basis of one enzyme activating system. The knowledge of drug metabolism may provide a better indicator of response. The aim of this study is to investigate how factors such as MMC metabolism rates and DNA damage induction and repair could influence response of cell lines to MMC. Cells lines have been characterised by their sensitivity to MMC and the activity of DTD and P450 Reductase. H460, HT29 and RT4 have respective $IC_{50}$ values of 0.77, 4.60 and 30.20 µM and respective (DTD;P450R) activities of (353;37), (347;10) and (134;11) nmol/min/mg. MMC metabolism rates were assessed (HPLC): MMC has a half life of approximately 25 min in H460 cells, 33 min in HT29 cells and 17.8 min in RT4 cells. The induction of cross-link and repair (COMET assay) was also analysed. 70% of DNA cross-links were obtained in H460 cells treated with 5 µM of MMC and 20% were repaired after 6h. Preliminary studies in A549 cells, which are as sensitive as HT29 cells the extent of damage was less (50%) and repair was faster and more extensive. In the resistant RT4 cells, no DNA cross-link was detectable. This study clearly suggests that at least two pathways could lead to MMC resistance: repair or detoxification mechanisms. Ongoing studies are investigating these pathways further.

### P28

CHARACTERISATION OF THE *IN VIVO* HOLLOW FIBRE ASSAY FOR THE ANALYSIS OF DNA DAMAGE USING THE COMET ASSAY.

Marie Suggitt*, Michael C. Bibby. Tom Connors Cancer Research Centre, University of Bradford, Bradford, BD7 1DP.

The *in vivo* hollow fibre assay (HFA) is used as a screening model by the National Cancer Institute (NCI) to prioritise potential anticancer agents for secondary xenograft testing. This study investigates whether the HFA can be used as a short-term assay to demonstrate drug-target interactions *in vivo*, namely DNA damage.

Initially A549 lung carcinoma cells were seeded into hollow fibres at various cell densities in order to characterise optimal cell growth both *in vitro* and *in vivo* over 5 days. Following *in vitro* studies of monolayer and hollow fibre cultures revealing DNA crosslinking of A549 cells with mitomycin C (MMC), hollow fibres were seeded with A549 cells (1 x $10^6$cells/ml) and implanted into NMRI mice. On day 4 MMC was administered intraperitoneally (*i.p*) at its maximum tolerated dose (MTD) (6mg/kg). Fibres were removed at 2 hours and cells retrieved for DNA analysis using the comet assay.

Methods of cell preparation for comet analysis were developed throughout the study. Home Office guidelines for the welfare of animals were adhered to throughout the study.

Results revealed statistically significant differences between the mean tail extent moment (TEM) values of treatment and control groups at both *i.p* and *s.c* sites ($p$ = <0.001). These values revealed MMC-induced DNA crosslinking in 55% and 49% of cells at *i.p* and *s.c* sites respectively.

In conclusion these data demonstrate that the HFA can be used as an *in vivo* preclinical model in anticancer drug development for studying the pharmacodynamic effects of the standard agent MMC. This assay is now being used to investigate novel potential DNA interactive compounds.

© 2003 Cancer Research UK

### P29

IMMUNOHISTOCHEMICAL ANALYSIS OF NQO1 AND CYTOCHROME P450 REDUCTASE IN HUMAN SUPERFICIAL BLADDER CANCERS: RELATIONSHIP WITH CLINICAL RESPONSE TO MITOMYCIN C.

Basu S *, Martin SW, Seargent J , Loadman PM , Gill JH , Shah T, Puri R and Phillips RM
Corresponding address: S. BASU; Cancer Research Unit, University of Bradford, Bradford BD7 1DP, UK

**Introduction & aims:** Mitomycin C (MMC) undergoes bioreductive activation by cellular reductases particularly by the enzymes NQO1 and cytochrome P450 reductase (P450R). Considerable controversy surrounds the issue of whether tumour response can be predicted on the basis of tumour enzymology. Our aim in the study was to predict the response of superficial bladder cancers (SBC) to MMC in the clinic on basis of the immunohistochemical distribution of NQO1 and P450R in the tumours.

**Methods:** In this retrospective study, tumour response to MMC (time to first recurrence) was compared to immunohistochemical localisation of NQO1 and P450R in 102 human SBC (pTa & pT1) specimens. Sections were stained for human NQO1 and P450R and graded according to staining intensity and distribution by three independent investigators.

**Results:** The majority of tumours (45%) had high levels of NQO1 and staining was confined to the cytoplasm of tumour cells In contrast, P450R levels in all specimens examined showed moderate to high staining in both cytoplasm and stroma. Clinical response to MMC (either single dose or course) was independent of both NQO1 and P450R expression in tumours.

**Conclusion:** The results of this retrospective study demonstrate that the response of SBC to MMC cannot be predicted on the basis of NQO1 and P450R levels. This study has also demonstrated that NQO1 may be a good target for therapeutic intervention in SBC.

### P30

DOES NQ01 [NAD(P)H QUINONE OXIDOREDUCTASE] GENOTYPE INFLUENCE THE RESPONSE TO MITOMYCIN C IN SUPERFICIAL BLADDER TUMOURS?

Basu S *, Martin SW , Brown JE , Loadman PM , Flannigan GM Shah T Puri R and Phillips RM
Corresponding address: S. BASU; Cancer Research Unit, University of Bradford, Bradford BD7 1DP, UK

**Introduction & aims:** The enzyme NQO1 has been suggested to play a role in the bioreductive activation of Mitomycin C (MMC). The gene encoding for NQO1 is polymorphic and a 609C>T variant results in loss of NQO1 enzyme activity. In view of the potential role of NQO1 in the mechanism of action of MMC, the genotype of patients undergoing MMC therapy may have a significant bearing on efficacy. Our aim in this study was to determine **if** the response of superficial bladder cancer to MMC can be forecast on the basis of NQO1 genotype status.

**Methods:** Following ethical approval, genomic DNA was extracted from 84 paraffin embedded human bladder tumours specimens. NQO1 genotype was determined by PCR-RFLP techniques, which involved the use of two rounds of PCR amplification using a nested primer strategy. PCR products were digested with *Hinf* 1 and genotyped on the basis of the restriction fragments produced.

**Results:** Out of 84 patients, 51 (63.1%) were wild type, 29 (34.5%) were heterozygous, while 2 (2.4%) were homozygous mutants. The clinical response to MMC (time to first recurrence) failed to show any association with NQ01 genotype status.

**Conclusions:** The response of bladder cancer patients to MMC cannot be forecast on the basis of NQO1 genotype status. These results suggest that NQO1 plays a limited role in determining the final outcome of MMC chemotherapy.

### P31

'PING-PONG' FLAVIN REDUCTION IN NQO2: MECHANISM STUDIES AND BIOREDUCTION

Terry C. Jenkins*, Shelley L. Moores*, Stephen W. Doughty, and Richard J. Knox[1]
*YCR Laboratory of Drug Design*, Tom Connors Cancer Research Centre, University of Bradford, West Yorkshire BD7 1DP; [1]Enact Pharma plc, Porton Down, Wiltshire

Human NAD(P)H quinone oxidoreductase 2 (NQO2) is an enzyme that is specifically elevated in most tumours and now being exploited in new ENACT-based therapies. Using a redox cycling reaction, FAD+ present in the active site transfers hydrogen atoms from a co-substrate (cofactor) to a substrate through a sequential 'ping-pong'-type mechanism. This process can be used to reduce a prodrug substrate to a highly toxic alkylating drug species inside a tumour – e.g. CB1954 is an excellent prodrug substrate. NQO2 is unique in that it uses a non-biogenic co-substrate (NRH) of bacterial origin.

Experimental data with synthetic nicotinamide-based NRH replacements show that simple derivatives are superior co-substrates ("cofactors") for NQO2 in the first half of its redox cycle (oxidized→reduced enzyme form). This finding and the 'ping-pong' effect can be rationalized in terms of molecular interactions; the use of NRH as co-substrate, rather than the NAD(H) or NADP(H) used by NQO1, is also explained. Correlation between activity and molecular character has been established using QM/MM methods, and this linkage can predict further NRH replacements. These findings have also been used to predict activity in prodrug/drug substrate families in the second reducing half of the NQO2 redox cycle.

For the first time, the exacting requirements for substrate (and co-substrate) activity by NQO2 can now be understood.

### P32

MANIPULATION OF P450 GENE EXPRESSION IN TUMOURS; A NOVEL APPROACH FOR TARGETED ACTIVATION OF BIOREDUCTIVE PRODRUGS

Robson T, Yakkundi A, McCarthy HO, McErlane V, Hughes CM, Hirst DG, *McKeown SR. Radiation Science group, University of Ulster, Jordanstown, N. Ireland. BT37 OQB

We are developing a gene-directed enzyme prodrug therapy (GDEPT) strategy to enhance metabolism of a novel bioreductive drug, AQ4N. Bioreductive drugs are activated in hypoxic cells allowing effective targeting of hypoxic, radioresistant tumours. We aim to achieve additional selectivity by using an X-ray inducible promoter linked to our therapeutic gene (cytochrome P450s). This should enhance drug metabolism only in the radiation field and spare normal tissues. We have identified several human cytochrome P450s which are important for AQ4N prodrug activation, these include CYP3A4, 1A1 and 2B6. RIF1 murine tumour cells transfected with cDNA from any one of these CYPs displayed increased DNA damage and clonogenic cell kill following treatment with AQ4N under hypoxia compared to controls. When tumours are transfected *in vivo* we have shown that a single CYP3A4 injection using a simple non-optimized approach can increase metabolism of AQ4N, and when used in combination with radiation 3 out of 9 tumours are locally controlled for > 60 days. This implies that the bioreduction of AQ4N by CYPs in this tumour system is sub-optimal and this strategy could therefore be very promising for clinical use where CYP levels are known to be variable. We are also developing CYPs linked to the radiation inducible promoter WAF-1 to allow for selective activation *in vivo*.

*This work is funded by Cancer Research UK. In vivo studies are conducted under the Animals (Scientific Procedures) Act 1986.*

© 2003 Cancer Research UK

### P33

**ABSTRACT WITHDRAWN**

### P34

DEVELOPMENT AND USE OF AN *IN-VIVO* PHENOTYPING TEST FOR CYTOCHROME P450 3A4 IN CANCER PATIENTS.

*Simon J. Brown, Clive Y. Ward, Roland C. Wolf, Elaine M Rankin. Biomedical Research Centre, University of Dundee.

The pharmacokinetics of most cytotoxic drugs show marked inter-patient variability. This may lead to unpredictability in the efficacy of the treatment or the degree of toxicity experienced by patients. Cytotoxic drug dosages are often based on an estimation of the patient's body surface area. This is now thought to be inaccurate as it is not related to physiological measures relevant to drug metabolism and disposition, such as hepatic or renal function. Oxidative metabolism of drugs such as taxanes and vinca alkaloids by the cytochrome P450 (CYP) isoform 3A4, which shows marked variability, may explain some of the variation in pharmacokinetics. Assessment of CYP3A4 enzyme may give a more accurate way of determining the dose of cytotoxic drugs for individual patients. To determine an individual's CYP3A4 activity we have carried out an *in-vivo* phenotyping test, where metabolism of a carefully selected probe drug is used to estimate the activity of the enzyme involved in its metabolism. For CYP3A4 we have used a small dose of Midazolam (a benzodiazepine drug commonly used in conscious sedation) which is predominantly metabolised by CYP3A4. As this method has previously been used in healthy volunteers, we have carried out a pilot study to see if it is safe and applicable to cancer patients, who may have liver disfunction and may be taking other medications which can induce or inhibit CYP3A4 activity. We will extend this study to correlate *in-vivo* phenotyping data for CYP3A4 (using Midazolam and a limited sampling strategy) with drug handling (measured by pharmacokinetic sampling), drug efficacy (assessed by standard RECIST criteria), and drug toxicity (assessed by Common Toxicity Criteria) in patients receiving drugs which are metabolised by CYP3A4.

### P35

CYTOCHROME P450 CYP1B1 VERSUS GLUT 1 PROTEIN EXPRESSION DURING THE DEVELOPMENT OF HEAD AND NECK SQUAMOUS CELL CARCINOMAS (HNSCCs).

Mhairi L Greer[1*], Francis M Daley[1], Paul Barber[1], Graeme I Murray[2], Laurence H Patterson[3], Steven A Everett[1], [1]Gray Cancer Institute, Mount Vernon Hospital, Middx HA6 2JR, [2]University of Aberdeen, [3]The School of Pharmacy, London

The trans-membrane glucose transporter protein (GLUT 1) is detectable in pre-malignant squamous epithelia and squamous cell carcinomas of the head and neck. The aim of this study was to compare the tissue distribution of CYP1B1 and GLUT 1 to ascertain their relative importance as diagnostic molecular markers. HNSCC sections were semi-quantitatively assessed using monoclonal CYP1B1 and polyclonal GLUT 1 antibodies and immunohistochemistry/light microscopy. Membrane-bound GLUT 1 expression in the carcinoma tissue was localised mainly in the tumour periphery but absent from the centre of well-differentiated tumour islands, in contrast CYP1B1 was expressed throughout. CYP1B1 and GLUT 1 both exhibited intra-patient heterogeneity. Protein expression of the two antigens in pre-cancerous tissue (n=17) showed that CYP1B1 was a better bio-marker, as staining was stronger and detectable throughout the tissue (76% CYP1B1 vs. 24% GLUT 1). Membrane bound GLUT 1 protein was strong only at the tip of the rete processes.

| | Immunoreactivity in hyperplasia (%) | | | |
|---|---|---|---|---|
| Marker | Negative | Weak | Medium | Strong |
| CYP1B1 (n=47) | 0 | 40 | 36 | 24 |
| GLUT1 (n=17) | 6 | 29 | 24 | 41 |

The results indicate that CYP1B1 is a superior biomarker for the detection of pre-malignant lesions in HNSCC. The over-expression of CYP1B1 during HNSCC development may prove a useful therapeutic target for CYP1B1-activated prodrugs.

*This work is supported by Cancer Research UK and GLCRT.*

### P36

DMU135: A CYP1B1 ACTIVATED TYROSINE KINASE INHIBITOR PRODRUG WITH TUMOUR SELECTIVE ACTIVITY.

P.C. Butler*, K.C. Ruparelia, T. Ijaz, H.L. Tan, N.E. Wilsher, P.J. Perry, M.D. Burke, and G.A. Potter, Cancer Drug Discovery Group, School of Pharmacy, De Montfort University, Leicester UK LE1 9BH

DMU-135 is a new anticancer prodrug that is activated by the tumour selective catalytic activity of the cytochrome P450 enzyme CYP1B1[1]. Upon oxidative metabolism by CYP1B1 the low-toxicity prodrug DMU-135 is bioactivated intra-tumourally to a potent tyrosine kinase inhibitor. The active metabolite DMU-117 is a potent broad spectrum TK inhibitor that is able to shutdown >30 % of total cellular tyrosine kinase activity. In normal cells DMU-135 typically showed low toxicity (e.g. in MCF-10A cells IC50 = 2 uM). However a marked increase in cytotoxicity was observed in cells expressing the CYP1B1 enzyme. Specifically, in MDA-468 tumour cells the prodrug had very potent cytotoxic activity (IC50 = 0.006 uM). In CYP1 inducible tumour cells, cytotoxicity increased dramatically upon enzyme induction, and was abrogated using enzyme inhibitors. Furthermore, the prodrug had very low toxicity to primary cultured normal human cells (IC50 = 30 uM), hence the tumour selectivity factor is 5000-fold. These results suggest that DMU-135 is a tumour selective prodrug useful for the treatment of CYP1B1 expressing tumours. Moreover, because the active metabolite inhibits multiple tyrosine kinase pathways, resistance due to second messenger redundancy is unlikely.

1. G.A. Potter and P.C. Butler, 3,4-Methylenedioxy Chalcones (DMU-135), *British Patent Appl. GB 0123777*, 2001.

**P37**

CYP1B1 PROTEIN EXPRESSION IN PROSTATE CARCINOMA,
DM Carnell[1,2*], FM Daley[1], RE Smith[3], P Hoskin[2], GI Murray[4], & SA Everett[1], [1]Gray Cancer Inst, [2]Marie Curie Res Wing, [3]Dept Pathol, Mount Vernon Hosp, Northwood, Middlx [1]HA6 2JR or [2,3]HA6 2RN. [4]Dept Pathol, Univ Aberdeen, Aberdeen AB25 2ZD.

Previous cDNA microarray and RT-PCR studies have demonstrated the expression of cytochrome P450 CYP1B1 in malignant prostate and benign prostatic hyperplasia. Normal tissues including the liver can express the CYP1B1 gene without expressing the protein. The aim of this study was to compare CYP1B1 protein expression in prostate carcinoma with bladder carcinoma which over-expresses CYP1B1. Protein expression was detected immunohistochemically using a monoclonal antibody specific for CYP1B1. Radical prostatectomy specimens (n = 14) of moderate Gleason grade 3+3 and 3+4 were compared retrospectively with biopsies taken from patients with bladder carcinoma (n = 20). The intensity of immunoreactivity was assessed semi-quantitatively and graded as strong, moderate, weak, or negative against a positive control. The majority of the prostate carcinoma tissue was positive for CYP1B1 although both inter- and intra-patient variability was evident; 2 tumours (14 %) were negative, 1 tumour (7 %) was weak, 7 (50 %) tumours were moderate and, 4 tumours (28 %) exhibited strong CYP1B1 immunoreactivity. In both prostate and bladder carcinoma, CYP1B1 protein expression was localised in the cytoplasm of the tumour cells and was undetectable in the associated normal stromal tissue. Bladder carcinoma exhibited greater CYP1B1 protein expression with moderate (45 %) to strong (55 %) immunoreactivity. CYP1B1 protein expression was also detected in benign hyperplastic prostate epithelium present in 13 (93 %) samples. In conclusion, human prostate carcinoma expresses the CYP1B1 protein with high frequency (n = 14, 86%) but lower immunoreactivity than bladder carcinoma. Future work will measure CYP1B1 functional activity in prostate carcinoma. *This work is supported by Cancer Research UK*

**P38**

INHIBITION OF THE MDM2-P53 INTERACTION AS A POTENTIAL TARGET IN CANCER THERAPY

Gillian Farnie, Shafiq Ahmed* and John Lunec
Northern Institute for Cancer Research, University of Newcastle, Medical School, Framlington Place, Newcastle NE2 4HH, United Kingdom. E-mail: S.U.Ahmed@ncl.ac.uk

MDM2 gene amplification and/or overexpression in a range of tumours causes inactivation of the p53 tumour suppressor protein. Inhibitors of MDM2-p53 interaction are therefore therapeutic candidates for activation of the p53 pathway in tumours.

AP-B, an isomer of a previously published synthetic peptide (AP, Chène *et al* 2000, *J. Mol. Biol.* **299**. 245-253), structurally based on the p53 binding domain for MDM2, was tested on wild type p53 cell lines with amplified and overexpressed MDM2 (SJSA and LS) or normal levels of MDM2 (HCT 116 and A2780). A luciferase based gene reporter assay was used to measure transcriptional activity of p53 and Western blotting to determine protein levels of p53, MDM2 and $p21^{WAF1}$ at 4, 9 and 24 hours. Cell line growth and cell cycle distribution was evaluated using Sulforhodamine B (SRB) assays and FACS analysis.

The AP-B peptide (100μM) significantly increases p53 activity in overamplified MDM2 cell lines (9 fold and 5 fold increases in SJSA and LS cell line respectively). This correlated with Western blot data, which showed an increase in the levels of p53, MDM2 and $p21^{WAF1}$. FACS analysis showed a significant increase in G1 arrest with the SJSA cell line, in concordance with $p21^{WAF1}$ levels. SRB data on control cell lines SaoS-2, (p53 null) and HCT 116 N7 (p53 degraded) showed no cell growth inhibition at 72 hours, indicating that growth arrest is dependent on wild type p53 activation. These findings confirm and validate the MDM2-p53 interaction as a potential target in cancer therapy, particularly for MDM2 overexpressing tumour.

**P39**

*IN VITRO* STUDY OF THE P53 PATHWAY IN PERIPHERAL BLOOD MONONUCLEAR CELLS IN RESPONSE TO DOXORUBICIN TREATMENT

*Maged S. Nashed, George A. Thomson, Fiona E.M. Paulin, Julie Wardrop, Elaine M Rankin, David P Lane. Biomedical Research Centre, Department of Surgery and Molecular Oncology, University of Dundee.

**Introduction and Aims** p53 is a tumour suppressor gene which is mutated in more than 50% of all sporadic cancers. The relationship between p53 status, response to treatment and the degree of side effects remains controversial. The aims of this study were to examine the pattern of changes in the key genes involved in the p53 pathway in normal tissues upon *in vitro* exposure to doxorubicin by means of western blotting (WB) and to examine the correspondence between immunohistochemistry (IHC) and WB findings in relation to the different levels of p53 protein. **Materials and Methods** Peripheral blood mononuclear cells (PBMNC) were used as a surrogate marker of normal tissue response to chemotherapy. PBMNC were isolated from normal subjects by density gradient centrifugation. Parallel experiments were done using resting and phytohemagglutinin activated PBMNC where cells were treated *in vitro* with different doses of doxorubicin for different durations designed to simulate various *in vivo* situations. p53, p21 and caspase 3 monoclonal antibodies were used for WB. IHC was done from formalin fixed paraffin embedded cell blocks of PBMNC. **Results** The overall pattern of p53 induction was similar among different individuals. However, the degree of its expression varied in relation to both the dose and the length of exposure to doxorubicin. p21 induction (as a marker of p53 transcriptional activity) followed the same pattern as p53 in activated PBMNC. Early activation of caspase 3 (as a marker of effector stage of the apoptotic pathway) in activated cells was also seen. The degree of p53 induction detected by WB corresponded to that detected by IHC and the degree of caspase 3 and p53 induction detected by WB correlated with PBMNC morphological changes. **Conclusions** Different degrees of induction of the key genes involved in the p53 pathway can be studied in PBMNC. This study opens an interesting field of *in vivo* experiments using IHC on PBMNC to identify molecular markers, which could potentially be used to predict chemotherapy toxicity to normal tissues.

**P40**

BCL-2 CONSTITUTIVELY SUPPRESSES P53-DEPENDENT APOPTOSIS IN COLORECTAL CANCER CELLS

Ming Jiang and Jo Milner* Yorkshire Cancer Research P53 Laboratory, Department of Biology,University of York, YORK YO10 5DD, UK

To dissect apoptotic genes governing the survival of colorectal carcinoma cells we employed RNAi to silence Bcl-2 and Bcl-$x_L$ in isogenic clones of p53+/+ and p53-/- cells, and of Bax+/- and Bax-/- cells. We identify a novel pro-apoptotic function of p53 that does not require activation by genotoxic agents and that appears to be constitutively suppressed by Bcl-2. Silencing of Bcl-2 induced massive p53-dependent apoptosis. The "Bcl-2/p53 axis" requires Bax and caspase 2 as essential apoptotic mediators. This newly discovered Bcl-2/p53 functional interface represents a key regulator of apoptosis which can be activated by targeting Bcl-2 in colorectal carcinoma cells.

© 2003 Cancer Research UK

**P41**

$P16^{INK4a}$ and $P15^{INK4b}$ ALTERATIONS IN VULVAL AND ENDOMETRIAL CANCER

Yuzhu Cheng* and Anthony D Blackett
Sheffield University Department of Obstetrics & Gynaecology, The Jessop Wing, Sheffield S10 2SF

$P16^{INK4a}$ and $p15^{INK4b}$ are tumour suppressor genes located on chromosome 9p21. These two genes regulate the cell cycle pathway by inhibiting the cyclin D/CDK4/6 mediated phosphorylation of pRb. In human neoplasms, the $p16^{INK4a}$ and $p15^{INK4b}$ genes may be silenced in the following ways: homozygous deletion, promoter methylation and point mutation. The 9p21 gene cluster has been implicated in a variety of tumour types and derived cell lines. Although $p16^{INK4a}$ and $p15^{INK4b}$ genes are known to play a role in many malignancies, their significance in vulval cancer (VC) and endometrial cancer (EC) was uncertain, and their physiological role remained unclear.

To clarify the mode of inactivation of these genes in primary cancers, we performed multiple molecular analyses on samples of 34VCs and 20 ECs. A comparative multiple PCR assay was used for the detection of homozygous deletions. The methylation status of samples was assessed using sodium bisulphate modification prior to PCR amplification. Protein expression was detected by western blotting and immunohistochemistry.

The findings indicated that in vulval cancer inactivation of $p16^{INK4a}$ is predominantly caused by promoter methylation, whereas in activation of $p15^{INK4b}$ is frequently caused byaberrant promoter methylation along with some homozygous deletion. Promoter methylation of $p16^{INK4a}$ but not $p15^{INK4b}$ occurred in endometrial carcinoma. Our results show that the mode of alteration at 9p21 was not uniform but was selective, dependent upon histological type. The two genes are inactivated by different mechanisms during primary VC and EC development.

**P42**

EXAMINATION OF THE RESPONSE OF BREAST CANCER CELLS, ALTERED IN P53 STATUS, TO DOXORUBICIN

Fiona E. M. Paulin*, Julie Wardrop, Clive Ward, David P. Lane, Elaine M. Rankin, Alastair M. Thompson & C. Roland Wolf.
University of Dundee, Biomedical Research Centre, Level 5, Ninewells Hospital and Medical School, Dundee, DD1 9SY.

Doxorubicin is widely used in the treatment of many cancers, however the clinical response and side effects differ considerably between patients. In part, the genotypic and phenotypic characteristics of a tumour may explain this phenomenon, but differences in patient drug metabolism may also be important. One key gene involved inthe cytotoxic response is the tumour suppressor gene p53. It is frequently mutated in cancers but the relationship between p53 mutational status and response to chemotherapy remains unclear.

To address this question in more detail we created two dominant negative (DDp53) breast cancer cell lines. The parental cell line, MCF7(p53 wildtype), and the DDp53 cell lines (p53 non-functional) were then treated with different doses of doxorubicin *in vitro*. Drug levels were quantified over time by HPLC-MS/MS and cytotoxicity assays were performed. In addition, gene expression was analysed by microarray technologies and quantitative PCR techniques. Protein levels of key factors were also examined by western blotting.

The DDp53 cell lines showed a greater resistance to doxorubicin and displayed a different basal gene expression pattern compared to the parental cell line. In addition the gene expression pattern induced was dependent on the dose of doxorubicin given. These results clearly demonstrate the important role of p53 in response to doxorubicin and imply that drug doses delivered to a tumour may be critical in determining the tumour response.

**P43**

COMPARISON OF THE EFFICACY OF ANTIBODY DERIVATIVES CARRYING DIFFERENT NUMBER AND GEOMETRY OF Fc IN GUINEA PIG LEUKAEMIA THERAPY.

*Kwok S. Kan, Andrew M. Smith, George T. Stevenson. Institute for Health Policy & Research, London Metropolitan University, Holloway, London N7 8DB.

**Aim** We studied two different factors which might affect the efficacy of antibody derivatives in leukaemic guinea pigs. We investigated the therapeutic effects of (i) increasing the number of Fc( conjugated to antibody derivatives, (ii) conjugating two Fc( either randomly to $Fab_2$ or specifically to the hinge of $Fab_2$.

**Methods** All human Fc( were used. $Fab_2Fc_1$, $Fab_2Fc_2$, and $Fab_2Fc_3$ were synthesised from Fab( of 5G10 murine monoclonal antibody. A thiolating agent N-succinimidyl-S-acetylthioacetate (SATA) was used to randomly introduce sulfhydryl (SH) groups to murine RJD $Fab_2$ followed by crosslinking between Fc( and Fab( via phenylenedimaleimide (OPDM) to yield $Fab_2$ $Fc_2$. Another $Fab_2Fc_2$ was produced by specifically crosslinking two Fc( to the SH groups in the hinge of RJD $Fab_2$ via OPDM. Strain 2 guinea pigs were inoculated i.p. with 5 x $10^4$ $L_2C$ leukaemic cells. After 24 hours, 4 mg of each antibody derivative was injected intravenously.

**Results and conclusion** Firstly, $Fab_2Fc_2$ produced a higher therapeutic response than $Fab_2Fc_1$ or the control animals. However, $Fab_2Fc_3$ performed no better than $Fab_2Fc_2$. Therefore, a higher number of Fc( than 2 within an antibody derivative may lead to steric hindrance which hampers antibody binding and recruitment of effectors. Secondly, SATA-linked $Fab_2Fc_2$ showed a better therapeutic response than hinge-linked $Fab_2Fc_2$. However, SATA-linked $Fab_2Fc_2$ produced greater breakdown products *in vivo* than hinge-linked $Fab_2Fc_2$. Increased *in vivo* breakdown of SATA-linked $Fab_2Fc_2$ may generate more univalent $FabFc_2$ remnants which would outperform hinge-linked $Fab_2Fc_2$ due to antigenic modulation

**P44**

IDENTIFICATION AND REPLICATION OF REACTIVE T-CELLS SPECIFIC AGAINST RENAL CELL CARCINOMA

Ciaran B Lynch,* Paul A Moss, Nicholas D James.
CR UK Institute of Cancer Studies, University of Birmingham. Edgbaston. Birmingham B15 2TH.

**Aims:** This project attempts to identify cytotoxic T-lymphocytes found within the tumour substance which are reactive against Renal Cell Carcinoma (RCC). RCC is a highly immunogenic tumour and has a large number of Tumour Infiltrating Lymphocytes (TILs).

**Procedure:** Patient volunteers undergoing nephrectomy for RCC are identified, consent obtained and a sample of tumour and matched normal kidney obtained. Ethical approval for this has been granted. Primary tissue is mechanically disaggregated and TILs are extracted from the tumour tissue by density gradient centrifugation. Standard methods of tissue culture are then used. T-cells, when stimulated by antigen, release cytokines and recent technological advances allow these cells to be identified and isolated. Standard methods of T-cell cloning are then used to expand and clone these tumour-specific cytotoxic T-lymphocytes.

**Major findings**: It has been possible to identify and grow tumour-specific T-cells *in vitro.* These cells are rare, amounting to approximately 1 in 100 000 TILs. IL-2 expands this population further, but at the expense of tumour specificity.

**Conclusions:** The above results are promising in the development of anti-tumour T-cells. Work is ongoing in T-cell cloning and measurement of anti-tumour activity against autologous tumour targets. These cells, if expanded sufficiently, would offer therapeutic opportunities in a Clinical Trial setting for T-cell adoptive immunotherapy.

© 2003 Cancer Research UK

P45

CD20 INDUCED APOPTOSIS IS AN ANTIBODY DEPENDENT PHENOMENON WHICH DOES NOT REQUIRE REDISTRIBUTION INTO TX-100 INSOLUBLE MEMBRANE RAFTS

H T Claude Chan, David Hughes, Ruth R French, Alison L Tutt, Claire Walshe, Martin J Glennie, Mark S Cragg*

*Tenovus Research Laboratory, Cancer Sciences Division, School of Medicine, General Hospital, Tremona Road, Southampton, SO16 6YD United Kingdom

Rituximab (Ritux) is in prevalent use in the clinic for the treatment of neoplasia and is currently being evaluated for use in autoimmune disorders. Although depletion of CD20 expressing cells occurs in both cases, the mechanism remains uncertain. Apoptosis may be one means through which depletion is achieved. Here, CD20 apoptosis was investigated with a panel of anti-CD20 mAb in a wide range of cell-lines. Although CD20 apoptosis was dependent on the target cells a hierarchy of mAb activity was apparent, with the B1 mAb generally the most potent. Interestingly, apoptosis was dependent upon the nature of binding employed by the different anti-CD20 mAb and correlated with the extent of homotypic cell adhesion caused. However, using previously characterised anti-CD20 mAb which vary in the extent to which they translocate CD20 to membrane rafts and a range of mutated CD20 molecules with diminished ability to redistribute to membrane rafts, we were able to determine that CD20 apoptosis is independent of translocation to TX-100 insoluble rafts. Further investigation of CD20 mutant molecules revealed that the apoptosis inducing function of CD20 does not appear to reside in the C terminus. We conclude that CD20 induced cell death is an antibody dependent process which is linked to the mode of mAb binding and does not require redistribution into TX-100 insoluble membrane rafts.

P46

MANIPULATION OF MACROPHAGE POPULATIONS CAN ENHANCE LOW DOSE RADIATION PLUS ANTI-CD40 MAB THERAPY. Jamie Honeychurch*, Martin J. Glennie, Peter W.M. Johnson, Timothy M. Illidge. Cancer Sciences Division, Southampton General Hospital, Southampton University, SO16 6YD, UK

We have demonstrated that combining total body irradiation (TBI) and anti-CD40 monoclonal antibody (mAb) can result in long-term protection in murine B-cell lymphoma and this protection is mediated by CD8+ T-cells (CTL). Therapeutic efficacy directly correlates with radiation dose, and we believe that this reflects a threshold of induced tumour cell death needed to provide critical amounts of antigen for uptake by “licensed” antigen presenting cells (APC) capable of priming CTL.

Which APC are most effective at processing and presenting antigen from dying cells is controversial. Evidence suggests that whilst macrophages (Mϕ) are the primary scavenging cell, they are unable to cross-present efficiently. Thus, we investigated if manipulation of Mϕ populations could enhance radiation therapy. Using flow-cytometry and fluorescent microscopy we have shown that tumour cells are rapidly engulfed by Mϕ following TBI, and that the degree of engulfment directly correlates with both the dose of radiation delivered and the amount of apoptosis observed. Treatment of mice with silica to selectively deplete Mϕ prior to TBI results in reduced tumour cell clearance in secondary lymphoid organs. Conversely, treatment with thioglycollate to increase Mϕ numbers results in a more rapid, and extensive clearance of dying tumour. Interestingly, these differences in antigen longevity and availability in the major sites of tumour growth post-irradiation are reflected in therapeutic efficacy. The degree of protection observed following low-dose radiation and anti-CD40 mAb therapy was significantly increased in animals depleted of Mϕ . This work was supported by CR UK.

P47

ANTI-CD40 MONOCLONAL ANTIBODY THERAPY: TREATMENT TOXICITY AND IMPLICATIONS FOR CANCER IMMUNOTHERAPY

Thomas Geldart*, Melanie Harvey, Norman Carr, Martin Glennie Peter Johnson, Cancer Research UK Oncology Unit, Southampton General Hospital, Southampton, SO16 6YD.

**Background** The critical importance of CD40 signalling in the development of CD8+ cytotoxic T cell responses and the expression of CD40 on a broad range of malignancies make CD40 an attractive target for immunotherapy. Published data has demonstrated that anti-CD40 therapy provides significant therapeutic effects and protects against tumour re-challenge in a syngeneic mouse model of malignancy[1].

**Aims** To establish the toxicity profile of anti-CD40 monoclonal antibody (mAb) treatment in an animal model.

**Methods** Rat anti-mouse CD40 mAb (3/23) was administered at a variety of dose levels to cohorts of BALB/c and C57BLK/6 mice. Biochemical, haematological and histopathological data was gathered over a period of three months.

**Results** There was no treatment related mortality. Anti-CD40 mAb therapy caused a dose dependent but fully reversible lympho-granulomatous hepatitis. Mild, reversible tubulo-interstitial lymphocytic nephritis and dose related splenomegaly were also noted.

**Conclusion** Anti-CD40 mAb treatment in mice is limited by dose dependent but reversible hepatic toxicity, a result in keeping with the published dose limiting toxicity of recombinant human CD40 ligand in humans[2]. These results have implications for the treatment of human cancers with anti-CD40 mAb immunotherapy.

**References**

1. French RR et al. 1999. Nature Medicine 5:548-53.
2. Vonderheide RH et al. 2001. J Clin Oncology 19:3280-7.

P48

DESIGNER T CELLS FOR THE IMMUNOTHERAPY OF CANCER.

David E. Gilham*, Ryan D Guest, Eleanor J Cheadle, Chris Hughes, Natallia Kirillova, Allison O’Neill, Joely Irlam and Robert E Hawkins.
Cancer Research UK Department of Medical Oncology, Paterson Institute, Christie Hospital NHS Trust, Manchester, M20 4BX.

T cells can be an effective treatment for malignant disease. However, tumours can avoid immune recognition through a variety of mechanisms including the down-regulation of important T cell recognition molecules. Chimeric Immune Receptors (CIR’s) consisting of antibody fragments (scFv) fused to the CD3ζ chain effectively re-direct T cell activity to protein antigens directly thereby circumventing tumour avoidance of T cell recognition.

T cells from healthy donors transduced with recombinant retroviruses encoding an anti-carcinoembryonic antigen (CEA) scFv.CD3ζ CIR effectively respond to and lyse antigen-specific target cell lines *in vitro*. Protection against tumour cell growth *in vivo* was observed when the gastric carcinoma cell line MKN45K was co-administered sub-cutaneously in NOD/SCID mice with CIR expressing human T cells (3/5 animals surviving at 78 days) while mock treated cells offered no additional protection over tumour only controls (0/5 animals surviving at 78 days). Importantly, a dose-dependent effect was observed when higher levels of transduced T cells present within the population generated correspondingly increase delays in tumour growth. Further experiments have indicated that the methods of pre-culture, and, in particular, the concentration of cytokines used to grow the T cells, can have a major influence upon the activity of the T cells

This work demonstrates that gene-modified T cells can challenge the growth of CEA expressing tumour cells *in vitro* and *in vivo*.

 © 2003 Cancer Research UK

**P49**

DESTRUCTION OF CD19+ LYMPHOMAS BY T CELLS EXPRESSING A CHIMERIC RECEPTOR

Eleanor J Cheadle*, Anne Armstrong, John Radford, David E Gilham, Robert E Hawkins.

Department of Medical Oncology, Paterson Institute for Cancer Research, Christie Hospital, Manchester, M20 4BX.

Chimeric T cell receptor technology enables the coupling of an antibody scFv to TCR signalling molecules such as CD3ζ, thus enabling the targeting of cytotoxic T cells to tumour without the need for MHC restriction.

CD19 is a signal transduction molecule expressed on the surface of all mature B cells and lymphomas but lost upon maturation to plasma cell phenotype. Chimeric receptors were constructed that expressed either an anti CD19 scFv coupled to the transmembrane and cytosolic domain of the CD3ζ molecule (CD19.CD3ζ), or an anti CD19 scFv coupled to the CD3ζ molecule via the human IgG1 CH2CH3 Fc spacer region (CD19hFc.CD3ζ). Constructs were cloned into retroviral vectors, which enabled efficient transduction of proliferating T cells.

Retroviral transduction of healthy donor T cells with chimeric receptor genes expressing CD19.CD3ζ or CD19hFc.CD3ζ, receptors led to comparable specific killing of a CD19+ Burkitt lymphoma cell line and IFNγ release upon culture with the Raji cell line. However, IFNγ release from CD19hFc.CD3ζ transduced T cells was significantly reduced compared to that from CD19.CD3ζ transduced T cells. Importantly, transduced patient T cells specifically killed autologous tumour cells derived from lymphoma biopsy samples.

The efficacy of T cells expressing a CD19.CD3ζ chimeric receptor is currently being evaluated in a murine model system.

**P50**

THE EFFECT OF ADEPT, AS A SINGLE OR COMBINED THERAPY, IN TWO COLORECTAL XENOGRAFTS.

Surinder K Sharma*, R Barbara Pedley, Helen L Irwin, Ethaar El-Emir, Geoffrey M Boxer, Robert W Boden, Kerry A Chester & Richard H J Begent,

CR UK Targeting & Imaging Group, Dept. of Oncology, RF & UCMS, UCL,Royal Free Campus, Rowland Hill Street, London NW3 2PF, UK.

The out come of antibody-directed therapies is related to the morphology and pathophysiology of the tumor.

Antibody directed enzyme prodrug therapy (ADEPT) has therefore been studied in two CEA-producing human colorectal tumour xenografts, differing in both these aspects. The LS174T model is a moderate to poorly differentiated adenocarcinoma. The SW1222 is organized into well-defined glandular structures around a central lumen. Biodistribution studies of a recombinant fusion protein (MFE-CP) comprising an anti-CEA single chain Fv (MFE-23) fused to the bacterial enzyme carboxypeptidase G2 (CPG2) showed localization of CPG2 activity in tumours and clearance from normal tissues, within 6 hours after injection. A single cycle of MFE-CP in combination with prodrug resulted in reproducible growth delay for both LS174T and SW1222 colorectal tumor xenografts without systemic toxicity, but was more effective in the SW1222 (10v15 days growth delay). Repeated ADEPT cycles gave significantly extended growth delay in LS174T but produced regressions in the SW1222model, with minimal toxicity. The microscopic effects of ADEPT on tumour biology have also been studied using quantitative digital microscopy.

Agents which enhance the effect of ADEPT when used in combination are being investigated, and will be discussed.

*In-vivo work in compliance with UKCCCR guidelines.*

Supported bythe Cancer Research Campaign, AstraZeneca and the Association for International Cancer Research.

**P51**

ISOLATION OF TUMOUR CELLS FROM PERIPHERAL BLOOD FOR POTENTIAL ASSESSMENT OF RESPONSE TO ANTIBODY DIRECTED ENZYME PRODRUG THERAPY (ADEPT). Helen L Irwin[1*], Frances Corcoran[1], Geoffrey M Boxer[1], R. Barbara Pedley[1], Jason Dearling[1], Janet M Hartley[2], Richard H J Begent[1] and Surinder K Sharma[1].

[1]CRUK Targeting and Imaging Group, [2]Molecular and Cellular Pharmacology Group, Dept.of Oncology, RF & UCMS, UCL, Royal Free Campus, Rowland Hill Street, London NW3 2PF.

The detection of circulating tumour cells from peripheral blood is of clinical importance in terms of monitoring disease progression, response to therapy and determining the mechanisms by which treatments exert their effect.

Two techniques (StemSep™ and RosetteSep™) were investigated whereby the haemopoietic cells are removed, leaving the enriched tumour cell population unaltered. Both methods were applied to separate spiked CEA-positive human colorectal tumour cells, SW1222, from whole blood obtained from healthy donors after written consent. RosetteSep™ was determined to be the better and more sensitive of the two techniques and was investigated further. Immunohistochemistry showed isolated tumour cells to be CEA positive, Cytokeratin positive and LCA (leucocyte common antigen) negative thus demonstrating the specificity of the separation technique. SW1222 tumour cells were then treated *in vitro* with the components of ADEPT, spiked into healthy whole blood and separated out using RosetteSep™. COMET assays were performed on tumour cell samples to determine the extent of DNA cross-linking caused by the ADEPT system.

The results indicated that the negative cell separation procedure could isolate ADEPT treated cells from peripheral blood. This implied that RosetteSep™ has potential application in isolating circulating tumour cells in peripheral blood of cancer patients. These cells can then be characterised further to determine the level of cellular damage caused by cancer therapy and yield data to inform design of future therapeutic strategies.

**P52**

IDENTIFICATION OF CTL EPITOPES FROM TUMOUR ANTIGENS IN HUMAN LUNG CANCER

Angela J Darby*, Peter W M Johnson and Tim J Elliott

Cancer Sciences Division, University of Southampton, UK

**Aims:** To identify CTL epitopes from novel tumour antigens in human lung cancer to lead to new targets for clinical trials of immunotherapy.

**Background:** Lung cancer is the commonest cause of cancer death in the United Kingdom and, despite advances in conventional chemotherapy and radiotherapy, only 5% of patients are alive at 5 years. CTL-based immunotherapy is based on the observation that cytotoxic T lymphocytes (CTL) can make an effective response to specific autologous tumour antigens.

**Methods:** Web-based algorithms were used to identify nonamer peptides with potential to act as CTL epitopes for nine tumour antigens with expression in lung cancer. Binding to HLA-A*0201 was measured with a T2 stability assay and experimental results for each antigen were ranked and compared to predictive results from the algorithms. Peptides shown to bind to HLA-A*0201 were used to prime CTL responses in a human ex-vivo system.

**Results:** Approximately 1/3 of peptides predicted to bind to HLA-A*0201 demonstrated no binding in a T2-stabilisation assay. Two of 5 peptides used in the ex-vivo system produced CTL that released peptide-specific interferon-γ in an ELISPOT assay.

**Conclusions:** While predictive algorithms can guide epitope identification, experimental validation is required. We are generating peptide specific CTL to test cytotoxicity using tumour antigen-bearing cancer cell lines.

### P53

### PHASE I/II STUDY OF FRACTIONATED RADIOIMMUNOTHERAPY (RIT) IN RELAPSED LOW GRADE NON HODGKIN'S LYMPHOMA (NHL).

*Mike Bayne*, Maureen Zivanovic, Val Lewington, Yong Du, Christine Harrison, Liz Hodges, Peter Johnson, Tim Illidge.*
CR UK Wessex Medical Oncology Unit, University of Southampton, Southampton General Hospital, SO16 6YD, UK.

Despite highly promising results with RIT in "low grade" NHL there remains uncertainty as to the optimal treatment approach. Currently a single myeloablative dose of radiolabelled anti-CD20 monoclonal antibody (mAb) is given. Both commercially developed radioimmunoconjugates, $^{131}$I-Tositumomab (Bexxar) and $^{90}$Y-Ibritumomab tiuxetan (Zevalin) use murine mAbs and the development of human anti-mouse antibody responses may prevent more than one administration. Impressive durable responses from clinical studies using higher myeloablative dose RIT followed by peripheral blood stem cell transplant (PBSCT) suggest that there may be a radiation dose response for RIT. With the development of Rituximab, a chimeric mAb, multiple or fractionationed treatments are possible, which in turn may enable higher cumulative doses of RIT to be delivered without the need for PBSCT. The primary goal of this study is to test the safety and efficacy of fractionated RIT in relapsed CD20 positive NHL in a dose escalation study using two fractionated doses of labelled Rituximab. Eligible patients will receive two fractions of $^{131}$I labelled Rituximab given with an eight weeks interval. Clinical, radiological, molecular response and toxicity data are being prospectively collected. In addition an analysis of serum rituximab levels during the course of the therapy is being performed and a novel technique for measuring the radiation dose delivered during RIT using the cytogenetic analysis of cultured peripheral blood lymphocytes is being developed. We report on the feasibility of this approach as well as toxicity and early response data on the first 6 patients.

### P54

### LONG TERM CLEARANCE OF TUMOUR IN RADIOIMMUNOTHERAPY (RIT) OF B-CELL LYMPHOMA REQUIRES TARGETED RADIATION AND MONOCLONAL ANTIBODY (mAb) INDUCED CELL SURFACE SIGNALLING

Yong Du, Jamie Honeychurch, Peter Johnson, Martin Glennie, Tim Illidge* Cancer Sciences Division, Southampton University School of Medicine, Southampton, UK. SO16 6YD

…RIT has emerged as a new treatment option for the treatment of lymphomas however the mechanisms underlying the tumour responses are poorly understood. We have therefore investigated the relative contributions of mAb effector mechanisms and targeted radiation to the clearance of tumour in vivo using two different murine syngeneic B cell lymphoma models (A31 and $BCL_1$). In this study we initially demonstrate an additive effect of external beam irradiation (EBRT) and anti-Id mAb, which for the $BCL_1$ tumor model was a modest improvement in tumour protection of around 15 days over control animals. However when we used $^{131}$I anti-MHCII mAb to target radiation in combination with unlabelled anti-Id mAb higher tumour doses could be delivered (18Gy per 18.5 MBq $^{131}$I anti-MHCII) and this resulted in 100% prolonged disease free survival in both tumor models at 100 days as well as prolonged survival with $^{131}$I anti-MHC and anti-CD19. Using in vivo tumour tracking and we have demonstrated that this additive cytoreductive effect is dependent on both targeted radiation and signaling mAb. Finally we have shown that the clearance of tumour appears to correlate with the ability of mAb (anti-Id, anti-CD19) to initiate cell surface signalling as measured by intracellular tyrosine phosphorylation on western blot analysis and intracellular calcium influx. In conclusion we have shown that the long term clearance of tumour in vivo critically depends upon the ability of mAb to target radiation to tumour as well provide cell surface signalling to downstream apoptotic pathways.

### P55

### DESIGNER T CELLS: ROLE IN THE TREATMENT OF HEPATIC COLORECTAL METASTASES

Daren. A. Subar*, David. E. Gilham, David. J. Sherlock and Robert. E. Hawkins
Cancer Research UK Department of Medical Oncology, Paterson Institute for Cancer Research, Christie Hospital, M20 4BX

T lymphocytes genetically engineered to target tumour cells are attractive as a potential immunotherapy. Previous work has shown that T cells isolated from patients with advanced colorectal disease can be effectively targeted to kill carcinoembryonic antigen (CEA) expressing cell lines through the expression on the T cell of chimeric receptors composed of an anti-CEA single chain antibody (scFv) fused to the CD3 ζ chain. It is of great importance to determine if these modified T cells maintain specific targeted activity after transduction and expansion in conditions relevant to clinical protocols. To investigate this we used media suitable for clinical practice; (AIM V) with mixed human AB serum in the presence of clinical grade interleukin-2 (IL-2).

T cells isolated from patients with colorectal liver metastases were transduced and expanded in these clinically relevant conditions. Greater than ten fold expansion of T cells was observed during a two week time frame when cultured with 100 IU/mL IL-2. These cells were shown to be functionally active in *in vitro* assay. To improve the overall yield of T cells for clinical use, we are investigating the effects of varying culture conditions upon T cell expansion and function. These experiments will provide information concerning the kinetics of gene modified T cell growth, which has important ramifications upon the transfer of this technology into an effective clinical therapy.

### P56

### DNA-Fc FUSION VACCINE TARGETING THE ENDOTHELIAL ANTIGEN TIE-2

Judith Ramage*, Rachael Metheringham, Robert S Moss,
Ian Spendlove, Robert Rees and Lindy Durrant
CRUK Clinical Oncology, Nottingham University, Nottingham.

Tie-2 stabilised pericyte/endothelial interactions during angiogenesis and is highly expressed on tumour endothelium. A vaccine that targets endothelium over-expressing Tie-2 may result in vessel damage and stimulate an inflammatory cascade resulting in disease regression. We have previously identified an enhanced HLA-A*0201 peptide in Tie-2 which can stimulate CTL responses that recognise T2 cells pulsed with either enhanced or wild type peptide. Incorporation of this enhanced epitope within a Tie-2 DNA construct stimulated poor CTL responses in HLA-A2 transgenic mice. Recent studies have shown that targeting the high affinity Fc receptor results in dendritic cell activation and presentation of both class I and class II epitopes. Fc- vaccines have been used successfully to stimulate both CD8 and CD4 responses to MAGE-3, HBV, CEA and CD55. Human Fcγ1 was therefore incorporated into the Tie-2 DNA vaccine. A dramatic increase in the frequency of γIFN secreting cells that were specific to both native and wild type peptides were demonstrated by ELISPOT in the mice immunized with the Tie-2Fc construct. Moreover these CTL recognise endothelial cells over-expressing Tie-2. This Tie-2 vaccine is currently being screened for toxicity and efficacy in tumour models. The human Fc region is probably providing both foreign T cell help and also allowing receptor mediated uptake and activation of dendritic cells in vivo. To assess the relative contribution of these mechanism of action a Tie-2 mouse Fc construct is currently being evaluated. These studies provide a novel approach to cancer vaccination.

© 2003 Cancer Research UK

**P57**

A MONOCLONAL ANTIBODY DIRECTED AGAINST SCR1-2 OF COMPLEMENT CONTROL PROTEIN, CD55 ENHANCES C3 DEPOSITION AND TUMOUR CELL LYSIS

R G Bradley*, J Morgan, I Spendlove and L G Durrant. CRUK Academic Unit of Clinical Oncology, Nottingham university, Nottingham City Hospital.

CD55 (Decay Accelerating Factor) is a glycophosphatidylinositol (GPI) anchored membrane bound complement control protein expressed on most cells including endothelial cells and leukocytes. CD55 accelerates the decay of both complement activation pathways, specifically C4b2a or C3bBb and corresponding C5 convertases, thus protecting cells from complement mediated bystander attack. Structurally, CD55 contains four Short Consensus Repeat domains, SCR 2-4 possessing decay accelerating activity, while SCR3 mutations have shown to disrupt both alternative and classical complement pathways.

Complement Deposition assays have shown that using the CD55 over-expressing osteosarcoma, 791T, cell line that mAb 791T/36, directed at SCR1-2, produces increased C3b/c deposition compared to BRIC 216 (SCR3 directed). 791T/36 also showed effective DAF blockade when compared to 1C6 and 1H4 monoclonal antibodies (also SCR3 directed). In order to assess the effectiveness SCR1-2 blockade, Chromium$^{51}$ release assays have been performed to determine whether inhibition of CD55 activity leads to tumour cell lysis and how this compares to lysis induced by alternative SCR blockade.

This novel approach for a SCR1-2 targeted monoclonal antibody, blocking CD55 function and generating potential therapeutic effects may be further analysed.

**P58**

INVESTIGATION OF THE HUMAN TELOMERASE RNA GENE CORE PROMOTER IN BASAL TRANSCRIPTION

Jiangqin Zhao*, Alan Bilsland, Stacey F. Hoare and W. Nicol Keith. Cancer Research UK Department of Medical Oncology, University of Glasgow, Cancer Research UK Beatson Laboratories, Garscube Estate, Switchback Road, Bearsden, Glasgow G61 1BD, UK.

The telomerase RNA gene, hTR, is regulated by a variety of transcription factors including NF-Y and Sp1. The hTR proximal promoter contains four Sp1 sites and one CCAAT-box. We have carried out a functional analysis of the proximal promoter region in order to fully understand the role of the sequence elements present and to define the core promoter region. Two Sp1 sites downstream of the CCAAT-box mediated negative regulation, while the other two Sp1 sites were positive regulators with the strongest effect mediated by the negative regulatory Sp1 site closely flanking the CCAAT box. Basal transcriptional activity is maintained via the CCAAT-box even when all four Sp1 sites are mutated, suggesting NF-Y is a fundamental regulator of hTR promoter function. Chromatin immunoprecipitation revealed binding of NF-Y, Sp1 and TFIIB to the promoter *in vivo*, providing the first direct evidence that a component of the Pol II transcriptional machinery is involved in telomerase RNA gene transcription in human cells. Thus the interaction of NF-Y at the CCAAT-box is pivotal to hTR gene transcription and surrounding sequence elements may provide an environment for the regulation of activity through recruitment of additional protein complexes.

**P59**

HYPOXIC REGULATION OF TELOMERASE GENE EXPRESSION

Claire J. Anderson*, Margaret Ashcroft, W. Nicol Keith
Cancer Research UK Department of Medical Oncology, University of Glasgow, Cancer Research UK Beatson Laboratories, Garscube Estate, Switchback Road, Bearsden, Glasgow, G61 1BD

Expression of the telomerase RNA (hTR) and protein (hTERT) component genes is greatly up regulated in cancer cells when compared to normal cells and this up-regulation occurs at the level of transcription. To determine how this occurs we are investigating a number of transcriptional regulatory elements within the telomerase gene promoters. Bioinformatic analysis of the telomerase promoter sequences identified potential hypoxia inducible sequence elements: 2 within hTERT overlapping the E-box consensus sequences and 1 within hTR. Gel retardation assays using labelled oligonucleotides encompassing these putative hypoxia response elements (HRE) revealed protein binding. Competition studies showed specific binding of HIF-1 and myc to these sites. Transfection of A2780 ovarian carcinoma cells with the telomerase promoters linked to luciferase reporter constructs allowed for functional analysis of these sites. Co-transfection of telomerase promoter vectors with a titration of HIF-1α expression vector gave a 2-fold increase in expression when compared to telomerase promoters alone. This effect is specific, as co-transfection of HIF-1α with hTERT promoter mutated in both consensus HRE sites produces no effect. These results suggest that telomerase gene expression is inducible by hypoxia, which may have implications for tumour development and gene therapy.

**P60**

CISPLATIN INDUCED NEUROBLASTOMA CELL DEATH AND THE INVOVLEMENT OF TELOMERES

Jessie C. Jeyapalan *[1,2], Michael J. Tilby [2], Thomas von Zglinicki [1]
[1]Department of Gerontology SCMS, University of Newcastle upon Tyne, Newcastle General Hospital, NE4 6BE
[2]Northern Institute of Cancer Research, University of Newcastle upon Tyne, Medical School NE2 4HH

**Background:** Neuroblastoma is chemotherapeutically treated by cisplatin whose major DNA adduct is the intrastrand crosslink between adjacent guanines. Human telomeric DNA is partially composed of guanines as its sequence is TTAGGG. Telomere damage accelerates shortening which may signal growth arrest and/or apoptosis. A specific role of telomeres in mediating cisplatin cytotoxicity has been proposed.

**Aim:** To establish the significance of the telomere-cisplatin interaction in the neuroblastoma cell line SHSY5Y.

**Methods:** Cytotoxic effects of cisplatin were assessed by the SRB assay, cell cycle analysis and apoptosis measurements using flow cytometry. Telomere and single stranded G rich overhangs were detected using an in gel hybridisation technique.

**Results:** A 2 hour exposure to cisplatin at concentrations at or below the IC50 value (15μM) induced low levels of apoptosis. Following exposure to high concentrations of cisplatin (>350μM) apoptosis increased dramatically by 24 hours after treatment. Also there was no net growth of cells and telomerase inhibition occurred. There was no change in telomere lengths or single stranded overhangs after a two hour exposure to cisplatin following any of the cisplatin concentrations tested.

**Conclusions:** Telomere lengths do not decrease after a two hour exposure or a continuous treatment to cisplatin. SHSY5Y apoptosis induction after cisplatin treatment is not mediated by telomere shortening.

© 2003 Cancer Research UK

**P61**

SYNTHESIS, MODELLING AND BIOPHYSICAL STUDIES OF AMINO-ANTHRAQUINONES AS TELOMERASE INHIBITORS

Toni Brown*, Roz J. Anderson, Don Cairns, Terry C. Jenkins. YCR Laboratory of Drug Design, Tom Connors Cancer Research Centre, Bradford, West Yorkshire BD7 1DP.

Functionalised amino-anthraquinones (AAQ) are being examined as selective ligands to stabilise four-stranded (tetraplex) DNA structures. Such complexes would prevent access of telomerase to its linear DNA substrate and thereby elicit a possible chemotherapeutic response for tumour control. Certain AAQs have previously been examined as intercalators for duplex DNA in the quest for cytotoxic agents.

Uncyclised and cyclised 1-mono-, 1,5-di- and 1,8-di-AAQs of different complexion have been synthesised with differing side-chains: $-NH(CH_2)_2OH$, $-NH(CH_2)_2NH(CH_2)_2OH$ and $-NH(CH_2)_2N(CH_2CH_3)_2$. A cleaner, flexible route to these compounds has been developed and will be presented.

Binding energies for novel cyclised compounds have been calculated using molecular modelling with two reported [NMR and crystal] DNA-tetraplex structures. Early results show that energies appear to be superior for the NMR rather than the crystal structure (~-700 *vs* ~-400 kcal $mol^{-1}$) using one selected quinoxaline-functionalised AAQ ligand.

Preliminary DNA melting experiments show stabilisation of calf thymus DNA, (DNA duplex-drug ratio 10:4) for uncyclised compounds with negligible effect for the cyclised AAQ series. *e.g.* 1-[2-(2-hydroxyethylamino)ethylamino]anthraquinone; $\Delta T_m$ =10 °C and 4-(2-hydroxyethyl)-1,2,3,4-tetrahydro-1,4-diaza-benzo[*a*]anthracene-7,12-dione; $\Delta T_m \leq 1$ °C. Full DNA-binding profiles and biological data will be presented.

**P62**

QUANTIFICATION OF CTL RESPONSE AGAINST hTERT IN BREAST CANCER

Shoba M.P. Amarnath*, Charlotte E. Dyer, Aswatha Ramesh, Obi Iwuagwu, Liviu V. Titu, Philip J. Drew, John Greenman. Postgraduate Medical Institute, University of Hull.

Telomerase is expressed in approximately 90% of breast cancers but not in most normal somatic cells. The enzyme plays a key role in maintaining chromosomal telomere integrity. The catalytic subunit of telomerase, human telomerase reverse transcriptase (hTERT), is a widely expressed tumour associated antigen against which specific cytotoxic T lymphocyte (CTL) responses have been identified. Human $CD8^+$ CTL have been shown to recognise hTERT synthetic peptides in both an HLA-A2 and HLA-A3 restricted manner, and to lyse $hTERT^+$ tumour cells of multiple histologies.

This study has investigated the specific $CD8^+$ CTL response in breast cancer patients against 3 synthetic hTERT peptides. PBMC were isolated from blood samples by Ficoll-density centrifugation and screened for HLA subtype by flow cytometry. The HLA-A2 or HLA-A3 positive PBMC samples were analysed using a standard IFNγ ELISpot assay.

The results showed that 11/15 cancer patients ($HLA-A2^+$ or $HLA-A3^+$) had a specific $CD8^+$ CTL response against one of the synthetic hTERT peptides; five of these patients responded to both HLA-A2 peptides. In contrast, 4/11 normal healthy female controls responded against a single peptide only. This suggests that a specific immune response exists against hTERT peptides in breast cancer patients when compared with normal healthy controls. Such functional immune response assays indicate that hTERT deserves further investigation as a target for anticancer immunotherapeutic strategies in breast cancer patients.

Financial support provided by RCS(Eng) and an Overseas Research Scholarship (S. Amarnath)

**P63**

THE DEVELOPMENT OF NOVEL TELOMERASE INHIBITORS: STRUCTURE-ACTIVITY RELATIONSHIPS

Mai-Kim Cheng*, Chetna Modi, Christina R. Grindon, Malcolm F.G. Stevens and Charles A. Laughton. Cancer Research Laboratories, School of Pharmaceutical Sciences, University of Nottingham, NG7 2RD.

We have discovered and are developing a novel class of polycyclic acridines that show great promise as inhibitors of telomerase. The hypothesis is that these molecules act by stabilizing the G-rich single-stranded overhang of the telomere in a four-stranded (quadruplex) form, in which it is unable to function as a substrate for elongation by the telomerase enzyme.

Therapeutically acceptable telomerase inhibitors that function through this mechanism must show a high specificity for the quadruplex form of DNA. Otherwise, they will be expected to also show the undesirable cytotoxic characteristics of conventional antitumour agents that target normal genomic (duplex) DNA.

We have studied the binding of a wide range of our polycyclic acridines to both duplex and quadruplex DNA, and correlated the results with their telomerase inhibition (TRAP assay) and cytotoxicity ($GI_{50}$ data from the NCI 60 cell panel). Binding affinities of the molecules to salmon testis DNA (fluorescence titration experiments) correlate excellently with the $GI_{50}$ data ($R^2$ 0.925) but not at all with telomerase inhibition. Conversely, the ability of the molecules to stabilize quadruplex DNA (FRET-based melting studies) correlates fairly well with the TRAP assay data ($R^2$ 0.677) but not at all with the $GI_{50}$ data. Associated molecular modelling studies suggest that quadruplex versus duplex selectivity is controlled by a subtle balance between many factors, including solvation, electrostatics, and DNA flexibility. These results support the proposed mechanism of action of these compounds and are guiding their further development.

**P64**

ZONATION OF MUCOSAL PHENOTYPE, DYSPLASIA AND THEIR RELATIONSHIP WITH TELOMERASE ACTIVITY IN BARRETT'S OESOPHAGUS

Aileen J. Fletcher-Monaghan*, James J. Going, Lisa Neilson, Bea A. Wisman, Ate van der Zee, Robert C. Stuart, W. Nicol Keith Cancer Research UK Department of Medical Oncology, University of Glasgow, Cancer Research UK Beatson Laboratories, Garscube Estate, Swittchback Road, Bearsden, Glasgow, G61 1BD

Unchecked neoplastic growth requires telomerase enzyme activity. We have investigated the zonal distribution of different mucosal types and dysplasia in Barrett's oesophagus in relation to telomerase activity. Patients and methods Biopsies (n 256) from squamous oesophagus, columnar-lined oesophagus every 2 cm, oesophago-gastric junction, gastric corpus and antrum were evaluated from 32 patients with ≥3cm Barrett's oesophagus by telomerase repeat assay protocol (TRAP) and reviewed for mucosal phenotypes and severity of dysplasia. Results Intestinal-type Barrett's mucosa was consistently present at all levels in Barrett's oesophagus, and at least one Barrett's biopsy was TRAP+ in 22/32 patients. The data were consistent with a proximal-to-distal gradient of increasing TRAP-positivity of intestinal-type Barrett's mucosa, possibly related to mucosal exposure to acid or bile reflux. Native gastric mucosa was rarely TRAP+ (1/31 corpus, 2/32 antrum), whereas native squamous mucosa was almost always TRAP+ (31/32). A non-significant association between TRAP positivity and dysplasia existed, but the TRAP assay could be positive without dysplasia and negative even in extensive, high-grade dysplasia.

© 2003 Cancer Research UK

### P65

A PRELIMINARY EVALUATION OF THE POTENTIAL ROLE OF FDG-PET TO ASSESS RESPONSE TO GLIVEC™ IN GASTROINTESTINAL STROMAL TUMOURS (GISTS)

Jackie Harney*, Way lup Wong, Susan Short. MCRW, Paul Strickland Scanner Centre, Mount Vernon Hospital, UK.

**Introduction:** GIST's are the commonest mesenchymal tumours of the GIT. They are characterised by over-expression of KIT, the protein product of the c-kit proto-oncogene, & unresponsiveness to any type of systemic chemotherapy. Glivec™ (Imatinib) is a new type of tyrosine kinase inhibitor that selectively inhibits various tyrosine kinases including KIT. Glivec™ shows a remarkable efficacy in the treatment of GIST's [1]. It is currently available in theUKon a named patient basis for advanced GISTs. If metabolic change occurs before morphologic change following treatment, traditional imaging modalities (CT & MRI) cannot detect it. FDG-PET has been shown to highlight early functional changes in glucose metabolism that correlate with metabolic tumour response to Glivec™ [2]. **Methods:** 4 patients with advanced, metastatic GISTs presenting to MVH between March 2002 and March 2003 were commenced on Glivec™ 600 - 800mg/day. MRI and FDG-PET scans were performed prior to treatment. FDG-PET was repeated after 2-4 weeks of treatment to assess early response. MRI scanning was performed at regular intervals thereafter.

**Results**: In this group of patients FDG-PET at 2-4 weeks demonstrated marked metabolic improvement, which correlated well with symptomatic improvement. MRI scanning at 6-8 weeks demonstrated partial response to treatment in all patients.

**Conclusions:** Reduced FDG uptake on PET scanning at 1 month appears to predict responses on MRI and herald clinical improvement in this small group of patients. **References:**

1. Demetri GD et al *N. Eng. Med.* 2002; **347:472**-480.
2. Van den Abbeele et al. ASCO 2001. Sarcoma abstract1444.

### P66

EVALUATION OF MULTI-FUNCTIONAL DYNAMIC CONTRAST-ENHANCED MRI TO PREDICT RESPONSE TO NEOADJUVANT CHEMOTHERAPY IN PRIMARY BREAST CANCER

Mei-Lin W. Ah-See*, N. Jane Taylor, Andreas Makris, Russell J. Burcombe, James J. Stirling, Helen J. Cladd, Martin O. Leach, Anwar R. Padhani; Mount Vernon Hospital, Northwood, Middlesex, HA6 2RN.*

**Introduction:** Neoadjuvant chemotherapy (NAC) is increasingly used for treating primary breast cancer. The ability to identify non-responders early during treatment will enable the use of alternative therapies that may be more beneficial. Here, we assess the role of multi-functional dynamic contrast-enhanced magnetic resonance imaging (MRI) to identify these non-responders**. Materials & Methods:** 10 patients with primary breast cancer (median age 44 years, range 33-59) were imaged prior to & following two cycles of 5-fluorouracil, epirubicin & cyclophosphamide chemotherapy. Dynamic MRIs were obtained following contrast-medium administration (Gd-DTPA) & parameters reflecting microvessel permeability (Ktrans), blood flow (rMSD), leakage space (ve) & oxygenation (R2*) were measured. Median & 5-95th centile values for each parameter were derived from tumour regions of interest. Pre-treatment parameter values & treatment changes were correlated with clinical response following 6 cycles of NAC. **Results:** Pre-treatment Ktrans, rMSD, ve & R2* did not predict for response. Percentage change in median Ktrans & rMSD correlated with response (p=0.04 & p=0.04 respectively) as did percentage change in the Ktrans 5-95th centile range (p=0.04). Changes in ve & R2* were not significant. **Conclusion:** These early data indicate that MRI parameters reflecting blood flow & vascular permeability may act as early response parameters for NAC in breast cancer. A large patient cohort is currently being studied to quantify the magnitude of this effect.

### P67

MAGNETIC RESONANCE SPECTROSCOPY (MRS) STUDY OF 17AAG IN HT29 XENOGRAFTS

Helen Troy[1*], Yuen-Li Chung[1], Udai Banerji[2], Ian R Judson[2], Martin O Leach[2], Marion Stubbs[1], Sabrina Ronen[2], Paul Workman[2] and John R Griffiths[1]
[1]St George's Hospital Medical School, London, UK, [2]Institute for Cancer Research, Sutton, UK.

**Aims**: 17AAG is an anti-cancer drug that inhibits heat shock protein 90 (Hsp90) which results in proteasomal degradation of oncogenic client proteins. The aim of this work was to develop a non-invasive pharmacodynamic (PD) marker for 17AAG.

**Methods**: HT29 xenografts were grown in nude mice to a size of 484$\pm$36 mg. *In vivo* localized $^{31}$P MRS was carried out on day 1 (pre-) and 5, on tumours treated with 17AAG (n = 14, 80mg/kg i.p., 4 days) or EPL vehicle (n = 5). *In vitro* $^{1}$H and $^{31}$P MRS and Western blots for Raf-1 and Hsp70 were carried out on tumour extracts to complement our *in vivo* MR data.

**Results:** 17AAG inhibited tumour growth (97 $\pm$ 2%), while control tumours increased by 120 $\pm$ 7% of pre-treatment volume. *In vivo* $^{31}$P MRS showed a significant reduction in β-NTP/total phosphorus signal (TotP) ($p < 0.03$) and elevation in phosphocholine and phosphoethanolamine (PC+PE)/TotP ($p < 0.05$) ratios in 17AAG-treated tumours. No significant changes were found in controls. *In vitro* $^{31}$P and $^{1}$H MRS of the 17AAG-treated tumour extracts confirmed our *in vivo* findings, showing a significant increase in the levels of PC ($p < 0.04$) and PE ($p < 0.03$) when compared with controls. Western blots showed induction of Hsp70 and reduced Raf-1 in the 17AAG-treated group, confirming the expected action of the drug.

**Conclusions**: 17AAG inhibited Hsp90 and altered tumour bioenergetics and phospholipid membrane metabolism, as well as decreasing tumour growth rate. MRS may provide a non-invasive PD marker for use in clinical trials.

### P68

DETERMINATION OF THE ANTI-CANCER DRUG ACTINOMYCIN D IN HUMAN PLASMA BY LIQUID CHROMATOGRAPHY-MASS SPECTROMETRY,

Julie Errington*, Gareth J. Veal, Julieann Sludden, Melanie J. Griffin, Lisa Price, Annie Parry, Juliet Hale, Andrew D.J. Pearson and Alan V. Boddy. Northern Institute for Cancer Research, University of Newcastle upon Tyne, Newcastle upon Tyne NE2 4HH.

Actinomycin D is an anticancer drug commonly used in the treatment of paediatric malignancies such as Wilms tumour and Ewing's sarcoma. Despite its long history of clinical use, little is known about the pharmacokinetics of actinomycin D in humans. As actinomycin D treatment in children with cancer is associated with veno-occlusive disease, and as dose intensity has been defined as a significant risk factor for the development of this potentially life-threatening toxicity, pharmacokinetic studies of actinomycin D may be beneficial in optimizing treatment with this drug.

We have developed an accurate and sensitive liquid chromatography-mass spectroscopy method for the determination of actinomycin D in human plasma samples. Extraction of analytical samples was carried out with acetonitrile and analysis performed on a $C_8$ column with an API 2000 LC/MS/MS using an internal standard of 7-aminoactinomycin D.

The method showed good reproducibility with intra- and inter-assay precision CVs of 2.7-11.3% and 2.3-7.8% respectively. Actinomycin D recovery rates varied from 83.5 to 89% and the limit of detection was determined to be 1ng/ml. Analysis of plasma samples obtained from 2 patients receiving actinomycin D treatment, indicated that the assay could successfully be used to quantify actinomycin D in clinical samples. Peak plasma concentrations of 7.71 and 41.3ng/ml were observed following doses of 0.75 and 1.5mg/m$^2$ respectively.

© 2003 Cancer Research UK

**P69**

SENSITIVE DETERMINATION OF BBR 3464 AND CISPLATIN DNA ADDUCT LEVELS IN CLINICAL SAMPLES USING ICP-MS and PIMMS

Tyrone John[*1], Chris Ottley[2], Graham Pearson[2], Geoff Nowell[2], Hilary Calvert[1] & Michael Tilby[1]. [1]NICR, Newcastle University, Framligton Place, NE2 4HH. [2]Durham University, Geology Department.

Introduction. Blood samples from recent clinical trials of the novel trinuclear platinum anti-cancer drug BBR3464 provide the opportunity to determine the levels of Pt-DNA adducts achieved in patients with this drug. Since immunoassays are not available for these adducts, highly sensitive inductively coupled plasma mass spectrometry (ICP-MS) methods have been developed for Pt on DNA.

Methods Whole blood was incubated with BBR 3464, 1hr, 37°C, and lmphocytes isolated (lymphoprep). A2780 ovarian cells were exposed to BBR 3464 and cisplatin (1h, 37°C). $IC_{50}$ values were determined by XTT. DNA was extracted (Qiagen tips) and Pt levels determined with ThermoFinnegan Neptune (PIMMS) and Perkin Elmer Elan 6000 quadrapole ICP-MS instruments.

Results and conclusion Adduct levels at $IC_{50}$ exposures of BBR 3464 and cisplatin in A2780 were 2.5 and 2.8 nmoles /gDNA respectively and gave signals >100x above background. DNA adduct levels detected in blood incubated with levels of BBR3464 observed in patients (0.03 μM) were about 0.18 nmoles/gDNA. PIMMS gives >50x Pt sensitivity than ICP-MS allowing the accurate analysis of blood DNA adducts. If BBR 3464 adducts are formed in patients at biologically effective levels they should be detectable by these techniques.

**P71**

DEXRAZOXANE (DXRz) DOES NOT REDUCE THE RESPONSE RATE TO DOXORUBICIN (ADRIAMYCIN) Kurt Hellmann* Windleshaw House, Withyham, East Sussex, TN7 4DB, UK

Reduction of side effects of antitumour drugs with-outloss of activity has been explored for many years. Although highly active, doxorubicin's full potential is restricted by a dose limiting cardiotoxicity. DXRz is highly effective in preventing this potentially lethal side effect. It is not accompanied by any new adverse reaction apart from a reduction in WBC. One of 16 randomised, controlled clinical trials reported a reduced response rate. It could not be repeated in an identical trial. Survival has not been reduced in any of the 16 trials. In an attempt to find an explanation for the apparent reduction in response, we examined the effects of DXRz on K562 cells.

We found that as with razoxane, incubation in $IC_{50}$ levels of DXRz (70uM) led to G2/M arrest within 8h. At 24h, 35± 6% of cells had tetraploid or higher DNA content, suggesting DNA rereplication. The cells were also significantly larger (mostly twice as large). If this occurred in a solid tumour, it would be possible for a tumour to increase in size without any increase in number of cells and thus give the illusion of not having responded.

Differences of response rate between controls and treated as in the 1/16 trials (above) cannot be taken at face value, but should be rigorously examined, particularly where differences are small (as above) and in accordance with FDA ruling that response rate is just a surrogate for the all important median survival time.

**P70**

METHIONINE DEPENDENCY OF TUMOURS: A BIOCHEMICAL STRATEGY FOR OPTIMIZING CHEMOSENSITIVITY. V Pavillard*, DJ Swaine, AA Drbal, RM Phillips, A Nicolaou, & JA Double. Tom Connors Cancer Research Centre and School of Pharmacy, University of Bradford, Bradford BD7 1DP, UK.

The aim of our study is to explore the effect of MET depletion on viability, cell cycle kinetics, sulfur amino acid metabolism in MET-dependent cancer cells for optimizing *in-vitro* chemosentivity. The cells used were rat hepatocarcinoma HTC (MET-dependent), Phi-1 (partially MET-independent), transformed mouse fibroblasts 3T3 (MET-dependent) and human skin fibroblasts Hs-27 (MET-independent). Cells were grown in Control medium (containing MET) and MET depleted medium (containing HCY); viability was tested by supplying MET after 5 days of MET depletion. In MET depleted medium, MET-dependent cells did not proliferate and were accumulated in S/G2 phases of the cell cycle compared to controls. They remained viable after MET depletion that resulted in cell proliferation, transient burst of cells in S and G2/M phases and normalization of cell cycle parameters few days later. Cysteine was taken up by HTC while 3T3 exported HCY. MET independent cells were able to grow MET depleted medium without significant changes in cell cycle parameters compared to controls. Cysteine was taken up by Phi-1 while Hs-27 exported HCY. The amino-acid metabolism seemed to be cell-type dependent. The cells treated with vinblastin and taxol (targeting cells in G2/M phase) were all more resistant to both drugs under MET depletion compared to controls (treatment in control medium). Only MET-dependent cells were more sensitive to both drugs while recovering after MET depletion compared to controls and MET-independent cells. The potential of synchronizing MET-dependent cells in a specific phase of the cell cycle is rendering cells more susceptible to cell cycle phase specific agents and produces a therapeutic gain. This work was supported by Cancer Research UK.

**P71a**

COMPARISONS OF HISTOPATHOLOGY, MOLECULAR GENETICS AND SINGLE VOXEL $^1H$ MR SPECTROSCOPY IN OLIGODENDROGLIAL NEOPLASMS

Carol Walker[+*], Trevor Smith[#], Kathyrn A. Joyce[+], Daniel DuPlessis[#] and Peter C. Warnke[#] [+]Clatterbridge Cancer Research Trust, Clatterbridge Hospital, Bebington, Wirral, CH63 4JY; [#]Walton Centre for Neurology and Neurosurgery, Liverpool, UK.

The diagnosis of gliomas is particularly challenging with the histopathological classification frequently differing from a classification based on molecular genetics. $^1H$-MRS is capable of non-invasively determining intracellular metabolite concentrations, which in brain tumours differ from normal brain, but it is not known whether this technique can distinguish the different subtypes of glioma based on either a molecular or a histopathological classification.

In this study, 28 tumours were investigated and laser capture microdissection and microsatellite analysis was used to determine allelic losses. Loss of both 1p36 and 19q13, typical of oligodendrogliomas, was found in 8/12 OII, 3/10 OAII, 2/3 OIII and 1/3 OAIII. $^1H$ single voxel spectra were obtained using a G.E. Signa 1.5T NVi MR Scanner running PROBE P (PRESS) SV MRS TR 1500 TE 35. Peak areas of NAA, Cho, Cr, myoinositol and lipid/lactate were calculated and normalised to the Cr peak. No significant difference in NAA, Cho, myoinositol, lipid/lactate or NAA/Cho was observed in oligoastrocytomas vs oligodendrogliomas, or tumours with 1p36/19q13 loss vs those without, when the data was analysed in the series as a whole or in low-grade tumours only. Despite the small number of high-grade cases, Cho and lipid/lactate signals were significantly greater in high-grade vs low-grade tumours.

In gliomas with an oligodendroglial component, single voxel $^1H$-MRS may be useful to determine malignancy, but in this study did not permit distinction between histological or molecular subtypes.

 © 2003 Cancer Research UK

**P72**

PROGNOSTIC SIGNIFICANCE OF OESTROGEN RECEPTOR BETA IN USUAL DUCTAL HYPERPLASIA OF THE BREAST

Abeer M. Shabaan , Penny A. O'Neill* and Christopher S. Foster
Department of Cellular and Molecular Pathology, The University of Liverpool, L69 3GA, UK

ERβ is thought to have prognostic significance in breast cancer. However, little is known about its role in benign proliferative breast lesions. We designed case-control study on 120 cases who had benign breast biopsies that progressed to breast cancer and 382 matched controls that did not develop breast cancer (follow-up: 20yrs). Foci of usual ductal hyperplasia, (UDH, n=154) and adjacent normal lobules (n=116) were identified and stained with monoclonal ERβ and ERα monoclonal antibodies. The majority of normal lobules from cases that developed breast cancer and controls expressed ERβ with a significantly higher level in control subjects ($P$=0.04). ERβ expression in UDH from cases (67.50%) was lower than that of matched controls (80.50%). This difference, however, was not statistically significant, whereas ERα level was higher in cases than in controls ($P$=0.008). A significantly lower median expression of ERβ was seen in UDH foci from control patients when compared with adjacent morphologically normal lobules ($P$<0.001). ERβ:ERα ratio was significantly lower in UDH from cases than in controls ($P$=0.008). Our date indicate that the level and distribution of ERβ are distinct from those of ERα. Concurrent low levels of ERβ and high levels of ERα characterise potentially high-risk patients. The relative occurrence and/or interaction of these two types of oestrogen receptor might help predict the phenotypical behaviour of UDH.

**P73**

INFLUENCE OF CYTOKINE GENE POLYMORPHISMS ON SUSCEPTIBILITY TO AND PROGNOSIS IN BREAST CANCER

W. Martin Howell*, Katherine C. Smith, Helen M. Fussell, Adrian C. Bateman. Departments of Molecular Pathology, Cellular Pathology and University Human Genetics, Southampton General Hospital, Southampton, SO16 6YD, UK.

Single nucleotide polymorphisms (SNPs) in the promoter regions of a number of cytokine genes are associated with differential levels of cytokine expression *in vitro*. In accordance with our findings in other malignancies, we hypothesised that these SNPs might influence breast tumour development and progression by affecting the efficiency of the anti-tumour immune response and/or pathways of angiogenesis.

144 female breast cancer patients and 263 cancer-free controls were genotyped for selected pro- and anti-inflammatory and angiogenic cytokine SNPs, including IL-1β –511 (T/C), IL-6 –174 (G/C), TNFα–308 (A/G), IL-10 –1082 (A/G), IL-8 –251 (A/T) and VEGF –1154 (A/G), using ARMS-PCR and TaqMan® 5' nuclease assays for allelic discrimination. Patient-control comparisons revealed that the TNFα –308 GG genotype was increased in patients v controls (79.7% v 68.2%, $P$=0.03, OR=1.83, 95% CI=1.08-3.09). Stratification of the patient group according to the Nottingham Prognostic Index revealed a number of significant associations, including IL-8 –251 AA and poor prognosis disease (35.3% v 12.7%; $P$=0.02) and IL-6 -174 GC and Grade 2/3 v Grade 1 disease (51.3% v 31.4%; $P$=0.04).

These results provide preliminary evidence that polymorphisms in the promoter regions of cytokine genes may affect both susceptibility to and prognosis in breast cancer, although causality cannot be directly inferred. Confirmation of these findings is currently being sought in an expanded patient and control group, with full SNP exclusion for selected genes.

**P74**

CORRELATION OF WILD TYPE OESTROGEN RECEPTOR BETA (ERβ1) mRNA AND PROTEIN EXPRESSION WITH PROGNOSIS IN TAMOXIFEN TREATED BREAST CANCER.

Penny A.O'Neill[1*], Abeer M. Shaaban[2], Christopher S.Foster[2], Helen Innes[1], D. Ross Sibson[1], and Michael P.A. Davies[1]
[1]Clatterbridge Cancer Research Trust, J. K. Douglas Laboratories, Wirral, and [2]Dept. of Cellular and Molecular Pathology, University of Liverpool, Merseyside.

ERα status is important for predicting response to hormonal therapies but the role of ER β is less clear. We studied expression of ERα and ERβ1 proteins in 167 patients with invasive breast cancers treated with adjuvant endocrine therapy. We used a previously validated PPG5/10 monoclonal antibody to detect full length, wild type ERβ1. The results were compared with mRNA expression of the same genes by RT-PCR.

ERα protein expression was closely associated with RNA expression detected by RT-PCR (p< 0.001). However ERβ1 protein was expressed at a variety of levels and did not correlate with RNA expression. Associations were seen between expression of both receptors by RT-PCR (p < 0.001) and between ERβ RT-PCR and ERα IHC (p < 0.027), but not between the ERα and ERβ1 proteins detected by IHC. It is therefore possible that translational or post-translational control plays a significant part in ERβ1 expression. There was no significant association between ERβ1 IHC status and PgR status, tumour stage or grade, axillary nodal status, presence of lymphovascular invasion, size of tumour or proliferation. There was a trend for ERβ1 IHC negative cases to have a better outcome both within the group as a whole and within ERα positive Tamoxifen-treated cases.

**P75**

DETECTION OF CIRCULATING TUMOR CELLS IN BLOOD AND BONE MARROW OF PATIENTS WITH GASTROINTESTINAL CANCERS

Sergej P. Osinsky*, Vitalij V. Lukyanchuk, Antoine Roggo, Jorg Kleeff, Daniil F. Gluzman, Vyacheslav A. Chorny, Dmitry S. Osinsky, Daniel Candinas, and Helmut Friess.
Institute of Exp. Pathol. Oncol. Radiobiol., Kiev, Ukraine; Bern University, Switzerland; Heidelberg University, Germany.

**Aim:** to examine whether cytokeratin 20 (CK-20) and prostate stem cell antigen (PSCA) are useful markers for the detection of disseminated cancer cells.

**Patients and Methods:** nested RT-PCR was used to determine the expression of CK-20 and PSCA in peripheral blood (PB) from 47 patients (pts.) with pancreatic carcinoma (PC) (11), gastric cancer (8), colorectal carcinoma (15), and miscellaneous tumors (13). Immunocytochemical method was used to detect the expression of CK in bone marrow (BM) from patients with PC (11). All patients and healthy volunteers (18) gave written consent before samples were obtained.

**Results:** CK-20 expression was observed in PB of 19/47 (40.4%) pts. with malignant tumors (MT). PSCA expression was present in PB of 22/47 (46.8%) pts. MT, and particularly in 7/11 (63.6%) patients with PC. CK-20 and PSCA expression was not observed in blood samples from healthy donors. CK- or PSCA-positive cells were found in 40% of Stage II-III patients. There was a relationship between PSCA expression and tumor stage. Correlation between findings of cancer cells in BM and survival was shown. Reactive changes of immunocompetent and stromal cells in BM of patients with $CK^+$ cells were observed.

**Conclusion:** Detection of cancer cells in PB and BM can be useful both to identify the patients of high risk for early metastases and prognosis of clinical outcome.

The study was supported by SCOPES (Grant No 7 IP 62587).

**P76**

INTERLEUKIN 10 POLYMORPHISM MAY INFLUENCE RENAL CANCER DEVELOPMENT.

Erik G. Havranek *, Mike Whelan[a], Joseph Whelan[a], Angus Dalgleish[b], Chris Anderson, Hardev Pandha[b]

Department of Urology, St. George's Hospital, London.
[a] Onyvax Ltd, St. George's Hospital Medical School, London.
[b] Department of Clinical Oncology, St. George's Hospital Medical School, Cramner Terrace, London SW17 ORE.

Introduction: Interleukin-10 (IL-10) is a T-helper 2 cytokine with known anti-inflammatory properties. A gene polymorphism of the IL10-1082 promoter region causes low expression of IL-10. This study aimed to determine whether there is an association between this IL-10 polymorphism and renal cancer.

Patients and Methods: The single nucleotide polymorphism in the IL10-1082 gene was detected by TaqMan real-time PCR using probes labelled with FAM or VIC dyes. Genotypes were assigned to DNA extracted from paraffin embedded archival tissue from patients (n=147) with known renal adenocarcinoma. These were then compared to DNA extracted from peripheral blood lymphocytes of control patients (n=149) without cancer.

Results: Of the renal cancer patients, 44% (n=26) had the AA genotype, 38% (56) AG and 18% GG. Of the control patients 30% (45) were AA, 46% (69) AG and 23% (35) GG. Chi square test showed a significant difference between cancer and control patients ($p<0.05$). The odds ratio (1.83) shows that the AA genotype is over-represented in renal cancer patients with a 95% confidence interval 1.14-2.95.

Conclusions: The results suggest that there is a significantly larger proportion of low IL-10 (AA) producing homozygotes amongst renal cancer patients than would be expected in a normal population. This data is in agreement with the findings in prostate cancer patients.

**P77**

EVALUATION OF PLASMA & SERUM VASCULAR ENDOTHELIAL GROWTH FACTOR (VEGF) DURING NEOADJUVANT CHEMOTHERAPY FOR PRIMARY BREAST CANCER.

Mei-Lin W.Ah-See*, Andreas Makris, Russell J. Burcombe, Charlotte Davies, Helen J. Cladd,Anwar R. Padhani & Adrian L. Harris. Mount Vernon Hospital,Northwood, Middlesex, HA6 2RN.*

**Background:** There is a need for surrogate markers of angiogenic activity. VEGF is an important regulator of angiogenesis & elevated tumour VEGF is a poor prognostic factor in breast cancer. We measured plasma & serum VEGF (pVEGF,sVEGF) during neoadjuvant FEC chemotherapy (NAC) for primary breast cancer in order to evaluate its role as a predictor of tumour response to NAC. **Materials & methods:** Serial measures of pVEGF & sVEGF were performed in 22 patients (median age 47, range 34-67) undergoing NAC for T2-4 biopsy-proven breast carcinoma. Samples were collected before cycle 1, 2, 4 & 6 of treatment and measured using an ELISA immunoassay. Baseline & changes in pVEGF & sVEGF were correlated with clinical, radiological & pathological response. **Results:** Little inter-or intra-patient variability was observed in pVEGF or sVEGF & no consistent variation with time could be demonstrated. There was no significant difference in baselineor change in pVEGF or sVEGF afterone cycle of treatment between responders & non-responders classified by clinical, radiological or pathological criteria. **Conclusions:** Given that baseline & changes in pVEGF & sVEGF are of limited value in predicting response to neoadjuvant chemotherapy alone in breast cancer, changes following combination therapy (chemotherapy & antiangiogenic therapies) may be of value as a surrogate marker of angiogenic activity specifically inresponse to antiangiogenic therapies.

**P78**

PROGNOSTIC VALUE OF COMPLEMENT REGULATORY PROTEINS CD55 AND CD59 IN BREAST CANCER

Zahra Madjd[1]*, Sarah E Pinder[2], Ian Spendlove[1] and Lindy G Durrant[1] [1]CRUK Clinical Oncology and [2] Dept. of Histopathology, University of Nottingham, City Hospital, Nottingham, NG5 1PB

**Introduction:** Cells express complement regulatory proteins CD55, CD59 and CD46 to protect them from bystander attack by complement. Our previous immunohistochemical study showed that in breast carcinoma loss of CD59 correlated with poor survival.
**Aim and methods:** The prognostic significance of CD55 was investigated in 480 patients with primary operable breast cancer, using an anti-CD55 monoclonal antibody (mAb) RM1 (developed in the department) with a standard immunohistochemistry method. The anti-CD55 mAb (RM1) was raised against a synthetic peptide and stained formalin-fixed, paraffin embedded sections stronger than the commercial mAb to CD55 (clone 67). As there are no commercially available anti-CD46 antibodies suitable for use on paraffin sections we are developing a peptide specific antibody to CD46. This will be used to complement the antibodies to CD55 and CD59 in screening the breast microarrays.
**Results:** 95% of the breast carcinomas expressed CD55 (RM1) with intensity ranging from weak (51%) to strong (6%). High expression of CD55 was significantly associated with low grade (G1, G2) ($p=0.001$), lymph node negativity ($p=0.031$) and good prognosis tumours (Nottingham Prognostic Index <3.4) ($p<0.001$). Significant correlation was also found between expression of CD55 and overall survival, loss of CD55 in breast tumours being associated with poor survival ($p=0.001$).
**Conclusion:** Our data indicated that unexpectedly loss of CD55 and CD59 is associated with aggressive breast tumours. However, it has been speculated that loss of CD55 and CD59 may be compensated by increased expression of CD46, which inhibits the complement cascade at an early stage. Analysis of CD46 expression in these breast microarrays will complete the picture of the role of these complement inhibitory proteins in tumour prognosis.

**P79**

PEROXISOME-PROLIFERATOR ACTIVATED RECEPTOR-GAMMA (PPARG) AND THE PPARG CO-ACTIVATOR, PGC-1, AND THEIR CORRELATION WITH AGGRESIVENESS OF HUMAN BREAST CANCER.

Wen G. Jiang[1], Anthony Douglas-Jones[2] and Robert E. Mansel[1], [1]Metastasis Research Group and [2]Department of Pathology, University of Wales College of Medicine, Heath Park, Cardiff, The United Kingdom

Peroxisome-proliferator activated receptor-gamma (PPARγ) belongs to a family of nuclear receptors and acts as receptor for peroxisome-proliferators, steroids, retinoc acids, and polyunsaturated fatty acids. The current study examined the transcript levels of peroxisome-proliferator activated receptor-gamma (PPARγ) and its co-activator (PGC-1) in a cohort of patients with breast cancer. An invasive breast cancer cell, MDA MB 231 exhibited lower level of expression of PPARγ, compared with non-invasive MCF-7. Both cells expressed PGC-1. Breast cancer tissues (n=120) exhibited a lower level of PPARγ mRNA compared with normal tissues (n=25, p=0.05). However, no difference of PGC-1 was seen between normal tissues and tumour tissues. Although the levels of PPARγ and PGC-1 did not correlate with nodal involvement and grade, significantly lower levels of PPARγ were seen in TNM3 and TNM4 tumours and from patients with local recurrence and those who died of breast cancer. Lowest level of PGC-1 was also seen in TNM3 and TNM4 tumours and patients who died of breast cancer. It is concluded that there is aberrant expression of PPARγ and its co-activator, PGC-1, in human breast cancer, and that low levels of these molecules in cancer tissues are associated with poor clinical outcomes.

© 2003 Cancer Research UK

### P80

HEPATOCYTE GROWTH FACTOR ACTIVATORS (HGFA AND MATRIPTASE-1) AND HGF INHIBITORS IN HUMAN BREAST CANCER. Christian Parr*, Gareth Watkins, Robert E. Mansel and Wen G. Jiang. Metastasis Research Group, Department of Surgery, University of Wales College of Medicine, Cardiff, U.K.

Hepatocyte growth factor (HGF) stimulates cancer cell dissociation, migration, invasion and angiogenesis. This factor is synthesised as an inactive precursor called pro-HGF. HGF activator (HGFA) and matriptase-1 are two serine proteases responsible for converting pro-HGF to active HGF, and have been implicated as pro-invasion and metastasis factors. HAI-1 and HAI-2 possess the ability to inhibit the action of both HGFA and matriptase-1. This study determined the expression of HGFA, HAI-1, HAI-2, and matriptase-1 in breast cancer.

Breast cancer tissue (n=100) and normal background tissue (n=20), was obtained immediately after surgery. The median follow-up for the patients was 72 months. Matriptase-1, HGFA, HAI-1 and HAI-2 expression was determined using RT-PCR and Q-RT-PCR.

HGFA was expressed at a higher level in node positive tumours compared to specimens from patients without nodal involvement (59±124 copies/µl vs. 38± 72 copies/µl, respectively). However, HAI-2 was expressed to a lower degree in node positive tumours (341±368 copies/µl), than that in the node negative breast cancer tissues (440±5685 copies/µl). Our results showed that grade 3 tumours produce statistically lower levels of HAI-1 (821±1183 copies/µl) and HAI-2 (237±358 copies/µl), compared to grade 1 tumours (1898±3800 and 479±522 copies/µl, respectively). HAI-2 was also found to be statistically lower in the TNM 3 breast cancer group when compared to TNM groups 1 & 2 ($p<0.001$), thus associated with a poor prognosis. No significant correlation was found between matriptase-1 and nodal status or tumour grade.

This is the first study to quantitate HGFA, matriptase-1 and HAI expression in human breast cancer tissues. The aberrant levels of HGFA, HAI-1 and HAI-2 have important bearing to the grade and stage of human breast cancer.

### P81

INDUCTION OF INSULIN-LIKE GROWTH FACTOR BINDING PROTEIN-3 BY HYPOXIA AND INCREASED URINARY EXCRETION IN BLADDER CANCER. Jonathan J. Ord* Edward H. Streeter David Cranston Adrian L. Harris Address for correspondence – Room 405 Institute of Molecular Medicine, John Radcliffe Hospital, Headington Oxford. j.ord@cancer.org.uk

Using 10000 gene Sanger cDNA chips we analysed gene expression; in an aggressive bladder cancer cell line EJ28 under normoxia and hypoxia (0.1%); and on 38 bladder cancers compared to a panel of 11 cancer cell lines. One of the most strongly differentially expressed genes in both arrays was the mRNA for Insulin-like growth factor binding protein 3 (IGFBP-3). This was upregulated 6-fold by hypoxia and 2 to 100-fold in 33/38 tumours. Vascular Endothelial Growth Factor mRNA by comparison was upregulated 2-fold by hypoxia and 2 to 18-fold in 27/38.

Urine from 157 bladder cancer patients and controls were collected prospectively and IGFBP-3 levels measured by ELISA. There were 71 superficial cancers, 18 invasive, 4 carcinoma-in situ, 30 patients clear of disease, and 30 controls.

There was a significant increase in urinary IGFBP-3 from control/clear groups to superficial cancers to invasive cancers, $p<0.01$ for superficial vs. control/clear, $p<0.01$ for invasive vs. control/clear [IGFBP-3 ng/ml mean (standard deviation); controls 15.2ng/ml (7.55); clears 14.6ng/ml (8.1); superficial 27.7ng/ml (29); invasive 55.1ng/ml (40)]. After correction for urinary creatinine significance ($p<0.01$) was retained for invasive tumours not with the superficial group.

Previous in vitro experiments on cell growth and IGFBP-3 make it very likely that the levels of IGFBP-3 seen here are of clinical significance reflecting the hypoxia in the primary tumour and contributing to cell viability

### P80a

LOBULAR CANCER- THE MARKER FOR BRCA 3 AND 4?

E F Shah*, L Barr, A D Baildam and D G R Evans
Specialist Breast Unit and Academic unit of Medical Genetics, South Manchester University Hospitals NHS Trust and St Mary's Hospital, Manchester

Aims: To demonstrate the likelihood of lobular carcinoma in individuals with LCIS who have a strong family history of lobular breast cancer, the lack of BRCA1 and 2 mutations in these families and the possibility of a link with other putative breast cancer susceptibility genes such as BRCA 3 and 4.

Procedures: Women attending the family history clinic with a 1:4 or greater lifetime risk of breast cancer were considered eligible for bilateral risk reducing mastectomy. 136 women from different breast cancer families underwent preventative surgery and follow-up thereafter in a dedicated out-patient setting. They went through the phases of a rigorous prophylactic mastectomy protocol[1] prior to surgery. All the families underwent extensive testing for BRCA1 and 2.

Findings: Six patients were found to have LCIS and/or invasive lobular cancer on definitive histology. All of them had a family history of lobular cancer. Testing for BRCA1 and 2 was negative in all cases, including one patient from a family with a BRCA1 mutation, which was discovered later.

Conclusions: LCIS in a BRCA1 or 2 family could be a coincidental finding. Lobular cancer may be the marker for BRCA 3 and 4.

Reference:

1. Lalloo F, Baildam A, Brain A, Hopwood P, Evans DGR, Howell A. A protocol for preventative mastectomy in women with an increased lifetime risk of breast cancer. *Eur J Surg Oncol* 2000; **26:** 711-713.

### P82

ELUCIDATION OF THE MOLECULAR MECHANISMS OF CYTOTOXICITY AND PATHOGENESIS IN NON-SMALL CELL LUNG CANCER (NSCLC) USING GENE EXPRESSION PROFILING

Russell D Petty[*1,2&3], Marianne C Nicolson[2], Keith Kerr[3], Graeme Murray[3]. and Elaina Collie-Duguid[1] [1] Dept of Medicine and Therapeutics, Univ. of Aberdeen, [2] Dept of Oncology, [3] Dept of Pathology, Aberdeen Royal Infirmary, Aberdeen, UK.

A better understanding of the molecular mechanisms of action and resistance to cytotoxic drugs and the mechanisms underlying NSCLC pathogenesis will lead to more effective therapies. Effective anti- cancer therapy involves the selective killing of tumour cells, therefore cell death pathways are of particular interest. Unfortunately the complexity of the molecular abnormalities present has hindered progress. We have used the model of neoadjuvant chemotherapy in NSCLC in conjunction with gene expression profiling (Affymetrix HG-U133A genechips-22283 transcripts) to elucidate the molecular determinants of drug cytotoxicity and pathogenesis in NSCLC. To date tumour:normal paired tissues from 3 patients who have received platinum based neoadjuvant chemotherapy have been profiled. Data has been analysed using Affymetrix MASv5.0 and DMTv3.0. We have identified 2599 genes with significantly altered expression in at least one of the tumours and 768 (401 decreased and 367 increased) with significantly altered expression in all three tumours compared to normal tissue (Mann-Whitney $p<0.05$). This analysis identified 20 cell death associated genes with significantly altered expression in all three tumours- these include TRADD, IAP, DAP-3, DAPK-2, FLAME-1 (average fold change 2.5, 3.0, 1.7, -8.0, and -2.0 respectively). Further work is ongoing and updated results including gene expression profiles in relation to pathological response will be presented at the meeting.

© 2003 Cancer Research UK

### P83

EXPRESSION OF VASCULAR ENDOTHELIAL GROWTH FACTORS (VEGFS), THEIR RECEPTORS AND ANGIONESIS MARKERS (TEMS) IN HUMAN COLORECTAL CANCER

Khaled Rmali*, Malcolm CA Puntis, Wen G Jiang, Department of Surgery, Univ. of Wales College of Medicine, Cardiff, CF14 4XN.

**Introduction**

Angiogenesis is an essential step in tumour growth and metastasis. We studied the expression of VEGFs, their receptors and their connection with angiogenic markers, both specific (TEMs)[1] and non-specific (PECAM) to tumour related angiogenesis in a group of patients with colorectal cancer

**Methodology:** RT-PCR and quantitative RT-PCR were used to assess the expression of VEGF, -B, -C and -D, their receptors Flt-1,KDR and Flt-4, as well as Tumour Endothelial Markers (TEM1-8) and PECAM in tumour (n=21) and normal colon tissues (n=28), and colorectal cancer cell lines (HT115, HRT18 & HT55).

**Results:** VEGF was expressed in HT115, VEGF-B and -D in HT115 and HRT118, and VEGF-C in HT55 & HRT118cells. VEGF-B expression was significantly higher in colon cancer tissues than in normal (p=0.001, $\chi^2$ test). The level of VEGF-C was significantly greater in tumour tissues than in normal (p=0.02), but the level of VEGF and VEGF-D remained the same. While Flt1 and KDR were highly expressed in tumour tissues (p=0.019 and p=0.003 respectively), Flt4 was detected equally both in colonic tumours and in normal (p=0.13). TEM-1, -2, -7, -7R, & TEM8 were at a higher level in colonic cancer than in normal mucosa. No significant difference between the levels of TEM4, TEM6 and PECAM found in cancer and normal mucosal tissues.

**Conclusion:** VEGF-B and VEGF-C, their receptors (Flt1&KDR) and TEM1, 2,7,7R and TEM8 highly expressed in human colorectal cancer tissues, which may contribute to the development of colon cancer, and may constitute a putative target for anti-angiogenic drug therapy.

1. Croix BS, Rago C, *et al*,. Science 2000, 289, 1197

### P84

MUCIN GENE EXPRESSION IN SUPERFICIAL BLADDER CANCER: IDENTIFYING NOVEL MUCIN TUMOUR MARKERS

Sze M. Yong*, Keith N. Stewart, Anne M. Pollock, James N'Dow, Andrew C. Schofield
(Correspondence: Department of Surgery, University of Aberdeen Medical School, Aberdeen, AB25 2ZD.)

**Introduction** Mucins are found in mucus secretions including the urinary bladder and their expression is frequently altered in cancer. We studied the expression of MUC1, 2 and 5AC in carcinoma in-situ (CIS), papillary urothelial carcinomas (pTa) and normal bladder specimens to examine their use as tumour markers in superficial bladder cancer.

**Methods** Samples from 65 bladder cancer patients were investigated including CIS samples (n=25), pTa samples (n=40; Grade1=6, Grade2=30, Grade3=4) and normal bladder specimens (n=14). Immunohistochemistry was performed using antibodies NCL-MUC1, MUC2 and MUC5AC. Specimens were scored for presence or absence, proportion and depth of staining. Statistical analysis was performed using the chi-squared test with the level of significance at p < 0.05.

**Results** MUC1, 2 and 5AC wereexpressed in 96% (24/25), 32% (8/25) and 8% (2/25) of CIS specimens respectively (p<0.001). pTa specimens expressed MUC1 and MUC2 in 92.5% (37/40) and 33% (13/40) respectively, with no expression of MUC5AC (p<0.001). CIS specimens expressed MUC1 in a higher proportion of cells (p=0.004) and stained deeper (p=0.041) compared to pTa specimens. Normal urothelium expressed MUC1 but not MUC2 or 5AC.

**Conclusion** MUC2 and 5AC were expressed in superficial bladder cancer but not in normal bladder specimens. These 2 mucins may therefore be potentially useful tumour markers in superficial bladder cancer.

### P85

*IN VITRO* ACTIVITY OF S-THALIDOMIDE AGAINST MULTIPLE MYELOMA CELLS: A GENE AND PROTEIN EXPRESSION PROFILE.

Jim Malpas*[1,2], Tracy Chaplin[2], Anita Sanmugathasan[2] and Wai Liu[1]. [1]New Drug Study Group and [2]Dept Medical Oncology, St Bartholomew's Hospital, London, UK.

**Background:** Thalidomide (Th) has proven efficacy in multiple myeloma (MM). However, its mode of action is unclear, and it may be anti-angiogenic or may promote apoptosis. We have investigated the changes to the expression of genes involved with these cellular processes following culture with Th in the U266 MM cell line. **Methods:** Cells were cultured with s-Th (0 - 1000 µM: Cellgene Corp., USA), and cell parameters, including apoptosis, were assessed on day 3. RNA was extracted from cells cultured for 24 hr with IC50 of s-Th, and gene expression profiles established by microarray methodologies. **Results:** Reductions in cell viability was observed in U266 cells cultured with s-Th (IC50: 357 µM), which were mirrored by significant increases in apoptosis (day 3: 9.3 ± 0.6% vs. 3.3 ± 0.9% on day 0; p<0.001). The table below shows the changes in expression of the key genes involved with apoptosis (apo) or with angiogenesis (ang).

| **ang** | **VEGF** | **FLT-1** | **β-FGF** | **FGF-r** | **MMP** | **IL-6** | **myc** |
|---|---|---|---|---|---|---|---|
| **Δ(fold)** | 0 | 5 ↓ | 3 ↑ | 0 | 0 | 0 | 10 ↓ |
| **apo** | **p53** | **ras** | **bcl-2** | **bcl-xl** | **TNF-α** | **Iκβ** | **NF-κβ** |
| **Δ(fold)** | 2 ↑ | 10 ↑ | 0 | 0 | 9 ↓ | 29 ↓ | 4 ↓ |

Immunoblotting analyses of treated cells revealed that changes in protein levels did not always correlate with gene expression. As an example, s-Th resulted in a reduction in IL-6 protein level but no change to the gene expression. **Conclusion:** Our data suggests that both angiogenic and apoptotic genes and proteins are affected by s-Th. There is evidence that microarray analyses should not be used alone, as epigenetic factors, not considered by the technique, may eventually alter protein level and functionality.

### P86

EXPRESSION OF TRANSGLUTAMINASES IN HUMAN BREAST CANCER AND THEIR POSSIBLE CLINICAL SIGNIFICANCE.

Wen G. Jiang[1]; Richard J. Ablin[2]; Anthony Douglas-Jones[3]; Robert E. Mansel[1]. [1]Metastasis Research Group and [3]Department of Pathology, University of Wales College of Medicine, Cardiff CF14 4XN, UK; [2]Innapharma, Inc., Park Ridge, NJ 07656, USA.

Implicated in several physiologic processes, including tumour-host responsiveness (Ablin/Gonder. Protides Biol. Fluids, 32:271, 1985), some transglutaminases (TGase) have been noted to play a regulatory role in the extracellular matrix in cell adhesion and migration of cancer cells. This study sought to determine the level of expression of TGases and their possible clinical significance in a cohort of human breast cancer patients (pts.) using RT-PCR and quantitative RT-PCR. Normal breast tissues generally expressed low levels of TGases-1, 2, 3 and 7, and higher levels of TGases-4, 5 and plasma TGase (FXIII). Significantly increased levels of transcripts of TGases-4 and 7, and significantly lower levels of FXIII were seen in tumour tissues (n=110) compared with normal mammary tissues (n=27), p=0,05, 0.04, 0.05, respectively. Node positive tumours exhibited significantly higher levels of TGase-2 and lower levels of TGase-3 (p=0.05 and 0.046, respectively). The lowest levels of TGases-3 and 7 were seen in pts. with metastatic disease, and TGase-3 in pts. who died of breast cancer, compared with those who remained disease free (median follow up 72 months). Higher levels of TGases-4 and 5 were noted in pts. with local recurrence. Breast cancer displays an aberrant expression of TGases, wherein the levels of TGases-2, 3 and 7 have a relationship with node involvement and patient outcome.

© 2003 Cancer Research UK

**P87**

THE EXPRESSION OF IGF-1 IN NORMAL AND MALIGNANT BREAST TISSUE AND ITS ASSOCIATION WITH TUMOUR SIZE

Christiana A Laban*[1], Paul J Jenkins[2], Rob Carpenter[1].
Departments of Breast Surgery[1],and Endocrinology[2], Bart's and The London, London UK.

**Background:** The growth hormone/insulin-like growth factor-I (GH/IGF-I) axis has been increasingly implicated in the development of breast cancer. However the correlation of its local expression with clinical factors has not fully been investigated.

**Aims:**(i) To quantify the mRNA levels of IGF-I in normal and malignant breast tissue and (ii) to identify any correlation between level of expression of IGF-I and clinical parameters.

**Methods**: Total RNA was extracted from 29 samples of paired normal and malignant breast tissue. mRNA levels of IGF-I were quantified using a real time RT-PCR assay ('Taqman'). Full clinical details were available for each patient.

**Results:** (i) IGF-I was expressed in 27 of 29 normal samples and 26 of 29 malignant breast tissue samples. IGF-I expression was significantly downregulated in the malignant tissue compared to normal tissue (median log copy number/microgrammes total RNA 4.8E+06 vs 2.0E+07 respectively; P<0.0005). (ii) IGF-I mRNA expression was significantly increased in the larger cancers (>20mm) compared to smaller cancers (<20mm) (1.2E+07 vs 2.9E+06 respectively; P<0.02). There was no association between IGF-I expression and tumour type, grade, nodal status or ER and PR status.

**Conclusion:** IGF-I mRNA is expressed locally in normal and malignant breast tissue. The upregulation in larger cancers is in keeping with its mitogenic role and suggests it might play an important role in tumour invasiveness and angiogenesis.

**P88**

CHARACTERISATION OF AN ALTERNATIVE TRANSCRIPT OF MURINE B29

*Matthew D. Fox, Mark S. Cragg, and Martin J. Glennie.
Directors Group, Tenovus Research Laboratory, Southampton General Hospital, Southampton, SO16 6YD.

B29 (Igβ, CD79b), and mb-1 (Igα, CD79a) form the CD79 heterodimer, which is crucial for the expression of surface immunoglobulin (sIg), and formation of the B cell Receptor (BCR). An alternative transcript of B29 has been reported in normal and malignant human B cells (hΔB29) where exon 3 is deleted, resulting in a truncated extracellular domain. This transcript appears to regulate apoptosis through the BCR. We have recently discovered a homologous alternative transcript of B29 in normal and malignant murine B cells (mΔB29). Sequence analysis of this transcript confirms that exon 3 has been deleted and that mB29 shares the same splice region as hΔB29. All B cell lines so far assessed express this alternative transcript as judged by RT-PCR and its expression appears to correlate with a diminished sensitivity to apoptosis signalled through the BCR. To address the function of the alternative transcript more directly mΔB29 was cloned into the pCI-puro expression vector. Following transient expression in COS-7 cells and western blot analysis, it was revealed that the mB29 transcript successfully encodes for protein. Furthermore, cellular localisation of mΔB29 prior to and after ligation of the BCR was studied using YFP-tagged mΔB29, indicating that mΔB29 is expressed at the cell surface. Subsequently, various techniques such as over-expression and siRNA have been used to up- or down-regulate expression of mB29, respectively, in a variety of murine B cell lines to directly examine its effect on apoptotic signalling through the murine BCR.

**P89**

TNF-α GENE INDUCTION IN A MODEL OF HUMAN MELANOMA INVASION

David P Jackson, Effie Katerinaki, Paul Berry*, Paul C Lorigan, Sheila MacNeil, Poulam M Patel.
Cancer Research UK Clinical Centre, St. James's University Hospital, Beckett Street, Leeds, LS23 6RZ.

Recent *in vitro* and animal studies suggest that an inflammatory microenvironment may promote melanoma invasion. We recently reported that the invasiveness of the human melanoma cell line HBL through fibronectin is increased by 27% by the pro-inflammatory cytokine TNF-α (1). The aim of this study was to use this model of invasion to investigate TNF-α gene up-regulation in the HBL cell line using cDNA microarrays.

HBL cells were treated with TNF-α at 200 U/ml for 4, 8 or 24 hours. Fluorescent labelled poly A+ RNA, from untreated and TNF–treated cells was hybridised to a 10K cDNA microarray.

Over this time course 21 genes were shown to be up-regulated greater than 2-fold in response to TNF-α and 22 genes were down-regulated. Of these a number of genes have been previously implicated in the TNF-α response, including Glutathione peroxidase and SOD2, scavengers of reactive oxygen species; NF-κB and I-REL, components of the NF-κB transcription complex; and TNFAIPA20. In addition we have identified a number of genes not previously known to be TNF-α regulated.

cDNA microarrays have identified TNF-α regulated genes that may play a role in the TNF-α response. We conclude that a combination of this technology with functional assays assessing the effects of TNF on e.g. cell invasion and migration, applied to a panel of melanoma cell lines with differing invasive potential will be a valuable approach to identifying genes relevant to inflammation associated metastasis.

(1) Zhu N *et al. J Invest Dermatol* 2002; 119:1165-1171

**P90**

**WITHDRAWN**

© 2003 Cancer Research UK

**P91**

GENE EXPRESSION PROFILING OF APOPTOSIS AND CELL CYCLE CONTROL IN DUKES' C COLON TUMOURS. Elaina S R Collie-Duguid[1*], Diane S Stewart[1], James Cassidy[3], Graeme I Murray[2]. Depts of Medicine and Therapeutics[1] and Pathology[2], University of Aberdeen, Aberdeen. CRUK Dept of Medical Oncology, University of Glasgow, Glasgow.

Dukes' C colon cancer patients with potentially curative resection and apparently the same histological stage and grade of tumour have vastly different clinical outcomes. Further insight into the molecular networks controlling tumorigenesis, response to therapy and survival in these cancer patients is required to allow more effective treatment. Cell cycle and apoptotic pathways are commonly disrupted in solid tumours, and this altered cellular profile may support tumour growth and chemoresistance. Microarray technology now allows, the many factors comprising these complex networks to be analysed simultaneously.

Gene expression levels were measured in tumour resection specimens from 4 patients with Dukes' C colon cancer, using Affymetrix HG-U133A GeneChip™ microarrays.

Analysis using Affymetrix MASv5.0 and DMTv3.0, revealed that 862 transcripts were either increased or decreased in all tumours (3.9%). Expression of 15 apoptosis associated transcripts (including MYC, TNFRSF6b, TRAIL-R2 [TNFRSF10b], DAP3 [2.0-3.8 median fold increase]; NFκB1α, Apo2 ligand [TNFSF10], PDCD4 and caspase 7 [1.8-3.8 median fold decrease]) and 33 cell cycle transcripts (including NEK2, DKC1, CDC20, cyclinD1,CDK7, CDC2δT, PCNA, CDC25B, CDK4 and CDKI3 [2.3-15.0 median fold increase]) was altered in all tumours compared to the paired normal tissue (Mann Whitney p<0.05). Expression profiles associated with prognosis and/or chemoresistance are currently being evaluated in a larger patient group.

**P92**

MOLECULAR MECHANISMS OF CYTOTOXICITY AND PATHOGENESIS IN NON-SMALL CELL LUNG CANCER (NSCLC) USING GENE EXPRESSION PROFILING. Russell D Petty*[1], Marianne C Nicolson[3], Keith Kerr[2], Graeme I Murray[2] and Elaina Collie-Duguid[1]. Departments of Medicine and Therapeutics[1] and Pathology[2], University of Aberdeen, Aberdeen, UK; Department of Oncology, Aberdeen Royal Infirmary, Aberdeen, UK[3].

A better understanding of the molecular mechanisms of action and resistance to cytotoxic drugs and the mechanisms underlying NSCLC pathogenesis will lead to more effective therapies. Effective anti-cancer therapy involves the selective killing of tumour cells, therefore cell death pathways are of particular interest. Unfortunately the complexity of the networks controlling chemoresistance and/or tumorigenesis has hindered progress.

We used the model of neoadjuvant chemotherapy in NSCLC in conjunction with gene expression profiling (Affymetrix HG-U133A GeneChips; 22283 transcripts) to elucidate the molecular determinants of drug cytotoxicity and pathogenesis in NSCLC. Tumour:normal paired tissues from 3 patients who received platinum based neoadjuvant chemotherapy were profiled. Data was analysed using Affymetrix MASv5.0 and DMTv3.0 software.

A total of 768 (401 decreased and 367 increased) genes had significantly altered expression in all three tumours compared to the uninvolved tissue (Mann-Whitney p<0.05). This analysis identified 20 apoptosis-associated transcripts with significantly altered expression in all three tumours, including TRADD, IAP, DAP-3, DAPK-2 and FLAME-1. These results illustrate the ability of this technology to unravel the complexity of the molecular abnormalities present. Further work is ongoing to elucidate the gene expression profiles associated with pathological response.

**P93**

QUANTITATIVE RT-PCR TO INVESTIGATE THE EFFECT OF PROTEOLYSIS INDUCING FACTOR (PIF) ON THE EXPRESSION OF UBIQUITIN-PROTEASOME PROTEOLYTIC PATHWAY. Jwan Khal*, Anna V. Hine and Michael J. Tisdale. Pharmaceutical Sciences Research Institute, Aston University, Birmingham, UK, B4 7ET.

Cancer cachexia is characterized by selective depletion of skeletal muscle protein reserves. The ubiquitin-proteasome proteolytic pathway has been shown to be responsible for muscle protein degradation and muscle wasting in cancer cachexia. To establish the importance of this pathway in the action of PIF in inducing protein catabolism in skeletal muscle a quantitative competitive RT-PCR method was developed to measure the mRNA levels of the proteasome subunits C2 and C5 and the ubiquitin-conjugating enzyme $E2_{14k}$. In addition this method was used to determine the effect of the arachidonic acid metabolite 15-hydroxyeicosatetraenoic acid (15-HETE), which is thought to be the intracellular transducer of PIF action. Using a surrogate model system for skeletal muscle, $C_2C_{12}$ myotubes *in vitro*, it was shown that both PIF and 15-HETE increase proteasome subunit expression (C2 and C5) as well as the $E2_{14k}$ enzyme by approximately 74% (p<0.05), 76% (p<0.01) and 63% (p<0.001) respectively, when cells were incubated for 4 hrs with PIF and by 51% (p<0.05), 85% (p<0.05) and 64% (p<0.05), respectively, when treated with 15-HETE for 4 hrs. These effects were attenuated with 50μM EPA, suggesting that EPA may act to inhibit several steps in PIF-induced proteasome expression. These results suggest that 15-HETE is the intracellular mediator for PIF induced protein degradation and the elevated muscle catabolism is accomplished through up-regulation of the ubiquitin-proteasome proteolytic pathway. Furthermore, EPA has shown anti-cachectic properties, which could be used in the future for the treatment of cancer cachexia.

**P94**

ETS TRANSCRIPTION FACTORS IN HUMAN BREAST CANCER

Yvonne. Buggy* [1], Teresa.M. Maguire [1], Gerald. McGreal [1], Arnold.D.K Hill[1+2], Enda. McDermott[1], Niall. O'Higgins[1], Michael.J. Duffy [1+2+3]
[1]Department of Surgery, University College Dublin, St Vincent's University Hospital, Dublin 4, Ireland.
[2]Conway Institute of Biomolecular and Biomedical Research, Dublin 4, Ireland.
[3]Department of Nuclear Medicine, St Vincent's University Hospital, Dublin 4, Ireland

Proteins of the Ets family of eukaryotic transcription factors are involved in a number of fundamental physiological processes, such as organogenesis, haematopoiesis and signal transduction. In particular, specific Ets factors have been implicated in angiogenesis, tumour cell invasion and metastasis.

This study investigated the *ex vivo* expression patterns of four Ets family members, i.e., Ets-1, Ets-2, PEA3/E1AF and ESX in an attempt to establish a link between these factors and the formation and progression of human breast cancer. Following ethical approval messenger RNA levels of all four Ets factors were determined by Reverse Transcriptase Polymerase Chain Reaction (RT-PCR) All four transcripts were increased in tumour tissue compared to either normal breast tissue or fibroadenomas. Transcripts for PEA3/E1AF were not found in normal breast tissue. In the carcinomas, correlations were observed between Ets-1 and HER-2/*neu* and uPA.

In conclusion, this is the first large scale study implicating Ets transcription factors in human breast cancer.

© 2003 Cancer Research UK

**P95**

THE EFFECTS OF ESTRADIOL AND ICI 182,780 TREATMENT OF PRIMARY BREAST CANCER CELLS ON GENE TRANSCRIPTION AND COMPARISON TO EFFECTS IN ESTABLISHED CELL LINES.

Nicola Brookman-Amissah*, Robert K. Price, Richard Foxon, Alan Mackay, Michael J. O'Hare and Robert C. Stein. *Department of Oncology, and Ludwig Institute of Cancer Research, University College London, 91 Riding House Street, London W1W 7BS.*

A gene transcription study of the effects of estradiol (E2) and the steroidal antiestrogen, ICI 182,780, was carried out on primary breast cancer cells. These were isolated from a pleural effusion obtained from a woman with metastatic breast cancer who subsequently responded to endocrine manipulation. After incubation in low estradiol medium for 6 days, the cells were treated with saturating doses of E2, ICI 182,780, E2 and ICI 182,780 in combination or vehicle control. Transcriptional profiling was performed on samples after 4, 24 and 72 hours. cDNA samples from each culture were reciprocally hybridised against reference RNA, from a pool of breast cancer cell lines, to microarrays of 10,000 ESTs representing mostly known cDNAs. Images were analysed using GeneSpring software.

Genes up-regulated by E2 treatment included cytokeratin 15 and the estrogen responsive genes pS-2, cathepsin D and LIV-1. The combination of E2 and ICI 182,780 abrogated these responses. Cells treated with ICI 182,780 alone showed no significant difference from the cells treated with the vehicle control.

Results were compared with data obtained from three estrogen responsive cell lines - MCF-7, T47D and ZR75.1, and an estrogen independent cell line - MDA-MB-453. Preliminary data from T47D cells shows surprisingly little overlap with the primary cells. Further data and validation of the results will be presented.

**P96**

GENE EXPRESSION ANALYSIS USING SERIAL STEREOTACTIC BIOPSIES FROM GLIOMAS

Elisabeth J. Shaw*[+], Michael P.A. Davies[+], Peter C. Warnke[#] and Carol Walker[+]

[+]Clatterbridge Cancer Research Trust, Clatterbridge Hospital, Bebington, Wirral CH63 4JY; [#]Dept. of Neurological Science, Liverpool University, Liverpool.

Gene expression analysis has an important role in the investigation of factors that influence therapeutic response in gliomas. However, minimally invasive surgical procedures, such as serial stereotactic biopsy, limits the tissue available for such study.

In practice, after histopathological diagnosis, 1-6 biopsies per case each containing 70-500ng total RNA may be available for analysis. Expression of the p16 gene relative to the HPRT gene has been successfully demonstrated by RT PCR using 50ng total RNA from such biopsies. However, for microarray analysis RNA amplification is required. The U373 glioma cell line was used for methods development. >From 50ng total RNA, linear amplification by T7 IVT (RiboAmp, Arcturus), cDNA synthesis and indirect labelling gave greater yields than exponential amplification using the Atlas SMART Fluorescence Amplification kit, but showed increased bias toward the 3' end. Hybridisation of Cy3/Cy5 labelled probes onto cDNA microarrays (HGMP) showed reproducible representation of gene expression in experiments using:- different quantities of total RNA in the initial amplification, identical samples processed in parallel, and in self-self hybridisation experiments. RNA from a small series of stereotactic biopsies has been amplified by the T7 IVT method and hybridised on cDNA microarrays and cluster analysis performed.

These results suggest that RNA amplification and microarray analyses may be used to investigate factors that influence response to therapy in gliomas diagnosed by serial stereotactic biopsy.

**P97**

DIFFERENTIAL GENE EXPRESSION IN BLADDER CANCER

Heba Sh. Kassem*, Jonathan H. Shanks, Noel W. Clarke, Richard Cowan, Geoffrey P. Margison

Cancer Research UK Carcinogenesis Group, Paterson Institute for Cancer Research (H.S.K & G.P.M), Christie Hospital, NHS, Trust Manchester, M20 4BX (J.H.S., N.W.C. & R.C.) Pathology Department, Faculty of Medicine, Alexandria University, Egypt (current address of H.K)

mRNA expression patterns were studied in the bladder transitional cell carcinoma (TCC) cell lines RT4, RT112 and T24 that represent the progression model of bladder cancer (G1,G2 and G3, respectively) and 5 paired bladder cancer/non involved mucosa samples using Atlas human oncogene/tumour suppressor cDNA arrays™ (BD Clontech, UK).Adjusted autoradiographic signal intensities were compared using the lower grade cell line or the mucosa as the reference in pair-wise analyses

Twenty-four genes (of 190, 13%) were differentially expressed among the 3 TCC cell lines with a consistent pattern of up or down-regulation in at least 2 comparisons. Fifteen genes (8%) were differentially expressed in the tumours relative to the mucosa counterparts in at least 3 paired-samples.

Comparing the differential expression analysis of the TCC samples and that of the cell lines, some genes revealed a consistent pattern of up or down regulation in the tumour relative to the non-involved mucosa and in the higher grade relative to the lower grade cell lines, indicating their potential role in TCC progression. Differential expression of some genes e.g. EGFR and PCNA, revealed similar patterns of alteration to those previously reported in the literature. Ezrin and neurogenic locus notch protein homologue1 (Notch1) were down-regulated in the high grade cell line (T24) relative to the lower grade cell lines (RT4 and RT112) and also in 4 and 3 tumours relative to the non involved mucosa counterparts, respectively, indicating a potential role in bladder cancer. To the best of our knowledge, this is the first report of the involvement of ezrin and Notch1 in bladder cancer and further analysis is warranted to define their role and potential use as diagnostic and prognostic markers in bladder cancer.

**P98**

DOES THE UP-REGULATION OF GASTRIN AND GASTRIN RECEPTOR GENE EXPRESSION IN BARRETT'S OESOPHAGUS RESULT IN AN INHIBITION OF APOPTOSIS?

*Joseph C. Harris[1], Altaf Awan[1], Janusz Jankowski[2], Susan A. Watson[1]

[1]Cancer Studies Unit, D Floor, West Block, QMC, Nottingham NG7 2UH

[2]Dept. of Cancer Biomarkers, University of Leicester, Leicester

**Introduction:** Barrett's Oesophagus (BE) is a pre-malignant condition of the lower oesophagus characterised by a metaplastic change from squamous to columnar intestinal-type epithelium. This study aimed to identify a role for gastrin,gastrin receptor (CCK2R) and CCK2Ri4sv (splice variant) in the progression of BE via circumvention of apoptosis.

**Methods:** Gastrin, CCK2R and CCK2Ri4sv gene expression was quantified in paired human normal and Barrett's biopsy samples via real-time PCR. Western blotting studies characterised the effect of exogenous gastrin on PKB phosphorylation in three CCK2R positive oesophageal cell lines; OE19 (adenocarcinoma), OE21 (squamous carcinoma)and OE33 (Barrett's metaplasia derived adenocarcinoma).

**Results:** Real-time PCR showed gastrin ($p=0.0076$), CCK2R($p=0.0068$) and CCK2Ri4sv ($p=0.0077$) to be significantly up-regulated in the BE samples compared to paired normals. Exogenous amidated gastrin increased PKB phosphorylation in OE21 and OE33 cells, whilst PKB was constitutively phosphorylated in OE19 cells. Additionally CCK2Ri4sv-transfected OE33 cells showed increased basal PKB phosphorylation compared to wild type cells.

**Conclusion:** Gastrin may play a role in progression to oesophageal adenocarcinoma through its activation of PKB.

© 2003 Cancer Research UK

**P99**

GENE EXPRESSION PROFILING OF SUPERFICIAL BLADDER CANCER. Jeetesh M Bhardwa*, Mahesh Kumar, D M Berney, Joanne Martin, Finbar Cotter, Vinod Nargund.

Bladder cancer (TCC) is the sixth most frequent malignancy occurring worldwide. Superficial TCC accounts for 75-85% of all TCC. 70% of patients with superficial TCC have one or more recurrences after initial treatment, and about one third of these patients have progression of disease. Markers are needed to identify those most at risk of developing invasive disease.

A retrospective study of 128 patients with superficial TCC matched for age/ sex/ smoking habits and number of tumours on presentation were divided into 2 categories, those that had tumours that progressed into invasive disease and those which did not recur nor progress. mRNA extracted from archival material on these patients (n=4) was used to perform Gene expression analysis using the Affymetrix® Human Genome HG U-95A Gene Chip. Out of a total of 12,500 genes on the chip, 10% were called present. We have filtered these genes down to a total of 6 candidate genes, MMP11, ETV6, TFAP2, TNFRSF6B, TGFBIF2, ING1L that were over expressed in TCC that became muscle invasive, other solid tumours are also known to over express these genes There were also 7 genes ACVR1B, CDC2L2, SFN, RBBP8, BECN1, RPS29 and ABL1 that were under expressed in specimens that turned invasive, implying that these genes may have a role in tumour suppression.

We will validate our results by performing Real Time Quantitative PCR on the above-mentioned genes (n=20). We hope to be able to prove that the overexpressed genes play an oncogenetic role in invasive TCC while the under expressed genes are involved in tumour suppression and prevention of muscle invasion.

**P100**

A COMPARATIVE APPROACH TO CANCER CYTOGENETICS
* Rachael Thomas, Ken C. Smith, Matthew Breen
Animal Health Trust, Newmarket, Suffolk UK

The clinical presentation, histology and biology of many canine cancers closely parallels those of human malignancies, and their extensive genome homology is well established. Comparative studies of related human and canine malignancies can make a significant contribution towards the understanding of tumour development in both species, and to improving tools for diagnosis, prognosis and therapy.Our ongoing studies focus on the molecular cytogenetic evaluation of canine cancers, the characterisation of non-random genomic abnormalities, and comparison with knowledge gained from more widely studied human counterparts. Ultimately this will lead to a greater ability to extrapolate data and resources between species, with potential benefit to both human and veterinary medicine.

As an example of this approach, data will be presented on preliminary comparative genomic hybridisation analysis of 25 cases of canine malignant multicentric lymphoma. This represents the most frequent life threatening cancer in dogs, comprising approximately 20% of all canine malignancies. Aberrations involved 32 of the 38 canine autosomes, with a maximum of 12 per case and a mean of three. Genomic gains were almost twice as common as losses. A subset of frequently encountered aberrations was detected. Potential correlations with immunophenotype and histological subtype have been observed. We aim to develop this approach for cytogenetic subdivision of this heterogeneous canine disease, and to correlate findings with clinical outcome. Comparisons may also be drawn with homologous aberrations observed in human lymphoma, suggesting that a related genetic aetiology maybe involved. This will form the framework for more detailed comparative studies.

**P101**

MOLECULAR CYTOGENETIC ANALYSIS OF A COHORT OF MEDULLOBLASTOMAS
Jayne M. Lamont*, Charles S. McManamy, Andrew D. J. Pearson, Steven C. Clifford, David W. Ellison. Northern Institute for Cancer Research, University of Newcastle-upon-Tyne, UK

Although improvements in diagnosis and treatment of medulloblastoma (MB) have increased five-year survival to 50-60%, it is recognised that a clearer understanding of genetic alterations in MB may allow improved treatment stratification and suggest novel therapies. Using fluorescence *in situ* hybridisation, 44 MBs, histopathalogically classified as classic (CL;70.5%), anaplastic/large cell (A/LC;25%) or desmoplastic (DES;4.5%), were analysed for several genetic abnormalities known to occur in MB. 35% (15/43) showed a significant degree of polyploidy at more than 4 of 8 loci (47% being A/LC). Investigation of trisomy 7 indicated copy numbers between 3 and 7+ in 41% (18/44), while 8q24 (*MYC*), showed gain/amplification in 22% of cases. Analysis of 9q22.3 (*PTCH*), revealed 1 gain (DES) and 2 losses (CL and DES), while 10q23.3 (*PTEN*) and 10q24.31 (*SuFu*) showed paralleled losses in 3 cases (7%). Isochromosome 17q (i17q), the commonest aberration in MBs, occurred in 43% of cases and in 55% of A/LC. This is in agreement with previous reports. However, i17q was not preferentially clustered in A/LC MBs, as previously suggested, because 39% of CL MBs also showed this feature. In addition, 5 MBs had 17q gain alone. Although no clear associations between histopathology and molecular cytogenetics are immediately apparent, the results at present do confirm and question previous data. In particular, there is no close association between chromosome 17 abnormalities and the anaplastic phenotype.

**P102**

FUNCTIONAL ANALYSES OF ALTERNATIVE ISOFORMS OF PAX3 IN MELANOCYTES
Qiuyu Wang[1*],Craig Parker[1],Shant Kumar[2], Pat Kumar[1]
1. Manchester Metropolitan University, Chester St., Manchester M1 5GD.

The paired box gene, PAX3 encodes a transcription factor, importantin cell proliferation, migration and survival during early embryonic neural and muscle development. Until recently, five different isoforms had been identified, while their functions remain unknown. We have discovered two new isoforms, g and h, lacking complete coding for exon 8. We have confirmed their sequences and subcloned seven isoforms, PAX3a-h into pcDNA4 expression vector. The constructed plasmids and empty vector pcDNA4 were stably transfected into a non-tumorigenic murine melanocyte cell line, Melan-a, using Transfectam. Transfected colonies wereanalysed for PAX3 isoform expression using specific primers bysemi-quantitative RT-PCR.

Promega Cell Proliferation Assay: melan a-PAX3h cells proliferated more rapidly than mock transfectants over 72h (P<0.01), while Melan a-PAX3a, -PAX3b cells proliferated more slowly (P<0.01 )and Melan a-PAX3c, PAX3d, PAX3e, PAX3g showed similar rates of proliferation. A cell growth curve analysis over 7 days confirmed this, except that PAX3e grew more slowly than mock transfectants. Cell transformation assay: mock transfectants and cells containing PAX3a, PAX3b or PAX3e failed to form colonies in soft agar, whereas cells expressing PAX3c, PAX3d, PAX3g, and PAX3h formed colonies. PAX3d expressing cells formed larger colonies than the others, while cells having PAX3h produced more colonies. Together, our results suggest that alternative isoforms of PAX3 have different effects on melanocyte growth and transformation. Further studies are needed to determine whether there is a causal relationship between PAX3 isoforms and tumorigenesis in melanoma or whether one spliced isoform can regulate another, and to determine the molecular mechanisms involved.

© 2003 Cancer Research UK

**P103**

**WITHDRAWN**

**P104**

ROLE OF MISMATCH REPAIR IN DETERMINING SENSITIVITY TO THE TOPOISOMERASE II POISONS.

Lisa C. Bloodworth*, Linda A.Hogarth, Andrew G. Hall.
Northern Institute for Cancer Research, Medical School, University of Newcastle Upon Tyne, NE2 4HH.

Conflicting evidence exists as to whether mismatch repair (MMR) affects sensitivity to topoisomerase II (topo II) poisons.The topo II gene may be a target for mutagenesis in MMR deficient cells. Drug resistance to topo II poisons *in vitro* has been associated with mutations mainly in 7 exons of the topoIIα gene. To determine if topo IIα is mutated in MMR deficient cells we have screened 12 cell lines of lymphoid and myeloid origin, with known MMR status, for single nucleotide polymorphisms (SNPs) in these 7 exons using denaturing HPLC. Many detected SNPs were intronic. However, in JURKAT and NALM-6 cell lines missense mutations were identified. The JURKAT acute lymphoblastic leukaemia (ALL) cell line showed a novel mutation corresponding to a R815Q amino acid change in the catalytic domain of the protein. The potential functional consequence of this is yet to be determined. MMR proficient and deficient cell lines were compared and there was no significant difference in the number of mutations. 31 relapsed and 35 presentation unpaired childhood ALL samples were analysed for SNPs in the same manner. SNPs detectedwere similar to the cell line screen. However, one relapse sample showed loss of heterozygosity (LOH) at 3 exons which has been confirmed by sequencing. In contrast this was not seen in the presentation and remission samples from the same patient. It is possible that LOH at this locus may be a more common phenomenon in leukaemia than can be detected by dHPLC. Thus further investigation using microsatellite markers is being undertaken.

**P105**

THE USE OF REAL-TIME PCR TO VALIDATE METHYLTHIOADENOSINE PHOSPHORYLASE AND p16 DELETIONS AS A NEW DRUG TARGET IN CHILDHOOD ACUTE LYMPHOBLASTIC LEUKAEMIA

Nadia Thornton*, Andy Hall and Sally Coulthard
Northern Institute for Cancer Research, Medical School, University of Newcastle upon Tyne, NE24HH.

Previous research has shown methylthioadenosine phosphorylase (MTAP), an enzyme involved in purine metabolism, and p16 are frequently co-deleted. As MTAP deficient cells are more sensitive to inhibition of *de novo* purine synthesis, this may offer an important new drug target in childhood acute lymphoblastic leukaemia

A Real-Time PCR assay, which can detect p16 and MTAP co-deletion, has been established in a panel of 28 cell lines, and in childhood ALL patients. Of the 28 cell lines, the MTAP status of 21 cell lines, have not previously been reported.

6/28 (21.4%) of the cell lines had deletions of the MTAP gene, and 7/28 (25%) of the cell lines had a deletion of the p16 gene. Of these, 27/28 (96.4%), had both MTAP and p16 co-deleted, and in only 1/28 (3.6%), p16 deleted but MTAP non-deleted. Of 5 patients studied so far, none have shown deletions in either loci.

Although deletions have been shown in a panel of cell lines, and not in the patients so far, this is most likely due to the small number of patients studied. Real-Time PCR is a robust and reliable method to establish the MTAP and p16 status of cell lines, which can be applied to clinical material.

**P106**

POTENTIAL USE OF ASPARAGINE SYNTHETASE LEVELS IN THE INDIVIDUALISATION OF L-ASPARAGINASE THERAPY

Mark Leslie*, Marian C. Case, Andrew G. Hall, Sally A. Coulthard. Leukaemia Research Group, Northern Institute of Cancer Research, University of Newcastle upon Tyne, U.K.

L-asparaginase (l-asp) is a potent chemotherapeutic agent used in the treatment of acute lymphoblastic leukaemia (ALL). As it is associated with potentially severe side effects, it would be beneficial for treatment to be targeted to patients who will respond. Understanding the molecular pharmacology of this drug will be advantageous in the individualisation of l-asp.

Cytotoxicity of L-asp results from the depletion of circulatory asparagine. Lymphoblasts lack activity of asparagine synthetase (AS) compared with normal cells. Resistance to L-asp is due to increased cellular AS activity in lymphoblasts.

Although lymphoblast AS levels are generally low at presentation, some patients may express higher levels. These patients might not benefit from L-asp. Some patients with acute myeloid leukaemia (AML) have also responded favourably to L-asp; limited results have been published regarding AS expression in these patients.

Using real-time PCR, we have observed large variation in normalised AS mRNA levels (RAS) in ALL and AML. For ALL, RAS values vary from 0.013 to 1.55 (median=0.18, n=68) and AML 0.2 to 11.6 (median=0.72, n=20). Little variation in expression was detected in mononuclear cells from healthy individuals (0.29 to 0.70, median=0.42, n=14). These results reached significance (one way ANOVA, $P<0.0001$); mononuclear v ALL (SNK, $P<0.01$); mononuclear v AML (SNK, $P<0.05$); ALL v AML (SNK, $P<0.001$). These results may have implications for patients being treated with L-asp.

© 2003 Cancer Research UK

**P107**

IDENTIFICATION OF TUMOUR SUPPRESSOR LOCI ON CHROMOSOME 10q IN MEDULLOBLASTOMA

Debbie K. Scott*, Janet C. Lindsey, Meryl E. Lusher, Andrew D.J. Pearson, David W. Ellison and Steven C. Clifford.
Northern Institute for Cancer Research, University of Newcastle, Newcastle-upon-Tyne, U.K.

We have previously demonstrated that deletions involving the long arm of chromosome 10 (10q) are a frequent event in medulloblastoma (MB), the most common malignant brain tumour of childhood. To map these deletions in detail and identify potential MB tumour suppressor gene (TSG) loci, we performed deletion mapping analysis on eleven MB cell lines using a panel of polymorphic markers at 10cM intervals across chromosome 10.

This analysis revealed two key findings. Firstly, that the highest rates of homozygosity were observed at 10q24.3-10q26.1, indicative of a common region of deletion. Secondly, a homozygous deletion involving the 10q23 region was detected, indicative of a MB TSG locus. Detailed mapping of this locus delineated a ~200kb deletion encompassing the *PTEN* TSG and further novel predicted transcripts. *PTEN* coding region mutations are rare in MB, occurring in <5% of tumours. We therefore investigated epigenetic *PTEN* inactivation in a series of MBs. Methylation-specific PCR analysis revealed tumour-specific hypermethylation of the *PTEN* CpG island in 38% (16/42) primary tumours. Work is currently underway to determine whether this is associated with transcriptional silencing and gene inactivation.

These data indicate (i). that the *PTEN* TSG and/or genes at 10q24.3-10q26.1 may represent critical targets of 10q deletions in MB, and (ii). that *PTEN* hypermethylation is a common event in this disease.

**P108**

EPIDEMIOLOGICAL EVIDENCE FOR AN INFECTIOUS ORIGIN FOR CHILDHOOD LEUKAEMIA

Richard J.Q. McNally*, Osborn B. Eden, Jillian M. Birch.
*Cancer Research UK Paediatric and Familial Cancer Research Group, Stancliffe, Royal Manchester Children's Hospital, Hospital Road, Manchester M27 4HA.

The Manchester Children's Tumour Registry (MCTR) is population-based and has been collecting case data from a geographically defined region of North West England since 1954 with almost total ascertainment. The aim of the report is to interpret the results of several recent analyses of childhood leukaemia data from the MCTR to test current hypotheses posing an infectious element of the aetiology of leukaemia. Trend tests, space-time clustering (Knox and K-function tests), ecological regression and geographical mapping were used to study the data on all leukaemias broken down by sub-type into ALL and ANLL. For the most recent period since 1980 ALL was further examined by immunophenotype. There was an increasing incidence of ALL due mainly to precursor B-cell sub-type. Space-time clustering was evident for cases of ALL aged 0-4 years for the time period 1954-85 based on date of diagnosis and place of birth. For the time period 1980-2001 space-time clustering was confined to cases of precursor B-cell ALL aged 18-54 months based on date and place of birth. The incidence of ALL was greater in more densely populated wards due to increased incidence of non-precursor B-cell ALL. The studies present a complex picture, but are supportive of an infectious element in aetiology particularly for precursor B-cell ALL. However there may be at least two infectious mechanisms operating, one predominating in the earlier years of the study period and another in the later years of the study period.

**P109**

GLUT-1, A MARKER FOR TUMOUR HYPOXIA, IS EXPRESSED IN CHILDHOOD NEUOROBLASTOMA AND RHABDOMYOSARCOMA

Edward J Estlin*, Marie Grady, Anna Kelsey, Ian Stratford, Rachel Airley
*Department of Paediatric Oncology, Royal Manchester Children's Hospital, Pendlebury, Manchester M27 4HA, UK

In several adult cancers, tumour hypoxia is associated with a poorer prognosis, possibly as a result of the induction of hypoxia-regulated genes. The facilitative glucose transporter Glut-1, which promotes a compensatory switch to anaerobic glycolysis and adaptation to hypoxia, is a validated surrogate marker for tumour hypoxia. However, there is a paucity of information regarding the prevalence and influence of hypoxia in paediatric cancers. To address this, expression of the hypoxia-regulated gene Glut-1 and the relationship with the prognostic indicators such as stage and MYCN amplification, are currently being investigated in studies involving patients with paediatric neuroblastoma and rhabdomyosarcoma. In a preliminary study, Glut-1 protein expression was investigated in archival formalin-fixed, paraffin-embedded biopsy material from a series of 20 patients presenting with each of neuroblastoma and rhabdomyosarcoma. For neuroblastoma samples, Glut-1 was present in 10/20 cases. Although in this small series of patients, there was no apparent link between lack of survival and either Glut-1 or MYCN amplification, there were non-significant trends whereby Glut-1 was associated with MYCN amplification ($r = 0.316$, $P = 0.201$) and patients showing concurrent Glut-1 expression and MYCN amplification were less likely to have survived ($r = 0.329$, $P = 0.183$). Glut-1 expression was found in 30% of rhabdomyosarcoma cases. Upon examination, virtually all tumours had large areas of abundant vascularisation. However, Glut-1 expression, if present, mostly occurred in patches of poor vascularisation around areas of necrosis. In conclusion, Glut-1 expression may be an indicator of hypoxia in paediatric neuroblastoma and rhabdomyosarcoma. Further study of an expanded series of patients for each tumour type is planned to define the relationship between Glut-1 expression and tumour stage, biology and outcome.

**P110**

SUSTAINED ACTIVATION OF RAS AND THE MAP KINASE P38 ARE EFFECTORS OF BASIC FIBROBLAST GROWTH FACTOR (bFGF)-INDUCED DEATH IN THE EWING'S SARCOMA FAMILY OF TUMOURS (ESFTs).

Andrew J. K. Williamson*, Ben C. Dibling and Susan A. Burchill.
Cancer Research UK, St James's Hospital, Leeds, LS9 7TF.

The aims of this study were to examine the effects of bFGF on cytoskeletal organisation and to determine whether bFGF-induced cell death was initiated following activation of the MEK-ERK and p38 pathways. Phalloidin and confocal microscopy were used to examine the effect of bFGF on the organisation of the cytoskeleton. Cell cycle profile was examined by fluorescent associated cell sorting (FACS) and Western blot. Expression and activation of Ras, ERK and p38 were examined by Western blot, ELISA, immunoprecipitation and GST- Ras binding domain binding assays. The effect of bFGF in the presence and absence of 10 μM PD98059 (ERK inhibitor) or 20 μM SB202190 (p38 inhibitor) on cell death was examined by FACS. TC-32 cells accumulated in G1 and produced neurite-like extensions after 24 hours exposure to bFGF. However A673 cells, which do not die, did not. Exposure of TC-32 cells to bFGF induced rapid and sustained phosphorylation of Ras, ERK and p38. Phosphorylation was seen five minutes after addition of bFGF and was sustained up to two hours. Sustained activation of p38 MAPK was extended to seventy-two hours. Incubation of TC-32 cells with PD98059 or SB202190 rescued TC-32 cells from bFGF-induced cell death, suggesting that ERK and p38 play a role in the signalling for cell death following exposure to bFGF. However ERK activation was also sustained in A673 cells, although Ras and p38 were not activated. This suggests that sustained activation of p38 and/or Ras are the major effectors of bFGF induced cell death in ESFT cells.

© 2003 Cancer Research UK

**P111**

OXIDATIVE METABOLISM OF 13-CIS-RETINOIC ACID IN CHILDREN WITH NEUROBLASTOMA

Gareth J. Veal*, Julie Errington, Karin E. Koller, Ann M. Elsworth, Andrew D.J. Pearson and Alan V. Boddy on behalf of the UKCCSG Pharmacology Working Group. Northern Institute for Cancer Research, University of Newcastle upon Tyne, Newcastle upon Tyne NE2 4HH.

13-cis-retinoic acid (13cisRA) plays a key role in the treatment of high-risk neuroblastoma (nbl) in children. However, whereas a high-dose intermittent schedule of13cisRA has proved to be clinically beneficial, a lackofefficacy was observed with low-dose continuous 13cisRA, suggesting that 13cisRA systemic exposure may be a key factor in determining clinical efficacy. In addition, oxidative metabolism of the retinoids has previously been shown to be clinically important, with ATRA metabolism being correlated with drug resistance in acute promyelocytic leukaemia.

We are investigating the pharmacokinetics of 13cisRA in nbl patients receiving a dose of 80 mg/m$^2$ b.d. (14 days consecutively out of 28; 6 courses). 10 patients have currently been studied with pharmacokinetic sampling carried out at 0,1,2,4 and 6 hrs post- 13cisRA administration on days 1 and 14 of treatment. Retinoid concentrations in plasma were determined by HPLC.

Peak 13cisRA concentrations of 0.4-9.2μM were observed between 1 and 6hrs in patients studied on course 2, with an approximate 15-fold inter-patient variation in exposure. Extensive accumulation of 4-oxo-13cisRA occurred between days 1 and 14, with plasma concentrations of this metabolite being greater than those of 13cisRA by day 14 in 7/10 patients. Metabolite concentrations returned to baseline on day 1 of each treatment course. These data show significant inter-patient variation in 13cisRA exposure and demonstrate that intermittent scheduling of 13cisRA limits the accumulation of its oxidative metabolites.

**P112**

GLUTATHIONE IN CHILDHOOD ACUTE LYMPHOBLASTIC LEUKAEMIA (ALL)-PREDNISOLONE RESISTANCE AND RATE OF BLAST PROLIFERATION-DOUBLE TROUBLE?

G. A. Amos Burke*, Sarah J. Townend, Dharmesh B. Patel, Nicholas J Goulden, Pamela R. Kearns.
Department of Child Health, School of Medical Sciences, University of Bristol, UK

Raised blast glutathione (GSH) levels in childhood ALL are correlated with high presenting white cell counts (WCC) and an increased risk of relapse but the relationship to drug resistance remains unclear. Using the T lineage leukaemia cell line CCRF-CEM C7, we demonstrate that increased total GSH is associated with resistance to prednisolone and that GSH levels are related to proliferation rates *in vitro*.

Total GSH levels were measured using the recycling assay of Tietze. *In vitro* cytotoxicity was assessed using the sulforhodamine B (SRB) colorimetric assay and proliferation was measured using incorporation of $^3$H thymidine.

Cells grown in the absence of serum had 2-fold higher GSH levels (34.9 v 17.6; p<0.001) and peak GSH concentrations were related to peak incorporation of $^3$H thymidine. Higher GSH content resulted in decreased sensitivity to prednisolone (p<0.05). Reducing GSH levels in serum-free culture using buthionine sulfoximine resulted in partial restoration of prednisolone sensitivity (p <0.05 at effective dose combinations). Paradoxically, 10 mM *N*-acetylcysteine increased the sensitivity to prednisolone.

Our data supports the concept that redox status is one of the molecular determinants of prednisolone sensitivity with the potential for therapeutic modification. The correlation of GSH with rate of proliferation offers an explanation for the association with high WCC at presentation in children with ALL.

**P113**

A SURVEY OF CURRENT PRACTICE IN UK PAEDIATRIC ONCOLOGY CENTRES AND ASSISTED CONCEPTION UNIT FOR THE PRESERVATION OF FERTILITY FOR ADOLESCENT MALES WITH CANCER.

Satinder Jagdev[1*], Linda Phelan[1], Marilyn Crawshaw, Juliet Hale, Adam Glaser[1]. [1]Paediatric and Adolescent Oncology Unit, St James' University Hospital, Leeds.

The treatment of cancers in childhood and adolescents may lead to impaired fertility. The offer of fertility preservation techniques for adolescent males has been a relatively new service. This postal survey(funded by NHS Executive) was conducted to establish current practice in the UK. Questionnaires were sent to 22 paediatric oncology(POCs) centres and 105 assisted conception units (ACUs) across the UK with 90% and 62% response rates respectively. Of the 20 responding POCs, 100% offered access to storage facilities for sperm and/or testicular tissue. The techniques offered included ejaculation (100% of centres), electro-ejaculation (35%), epididymal aspiration (30%) and testicular biopsy (30%). Only 40% of POCs had access to written guidelines specifically for adolescent males. The commonest tumour types in which fertility preservation was offered in both settings were Hodgkin's and Non-Hodgkin's Lymphoma and sarcomas. Of 65 responding ACUs, 23 offered storage facilities to adolescent males. The techniques offered included ejaculation (95%), electro-ejaculation (26%), epididymal aspiration and testicular biopsy(69% respectively). 75% of POCs and 74% of ACUs required their adolescent male patients to be Gillick competent to undergo sperm banking. Fertility counselling and psychosocial support were provided by a variety of health professionals in both settings. These data highlight the inconsistencies in information and access to services for adolescent males with cancer. Specifically tailored national guidelines, are needed.

**P114**

HOW SHOULD GLOMERULAR FILTRATION RATES BE MEASURED IN TEENAGERS HAVING CHEMOTHERAPY?

Jeremy Whelan,* Caoimhe O'Sullivan, Susie Stanway and Siew Ng

Accurate assessment of renal function of patients receiving intensive chemotherapy is essential. Glomerular filtration rate (GFR) may be assessed by EDTA clearance or may also be derived from formulae using serum creatinine values. Isotopic EDTA clearance is the gold standard for measuring GFR but is invasive, slow and uses radio-isotopes. The formulae most appropriate for use with teenagers has not yet been determined. The aim of this study was to compare three commonly used formulae (Schwartz, Modified Schwartz and Cockcroft-Gault) to EDTA in thirteen to twenty-one year olds having chemotherapy.

*Methods*: EDTA clearance results from 114 patients were obtained from the nuclear medicine department. The three calculations were then performed and compared to the EDTA clearance.

*Statistical analysis:* Agreement between the three methods was assessed. Sensitivities and specificities were computed for each method, with GFR rates of less than 80 units considered as the cut-off point as this value is commonly used for a dose reduction or exclusion from a regimen.

*Results:* We found low agreement and large variation in scores relative to their corresponding EDTA. The sensitivities were 9% (95% CI: 3% to 22%), 37% (95% CI: 23% to 53%) and 2% (0% to 12%) for the Schwartz, Modified Schwartz, Cockcroft-Gault formulae respectively. In contrast, the specificity of each formula was high.

*Conclusion*: The poor agreement and the low specificity and high sensitivity of the Schwartz, Modified Schwartz, Cockcroft-Gault formulae compared to the EDTA measurements suggest that these methods may not be adequate for correctly identifying 13-21 year olds with reduced renal function.

© 2003 Cancer Research UK

**P115**

A CRYPTIC REARRANGEMENT INVOLVING *MLL* AND *MLLT10* OCCURRING *IN UTERO*.

Louise K. Jones*[1], Michael J. Neat[2], Frederik W. van Delft[1], Michael P. Mitchell[3], Maria Adamaki[1], Sara J. Stoneham[1], Naina Patel[1] and Vaskar Saha.[1]
Cancer Research UK Children's Cancer Group[1] and Medical Oncology Unit[2], 3[rd] Floor, John Vane Science Building, Barts and The London School of Medicine and Dentistry, Queen Mary University of London, Charterhouse Square, London, EC1M 6BQ.

We describe a child with a diagnosis of Acute Myeloid Leukaemia (AML) FAB 5, who presented initially with a prodromal phase. Routine cytogenetic G-banding identified a dic (1;19). Fluorescent *in-situ* hybridisation, however, identified a cryptic insertion of *MLL* into 10p12. RT-PCR and sequence analysis confirmed the presence of a *MLL-MLLT10* fusion, resulting in an in-frame fusion of *MLL* exon 6 to *MLLT10* 979. Using long range PCR, 21kb of intronic sequence was analysed. The precise genomic breakpoint was localised to intron 6 of *MLL* (bp 1512) and *MLLT10* bp 10884 (between coding sequence 978/979). An identical genomic fusion was identified by PCR and sequence analysis from DNA extracted from the neonatal Guthrie blood spot.

Sequence analysis shows both introns to contain 70% repetitive elements. Junctional analysis of the genomic fusion shows that the breakpoint is immediately flanked by an *Alu*Jb repeat on the *MLL* side and *L1*MCa repeat on the *MLLT10* side and contains short sequence homologies, an indication of errors in end-joining of double strand breaks.

This is the first report of a *MLL-MLLT10* fusion at birth and provides further evidence of the prenatal origin of chromosomal translocations seen in childhood AML.

**P116**

SELECTION FOR p53 MUTANT CELLS BY CHEMOTHERAPY IN HIGH-RISK NEUROBLASTOMA

Jane Carr*, Christine Challen, Julian Board, Katrina M. Wood, Andrew D. J. Pearson, John Lunec and Deborah A. Tweddle
Northern Institute for Cancer Research, Medical School, University of Newcastle, NE2 4HH.

**Background:** Neuroblastoma is the most common cause of death from childhood cancer. Most high-risk neuroblastomas (stage 4 >1 yr, or *MYCN* amplified localised & infant disease) initially respond to cytotoxic therapy, but the majority relapse with chemoresistant disease. p53 mutations are rare in neuroblastoma at diagnosis, however they have been occasionally reported in relapse tumours and cell lines, and are associated with chemoresistance (Tweddle *et al*, Cancer Res, 2001:61:8-13).

**Methods:** Automated DNA sequencing was used to sequence exons 4-9 of the p53 gene in 8 paired diagnostic and relapsed neuroblastomas. Four out of 8 cases were high-risk neuroblastoma and 6 patients died of progressive disease.

**Results:** A p53 mutation was detected in the relapse but not the diagnostic tumour in 1 of the 6 patients who subsequently died fromprogressive disease. This was a missense mutation at codon 270 causing amino acid change from phenylalanine to leucine in a patient with high-risk (stage 4 >1yr) *MYCN* amplified neuroblastoma. The same mutation was identified in DNA from tumour after less than 3 months of induction (rapid COJEC) chemotherapy. Using mutation specific p53 primers we were unable to amplify DNA from the tumour at diagnosis. The minimum concentration of mutant DNA required to generate a PCR product was 0.05%, suggesting that if the mutation was present at diagnosis then it was at a lower level than this.

**Conclusions:** These findings are consistent with selection of p53 mutant cells by chemotherapy in a subset of high-risk neuroblastomas and may have important therapeutic implications.

**P117**

EVIDENCE FOR A REDOX MECHANISM OF ACTION OF PREDNISOLONE IN CHILDHOOD ACUTE LYMPHOBLASTIC LEUKAEMIA THROUGH THE IDENTIFICATION OF THE NOVEL GENE *CGI-31*.

G. A. Amos Burke*, Pamela R. Kearns, Nicholas J. Goulden.
Department of Pathology and Microbiology, School of Medical Sciences, University of Bristol, UK

Glucocorticoids are the most important drugs used in the treatment of acute lymphoblastic leukaemia and poor response to these drugs during induction is a powerful adverse prognostic factor. However, the mechanisms by which glucocorticoids induce cytotoxicity are poorly understood. Using the T-lymphoblastic cell line CCRF CEM C7, we have demonstrated the involvement of a novel gene with proposed thioredoxin function in the response to the glucocorticoid, prednisolone.

Global gene expression profiles were examined in sensitive and resistant populations of CCRF CEM C7 using the technique of differential display. Quantitative RT-PCR was used to confirm altered gene expression.

Using differential display, down-regulation of the novel gene *CGI-31* was seen in sensitive but not resistant leukaemia cells during 6 hours of prednisolone exposure and this was confirmed using quantitative RT-PCR.

The involvement of *CGI-31*, a gene with proposed thioredoxin function, in the response to prednisolone of sensitive leukaemic cells supports the concept that prednisolone acts via a mechanism involving changes in the redox status of the cell. We believe this novel mechanism offers the potential for the development of new therapies. Studies with RNA interference are ongoing to assess the functional significance of *CGI-31*.

**P118**

HIERARCHICAL CLUSTERING ANALYSIS OF GENE EXPRESSION PROFILES ACCURATELY IDENTIFIES SUBTYPES OF ACUTE CHILDHOOD LEUKAEMIA

Frederik van Delft*, Tony Belloti, Mike Hubank, Tracy Chaplin, Naina Patel, Maria Adamaki, Daniella Fletcher, Ian Hann, Louise Jones, Alex Gammerman, Vaskar Saha. CRUK Children's Cancer Group, Charterhouse Sq, London EC1M.

**Aims:** Devising mathematical tools for diagnosing acute leukaemia subtypes, based on data obtained from gene expression profiling.

**Procedures:** Bone marrow cells were obtained from all patients newly diagnosed to have acute leukaemia at the Royal London and Great Ormond Street Hospitals from February 2002. RNA was extracted immediately from the mononuclear fraction and gene expression analysis performed using the HGU133 A and B gene chip. Two supervised hierarchical clustering techniques were used to identify the minimum number of genes able to discriminate leukaemic subtypes.

**Findings:** Data was refined on 30 ALL and 10 AML patients. A 30 gene set, cross validated between the two methods, was identified. Gene expression patterns were validated using Real-Time PCR. This was able to accurately differentiate the different leukaemic subtypes including immunophenotype and cytogenetic changes. This gene set was then used to accurately identify two cases of unclassified leukaemia. Preliminary analysis also identifies similarities and diversities within specific subgroups.

**Conclusion:** Gene expression analyses provide a rapid and single platform for the diagnosis of haematological malignancies. Future correlation with response to treatment and outcome has the potential to further refine risk-classification and identify novel pathways for targeted therapy.

© 2003 Cancer Research UK

